# The ‘Maltreatment and Abuse Chronology of Exposure’ (MACE) Scale for the Retrospective Assessment of Abuse and Neglect During Development

**DOI:** 10.1371/journal.pone.0117423

**Published:** 2015-02-25

**Authors:** Martin H. Teicher, Angelika Parigger

**Affiliations:** 1 Department of Psychiatry, Harvard Medical School, Boston, Massachusetts, United States of America; 2 Developmental Biopsychiatry Research Program, McLean Hospital, Belmont, Massachusetts, United States of America; 3 Department of Psychology, University of Konstanz, Konstanz, Germany; Central Institute of Mental Health, GERMANY

## Abstract

There is increasing interest in childhood maltreatment as a potent stimulus that may alter trajectories of brain development, induce epigenetic modifications and enhance risk for medical and psychiatric disorders. Although a number of useful scales exist for retrospective assessment of abuse and neglect they have significant limitations. Moreover, they fail to provide detailed information on timing of exposure, which is critical for delineation of sensitive periods. The Maltreatment and Abuse Chronology of Exposure (MACE) scale was developed in a sample of 1051 participants using item response theory to gauge severity of exposure to ten types of maltreatment (emotional neglect, non-verbal emotional abuse, parental physical maltreatment, parental verbal abuse, peer emotional abuse, peer physical bullying, physical neglect, sexual abuse, witnessing interparental violence and witnessing violence to siblings) during each year of childhood. Items included in the subscales had acceptable psychometric properties based on infit and outfit mean square statistics, and each subscale passed Andersen’s Likelihood ratio test. The MACE provides an overall severity score and multiplicity score (number of types of maltreatment experienced) with excellent test-retest reliability. Each type of maltreatment showed good reliability as did severity of exposure across each year of childhood. MACE Severity correlated 0.738 with Childhood Trauma Questionnaire (CTQ) score and MACE Multiplicity correlated 0.698 with the Adverse Childhood Experiences scale (ACE). However, MACE accounted for 2.00- and 2.07-fold more of the variance, on average, in psychiatric symptom ratings than CTQ or ACE, respectively, based on variance decomposition. Different types of maltreatment had distinct and often unique developmental patterns. The 52-item MACE, a simpler Maltreatment Abuse and Exposure Scale (MAES) that only assesses overall exposure and the original test instrument (MACE-X) with several additional items plus spreadsheets and R code for scoring are provided to facilitate use and to spur further development.

## Introduction

A variety of tools have been developed for use in adults to retrospectively assess exposure to childhood maltreatment. One of the most widely used instruments is the Childhood Trauma Questionnaire (CTQ) [1,2], a 28-item self-report inventory that provides a brief, reliable, and valid screen for histories of abuse and neglect. It assesses severity of exposure to five types of maltreatment—emotional, physical, and sexual abuse, and emotional and physical neglect. Scores on each section range from 5–25, and can be summed to a composite score. A more recent self-report scale of similar design is the Childhood Experience of Care and Abuse questionnaire (CECA-Q) [3] based on the Childhood Experience of Care and Abuse Interview (CECA; [4]). The self-report questionnaire assesses lack of parental care (neglect and antipathy), parental physical abuse, and sexual abuse from any adult before age of 17, and shows satisfactory reliability and validity. Another important and even simpler metric is the Adverse Childhood Experience (ACE) score [5], which emphasizes multiplicity of exposure rather than severity. The ACE score was derived from 18 questions adapted primarily from the Conflict Tactics Scale [6] about the first 18 years of life. Three categories of childhood abuse were assessed: emotional, physical, or contact sexual abuse, along with five categories of household dysfunction: exposure to substance abuse, mental illness, violent treatment of mother or stepmother, incarceration for criminal behavior and parental separation, divorce or death [5,7]. Subjects were defined as exposed to a category if they responded “yes” to one or more of the questions in that category. The number of categories reported (range 0–8) was summed to produce the ACE score [5,7]. This turned out to be a simple, but highly effective strategy, yielding a multiplicity score that seems to capture the extent of exposure to childhood adversity. The most recent version of the ACE score (Wave II) added two categories—physical and emotional neglect [8].

The ACE score was used in an important series of epidemiological studies assessing the relationship between childhood adversity and measures of physical and mental health in a Health Maintenance Organization population of 17,337. ACE scores were found to be a powerful determinant of risk for alcoholism [9,10,11], substance abuse [12], depression [5,9], suicidality [13], and receipt of psychotropic medications [7,14] and to delineate a graded ‘dose-dependent’ relationship between early adversity and outcome. Evidence of a dose-dependent relationship lends further credence to a causal link and provides a critically important statistical insight. Individuals with ≤ 5 ACEs (versus those with 0 ACEs) were 3.7–5.0 fold more likely to have a lifetime history of depression, and 4.4–6.4 fold more likely to have had a recent episode of depression [15]. Individuals with very high levels of exposure to adversity (ACE ≢ 7) were 31-fold more likely to have attempted suicide. The population attributable risk fractions associated with 1 or more ACEs were 67%, 64% and 80% for lifetime, adult, and childhood/adolescent suicide attempts, respectively. This graded relationship between ACE score and psychopathology was also reflected in pharmacy records. Adults with ≥ 5 ACEs (versus those with 0 ACEs) had 3-, 2-, 10- and 17-fold higher rates of prescriptions for antidepressant, anxiolytic, antipsychotic and mood-stabilizing medications [7]. Overall, early adversity as indexed by the ACE score accounted for 50–75% of the population attributable risk fraction associated with depression, suicide attempts, drug abuse and alcoholism, which are leading causes of premature death and disability in the USA [16].

However, while the ACE has been particularly useful in epidemiological studies given its brevity, and the CTQ has served well as both a clinical assessment instrument and a primary research tool, they each have their limitations. Our primary concern is that three ACE score items appear to confound an aversive early experience with shared inheritance. These ACE items are the presence of family members in the household with: (1) substance abuse, (2) chronic mental illness or (3) criminal behaviors leading to incarceration, which is often directly or indirectly related to substance abuse. These items do not focus on what the subject actually experienced, and could simply be indicative of an enhanced genetic risk for substance abuse [17] or major mental illness [18]. This problem with the ACE score has been recognized by the authors, as they have, for example, conducted a separate analyses of ACE scores minus the family mental illness item when examining the effects of childhood adversity on prescribed psychotropic drug use [7]. We felt that it was critical to minimize this confound particularly as we sought to better understand how genetic risk and exposure interacted. Second, the ACE, CTQ and CECA-Q omit forms of maltreatment that we believe to be important. None of these instruments solicits information about exposure to peer victimization. Bullying is a major risk factor for psychopathology and drug abuse [19,20,21,22,23,24,25], with the main determinant being exposure to peer emotional maltreatment [25]. Further, the CTQ and CECA-Q contain no items about witnessing domestic violence and the ACE only inquires about witnessing violence toward mothers or stepmothers. We have recently reported that witnessing violence towards siblings occurs as often as witnessing violence toward mother and actually appeared to be of substantially greater impact regarding risk for psychopathology [26].

Finally, a limitation of all instruments used in adults to retrospectively assess exposure to childhood maltreatment is that none collect detailed information on how exposure levels changed across development. This information is of fundamental importance as there may be sensitive periods when experience exerts maximal effects on the developmental trajectory of specific brain regions [27,28] and risk for psychopathology [29,30]. Hence, our goal was to develop a self-report instrument for use in adults to assess exposure to childhood maltreatment that included items for peer victimization, witnessing violence toward mothers, fathers and siblings, eliminated items that could confound exposure with familial risk, and collected information on exposure at each age. Our aim was to be able to provide both an ACE-like multiplicity of exposure score and a CTQ-like severity score across development.

In planning to develop this instrument we decided to select items for inclusion using item response theory (IRT). Rating scales in psychiatry and clinical psychology have traditionally been developed and evaluated using classic test theory (CTT). However, some recent efforts have been made to develop new scales or revise older scales using IRT (e.g., [31,32,33]). CTT analyzes and evaluates an instrument in regard to the total test score (e.g., number of endorsed items) and is based on a theory of errors in measurement that explain differences between the observed score and the person’s true score. CTT does not analyze the specific properties of test items, and highly similar items are often included in CTT-developed scales that use Chronbach’s alpha and split-halves reliability as key criteria.

In contrast, IRT focuses on the principle that discrete response items are taken to be observable manifestations of hypothesized latent traits, constructs, or attributes that can only be inferred from the manifest response. Further, response items differ on one or more properties. In the simplest IRT, the Rasch model, the probability that an item is endorsed is a function of the person (e.g., their ability level) and the difficulty of the item, or in this case the exposure level of the subject and the severity of the item. In this model every item is considered to be an equally good measure of the latent trait it helps delineate, and only differs in its difficulty or severity (as indicated by its position on the item characteristic curve) [34]. Through the selection of items of different difficulty the test developer adjusts how informative the scale is in discriminating people of different abilities or exposure levels. For example, a scale that consisted of multiple high difficulty items and few moderate or easy items would provide an instrument designed to delineate differences in ability levels between individuals with generally high abilities, but would provide much less information about individuals with lower ability levels. IRT provides the test developer with very specific information on the informativeness of the test across levels of the latent trait.

Rasch analysis also differs significantly from more complex IRT approaches in which a primary task of the test developer is to select a model that fits the data. In Rasch analysis one is presented with a very simple model with very specific properties and the test developer’s task is to select items, which in concert, fit the model [35].

While we are unfamiliar with other examples of using IRT to categorize individuals by their exposure level, Rasch analysis seems ideally suited to this task. In the Rasch model items only differ in their difficulty or severity, and are equally good discriminators. A two-parameter model categorizes each item both by difficulty and discriminative ability. A three-parameter model includes an additional term to account for guessing test items correctly. Guessing is not an issue with exposure data. It also seems possible to select examples of exposure of differing severity to a specific type of abuse that would have the same discriminative ability as indicated by similar logistic regression slopes. Hence, a one-parameter model should suffice as a reasonable approximation. This is ideal, as there are very strong advantages to the one-parameter model, such ease of scoring. Further, fit to a Rasch model implies that the scale provides a ‘fundamental measurement’ in which items and persons are measured on an interval scale with a common unit [36,37]. This is a remarkable property, rarely attained in scales used in psychology and psychiatry, even though we often treat them as interval or ratio measurements [37].

## Materials and Method

### Ethics Statement

This Project was reviewed and approved by the McLean Hospital IRB, Assurance # FWA00002744. During the review of this Project, the IRB specifically considered (i) the risks and anticipated benefits, if any, to subjects; (ii) the selection of subjects; (iii) the procedures for securing and documenting informed consent; (iv) the safety of subjects; and (v) the privacy of subjects and confidentiality of the data. All participants gave written informed consent prior to participation.

### Overview

**Fig 1:**
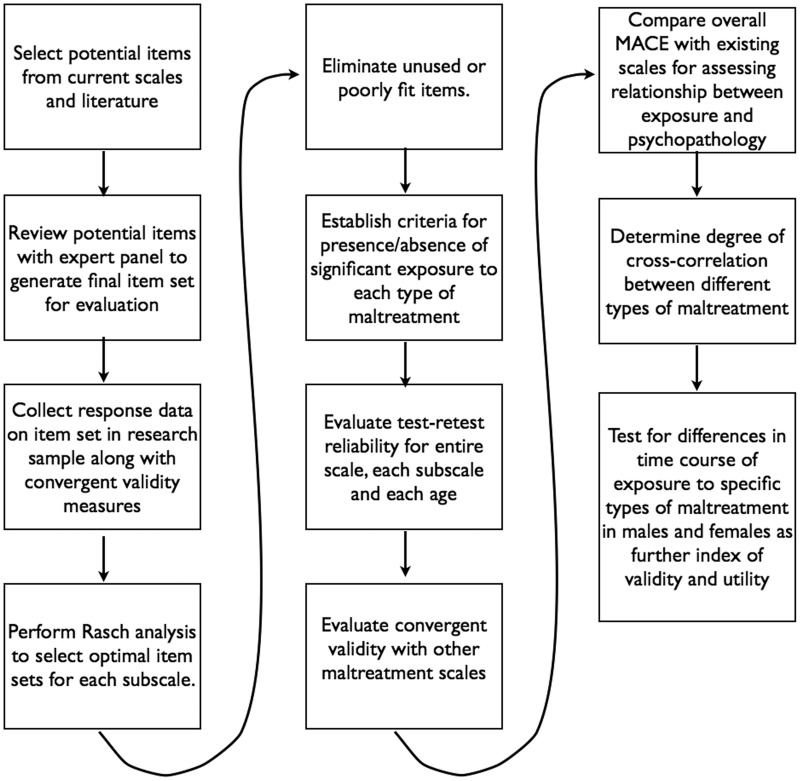
Development and Validation. Flow chart enumerating the sequence of steps used to develop and validate the Maltreatment and Abuse Chronology of Exposure Scale.

The key steps we followed in developing and validating the MACE scale are outlined in Fig. 1. Briefly these steps consisted of item generation, data collection, item selection and elimination using the Rasch model, determination of cutoff scores for clinically-significant exposure levels, assessment of test-retest reliability, assessment of convergent validity, comparison with ACE and CTQ as outcome predictors, analysis of cross-correlations between different types of maltreatment and finally assessment of differences in exposure chronology by maltreatment type and gender.

### Initial Item Selection

Potential items for the MACE were developed following a review of questions contained in the ACE Study Family History Questionnaire, ACE Score Calculator (http://www.acestudy.org/), Childhood Trauma Questionnaire (CTQ) [1,2], Juvenile Victimization Questionnaire [38,39], Childhood Experience of Care and Abuse Interview [40,41,42], Social Experience Questionnaire (SEQ-S) [43,44], Parental and Peer Verbal Aggression Scales [25,45,46] Conflict Tactics Scale [6,47], Abuse and Trauma Questionnaire [25,26,46] and Traumatic Antecedents Interview [48,49]. The goal was to select as possible items examples of maltreatment (specifically forms of abuse, neglect and peer victimization) that individuals directly experienced. Generally, items were reworded so that they could be answered Yes/No, though the items could contain a frequency criteria (e.g., “Said hurtful things that made you feel bad, embarrassed or humiliated more than a few times a year”). This process led to an original pool of 68 items. This item pool was presented to a working group consisting of psychiatrists, psychologists and clinical nurse mental health specialists to identify possible gaps and redundancies. Through a series of iterations the pool was increased to 75 items. The additional items were more specific examples of abuse or neglect enumerated in the original set. On 12 items inquiring about peer physical or verbal abuse there was also a follow-up question to determine if the peer was a ‘date’.

These items were organized into seven sections. Five sections were introduced by referring to specific groups of people (familial, non-familial, peers or a date) doing hurtful things to the respondent, or of whom he was a witness. For instance, one category heading was *“Sometimes parents*, *stepparents or other adults living in the house do hurtful things*.*”* Items in the first five sections were not randomized and resemble an interview situation. Some of the items were worded to be quite specific (e.g., *“Spanked you on your bare (unclothed) buttocks”*), whereas others included a broader spectrum of possible experiences (e.g., *“Intentionally pushed*, *grabbed*, *shoved*, *slapped*, *pinched*, *punched or kicked you*.*”*). The last two sections had no preamble and simply asked subjects to: *“Please indicate if the following happened during your childhood”* or *“Please indicate if the following statements were true about you and your family during your childhood”*.

To differentiate between the occurrence of an experience and the respondents’ subjective perception of the event, we provided check boxes on 35 items whereby the subject could indicate whether they felt helpless or terrified during the experience. Five items included a blank text field for additional information. On one item *“Forced or threatened you to do things that you did not want to do”*, they were asked to provide more information about the acts that they were forced to do. On four positive items they were asked about the person who did this (e.g., *“One or more individuals in your family made you feel loved*.*”* Who?). The scale included eight positive items, which when reverse scored provided information on emotional or physical neglect (e.g., *“One or more individuals in your family helped you feel important or special”*). To finish the questionnaire on a potentially positive note, some of the reverse scored items were presented at the end.

Items included for initial evaluation were selected to potentially assess exposure to the following types of maltreatment: parental verbal abuse; parental non-verbal emotional abuse; parental physical maltreatment; childhood sexual abuse (familial or extra-familial); witnessing interparental violence; witnessing violence to siblings; peer verbal abuse and ostracism; peer physical bullying; emotional neglect and physical neglect. Note, non-verbal emotional abuse and parental verbal abuse were not initially identified as distinct subtypes. Items were included to assess overall degree of exposure to parental emotional abuse. However, subsequent analyses indicated that these items did not fit a single latent construct, but partitioned into two latent constructs best categorized as verbal and non-verbal emotional abuse. Four items were also included to address parental loss, but this category was eliminated from the final instrument, as it did not fit conceptually within an abuse or neglect framework.

Overall, items were selected for testing based on face validity as indexed by their similarity to items included as indices of specific types of abuse, neglect, or peer victimization on prior scales. This approach provided a good framework for selecting items pertaining to physical emotional and sexual abuse, physical and emotional neglect, peer emotional and peer physical abuse, and witnessing interparental violence.

**Criteria for item inclusion in a subscale.** The goal was to develop 10 different short Rasch-type scales to assess exposure to the specific types of maltreatment and neglect mentioned above. These subscales would then be presented as components of a single instrument. A simple Rasch Model was used for item selection because of the ease of calculating scores and benefit of providing a true interval scale. Items were selected to bracket a range of exposure levels, and, when possible, to be maximally informative between the midpoint of the exposure range to the +2 latent exposure level in order to best differentiate individuals with moderate (presumably clinically significant) exposure levels.

The goal of using the Rasch model was to estimate independently the degree of exposure of each subject to a specific type of maltreatment and the severity of each item through a process of unconditional joint maximum-likelihood estimation. The relationship between the severity of each item and the probability that the item will be endorsed by individuals at different exposure levels is illustrated by Item Characteristic Curves (ICC) [37,50]. The informativeness of an item is equal to the reciprocal of the variance, and Item Information Curves illustrates how informative each item is in delineating a range of exposure levels [37,50]. Finally, the Test Information Function combines all of the individual item information curves to indicate how informative the collection of item constituting the subscale are in identifying subjects with different exposure levels.

Rasch modeling software routines in R (ltm, eRm libraries [50]) were used for calculations and plots. The procedure used for item selection was based on Cole et al’s [31] development of a Rasch-based short form of the Center for Epidemiologic Studies—Depression scale (CES-D).

The appropriateness of items selected to a Rasch scale is judged primarily by mean square fit criteria. The mean square fit (χ^2^/df) is an index of fit of an item to the Rasch model, determined by averaging the squared residual for each person—item combination (averaging across all participants for a given item) [31]. Both infit and outfit mean square fits were determined. Infit measures unexpected responses to items with a severity level near one’s exposure level, whereas outfit measures unexpected responses to items with a severity level markedly different from one’s exposure level [51]. Low mean square values are associated with non-significant χ ^2^tests, and so fit the model. However, these low values suggest that the observations might be too predictable due to redundancy or model overfit. High mean square values are associated with significant χ ^2^ tests and indicate noise or poor fit. High mean squares are a much greater threat to validity than low mean squares, which are tolerable, so we focused on the elimination of items with high mean squares from the subscale. There is debate regarding acceptable ranges for means square fit statistics. There is good agreement that mean square fit values of 1.5 or greater are unacceptable. Wright and Stone [52] recommended maximum mean square fits of 1.3, and suggested minimum mean square fits of 0.7. As high mean square fits are much more problematic [52] we strove to select and include items with mean square fits less than 1.3. Some items with mean squares below 0.7 were included, if they were high severity events considered important to the exposure category (e.g., “*Hit or harmed you so severely that it needed medical attention”* in the peer physical bullying subscale).

To explain this more intuitively, items will fit a Rasch latent trait for exposure to a specific type of maltreatment if the items align relatively consistently (unidimensionally) across subjects in their frequency of endorsement. For instance, in terms of physical abuse, subjects will generally endorse that they were *“Intentionally pushed*, *shoved*, *punched or kicked”*, more often then that they were *“hit so hard it left marks for more than a few minutes”* or that *“they were hit or harmed so severely as to need medical attention”*. Further, subjects endorsing this highest level of exposure would also endorsed exposure to the less severe items with very high probability. On the other hand, an individual with high exposure to physical abuse may fail to endorse exposure to even the least severe sexual abuse items. Hence, sexual abuse items would not fit within the physical maltreatment latent trait as they would not show a consistent ordering in relation to the physical maltreatment items, and such inconsistently ordered items would receive high outfit or infit mean square fits. Conversely, the high exposure physical maltreatment item may have a low outfit mean square fit as it is almost certain that no one will endorse this item who had no or very low exposure to physical maltreatment. In this sense response to the item is too predictable, but its inclusion does not threaten the validity of the latent trait, and we chose to include it as it made the scale more informative regarding high levels of exposure.

Once items were selected that provided acceptable infit and outfit mean square values, then the overall model fit was determined using Andersen’s Likelihood ratio (LR)-test [53]. This is a ‘global’ test in which all items are investigated simultaneously. It provides a powerful test of violations of sufficiency and monotonicity, and can also detect differential item functioning (bias). Andersen’s LR is based on partitions of the data into two or more groups. For the determination of model fit and differential item functioning we partitioned subjects by age (median split based on number of days from birth to test day). An acceptable fit (non-significant χ ^2^) was taken as an indication of overall model fit and lack of bias. We avoided partitioning subjects into those with low versus high scores because of the large number of subjects who endorsed zero items, as this would bias parameter estimations by clustering all of these subjects into a single partition.

It follows from the mathematics of the dichotomous Rasch model that the number of items positively endorsed provides an adequate and fair representation of the ability level of the subject [35], or more precisely in this case the exposure level of the subject.

### Scoring Algorithms and Cutoffs

Subscales that contained 5 or more items were scored for severity of exposure to the latent category by determining the number of items with positive endorsements and model parameters. These scores typically fell between values of-4 and +4 and represent mean-centered logit scores. They were recalibrated to range from 0–10, so that total exposure severity levels across the 10 subscales could range from 0–100. Subscales with 4 items could not be scored in this manner due to insufficient item parameters. Instead, they were scored 0, 3, 5, 8 and 10 based on a linear interpolation of number of items positively endorsed.

MACE criterion scores for indicating above threshold exposure within each MACE category were established by comparing MACE severity scores to cut scores of other instruments. Criterion scores were designed to be similar to the ACE (comparator) for the six overlapping categories (sexual abuse, physical maltreatment, emotional neglect, physical neglect, emotional abuse, and witnessing interparental violence). The parental and peer verbal abuse category of the MACE was compared to the Verbal Abuse Questionnaire [46], that provides separate measures of maternal and paternal verbal abuse, as well as of female and male peer verbal abuse. Maternal and paternal verbal abuse score were averaged and a cut score (>40) was used for comparison. Female and male peer verbal abuse scores were combined and a maximum (male or female) score of 30 (significant level of verbal aggression) and 40 (substantial level of verbal aggression) were used as comparison cut scores [25].

The Abuse and Trauma Questionnaire that we created and used in previous studies [25,26,46], provided additional data on 766 of the subjects for establishing criterion scores for exposure to parental physical abuse, peer physical bullying, sexual abuse, witnessing interparental violence and witnessing violence to siblings. This instrument assessed exposure to physical abuse by the question: *‘‘Have you ever been physically hurt or attacked by someone such as a parent*, *another family member or friend (for example have you ever been struck*, *kicked*, *bitten*, *pushed or otherwise physically hurt)*?*”* If so, they were asked to provide information on their relationship to this individual, the number of times they were hurt, age of initiation and termination of these episodes, whether the abuse received, or should have received medical attention, and whether the abuse resulted in permanent injuries or scars. Similarly, sexual abuse was assessed by response to the question: *‘‘Have you ever been forced into doing more sexually than you wanted to do or were too young to understand*? *(By ‘‘sexually” we mean being forced against your will into contact with the sexual parts of your body or his/her body)”*. Witnessing violence was assessed using the question, *“Has an adult member of your family ever purposefully attacked another family member (i*.*e*., *struck*, *kicked*, *bitten*, *pushed*, *hit)*?*”* Followup questions identified the individuals involved, number of times observed, ages of initiation and termination and severity.

### Assessment of reliability and validity

Test-retest reliability was assessed for the overall total MACE Scores and for the MACE Scores across age, using regression coefficients and the Bland & Altman [54] method. This technique eschews regression as insufficient for determining reliability of clinical measures as two tests can be highly correlated but provide very different actual readings. Reliability (repeatability) by Bland & Altman requires first that the mean difference between Test 1 and Test 2 be not significantly different than zero and second that 95% of the differences between Test 1 and Test 2 fall within 2 SD of the mean difference score. This is the definition of reliability adopted by the British Standards Institution.

Convergent validity was determined by comparing total MACE scores to ACE and CTQ scores. Our criteria for convergent validity were correlation coefficients in the range of 0. 6–0.8. However, as the MACE included types of maltreatment not assessed by the ACE or CTQ we expected that these instruments would not account for much more than 50% of the variance in MACE scores.

### Assessment of utility as predictor of psychiatric symptom scores

ACE and CTQ scores have proven useful as research tools as high levels of exposure to childhood adversity, as indicated by these scales, is associated with increased risk for psychopathology [5,9,10,11,12,13,55,56,57,58,59,60,61] and with discernible differences in brain structure and function [62,63,64,65,66]. Hence, we also required that MACE scores be at least as useful as ACE and CTQ scores in ‘predicting’ the association between childhood maltreatment and adult psychopathology. For these comparisons we used seven self-report indices of psychopathology provided by four self-report scales.

The Kellner Symptom Questionnaire (SQ) was used to rate psychiatric symptom severity in four domains (depression, anxiety, anger-hostility, somatization). This is a 92-item yes/no questionnaire that provides current ratings during the past week [67]. We have previously found that SQ scores were substantially increased in individuals reporting exposure to physical, sexual and emotional abuse [46] as well as peer verbal abuse [25]. Dissociation was assessed using the Dissociative Experience Scale (DES) [68], which consists of 28 questions and Likert lines assessing the frequency of occurrence of various dissociative experiences in an individual’s daily life. DES scores are also strongly associated with exposure to multiple forms of abuse [46] as well as peer verbal abuse [25]. ‘Limbic irritability’ was assessed using the Limbic System Checklist-33 [69], which we created to evaluate the frequency with which subjects experienced (throughout their life) symptoms often encountered as ictal temporal lobe epilepsy (TLE) phenomena, based on the work of Spiers et al [70]. These items consist of paroxysmal somatic disturbances, brief hallucinatory events, visual phenomena, automatism, and dissociative experiences. LSCL-33 scores are dramatically influenced by abuse history [69], more so than any other variable we have examined [25,26,46,71]. The Adult Suicidal Ideation Questionnaire [72] (ASIQ) was used to assess suicidal ideation during the past month. It consists of 25 items rated on their frequency of occurrence.

For these contrasts we compared overall MACE scores across age to ACE and CTQ as independent variables. MACE provides two types of scores. MACE Multiplicity indicates the number of different types of maltreatment or neglected reported, and ranges from 0–10, in the same manner as ACE scores. MACE Severity, on the other hand, sums the individual severity scores for each type of maltreatment and can range, theoretically, from 0–100. This is very similar to CTQ total scores, which sum rating of severity to the five types of maltreatment assessed by this instrument, and range from 25–125. Hence, we compared MACE Multiplicity to ACE scores and MACE Severity to total CTQ scores. For these contrasts samples were limited to those individuals who completed both the MACE and the comparator. Ordinary least squares were used in the first analysis to calculate *r* between the exposure score and symptom ratings. Williams test [73] was used to indicate whether MACE provided a significantly stronger or weaker correlation than the comparator scale.

Multiple regression analysis with variance decomposition was used to provide a more exact determination of differences in percent variance in the seven self-report ratings explained by MACE versus comparator scale. We also include age, sex, parental education and perceived financial sufficiency to control for the confounding influence of sociodemographic variables. Assessment of the relative importance of regressor variables in linear models is simple in the special case where all regressors are uncorrelated. Each regressor’s contribution then is their univariate *r* ^*2*^, and all univariate *r* ^*2*^ *-*values add up to the full model *r* ^*2*^. This is rarely true with observational data, and certainly not true in the present circumstance. Regressors were correlated, so that it was no longer straightforward to break down model *r* ^*2*^ into shares from the individual regressors [74]. Hence, we used a technique for variance decomposition developed by Lindeman, Merenda, and Gold [75], and recommended by Johnson and Lebreton [76] and Grömping [74] to more accurately gauge the relative importance of MACE versus comparator scale. Briefly this technique decomposes *r* ^*2*^ by calculating the sequential contribution of each regressor (in which the contribution of a regressor depends on the regressors that come before) by averaging over all possible sequential orderings (R package *relaimpo*).

### Time course of exposure to different types of maltreatment

The most novel feature of the MACE, and primary reason for its development, is the potential capacity of the instrument to delineating the developmental time course of exposure to maltreatment. Linear mixed effect models (R package LME4 and LMERConvenienceFunctions) were used to ascertain: (1) whether there were significant differences in severity of recollected exposure to the 10 types of maltreatment across development; (2) to indicate whether levels of exposure were influenced by gender and parental education; and (3) whether males and females showed a significantly different exposure pattern. For these analyses subjects were nested within levels of financial sufficiency.

Additionally, we hypothesized that different types of maltreatment would have relatively unique exposure patterns. To test whether the 10 types of maltreatment differed from each other in recollected time course we used linear mixed effect models in a pair-wise manner to test the null hypothesis that the normalized exposure patterns for two different types of maltreatment would not show a significant type x time course interaction. Results for males and females were analyzed separately. Overall, there were 45 possible 2-way combinations. Hence, Bonferroni correction was used to adjust p-values to hold the experiment-wise error rate to p < 0.05. Degree of difference in time course (z-transformed probability values) were visualized as a dissimilarity matrix and in 2D Euclidean space using multidimensional scaling [77,78].

### Participants

Subjects for this study were recruited by advertisement using the general tag line *“Memories of Childhood” and were part of a larger study assessing the effects of childhood maltreatment on brain development and risk for depression*. Subjects were screened by phone for age, handedness, head trauma medications and general health as the protocol required that subjects be medically healthy, right handed, unmedicated, unexposed to significant head trauma and between 18–25 years of age for the interview and neuroimaging components. Subjects who passed initial phone screening were provided with a URL and password to a HIPAA-compliant online enrollment system, which collected detailed information on their life experiences, medical and psychiatric history, developmental history, demographics and psychiatric symptomatology plus the preliminary MACE scale. Enrollment targets, for this ongoing study, entailed collection of online data on a sufficient number of subjects to recruit 600 subjects, with equal representation of individuals exposed to 0, 1, 2, 3 and four or more types of maltreatment for diagnostic interviews, and to select 260 of these for neuroimaging. Detailed ratings were collected between November 8, 2010-July 3, 2013, and included just over two-thirds of the proposed sample.

**Demographic characteristics**. Data was collected on race, ethnicity, education, parental education, family income, and perceived financial sufficiency during childhood (1 = much less than enough money to meet our needs, 5 = much more than enough money to meet our needs). We included perceived financial sufficiency as an alternative to family income, as participants were often uncertain of their parents’ income during childhood, and family income could mean very different things depending on locale, family size, and parental spending habits. In all cases, perceived financial sufficiency explained a greater share of the variance in symptom ratings than family income.

The MACE items were completed by 1051 healthy, unmedicated, right-handed, young adults (381M/670F) between 18–25 years of age. From this sample 407 (157M/250F) subjects were invited and came to the laboratory for detailed clinical evaluation, and completed the CTQ. Seventy-five subjects completed a second version of the MACE for test-retest reliability measures. Demographics features are summarized in Table 1. Briefly, the online and interviewed samples were predominantly white, middle-class and well-educated.

**Table 1:** Sociodemographic features of the samples.

Measures	Online Sample	Interviewed Sample
Gender	381 males, 670 females	157 males, 250 females
Age (years ± SD)	23.1 ± 1.7	23.3 ± 1.7
Racial Composition		
White	77%	71%
Black	8%	10%
Asian	10%	12%
American Indian	1%	2%
Other	4%	6%
Hispanic Ethnicity	8%	11%
Parental Education (years ± SD)	16 ± 3	16 ± 3
Subject Education (years ± SD)	16 ± 2	16 ± 2
Family Financial Sufficiency		
Much less than enough money	1.52%	2.46%
Less than enough money	13.61%	16.22%
Enough money	49.00%	48.65%
More than enough money	32.35%	29.98%
Much more than enough money	3.52%	2.70%

## Results

### Rasch Analyses and Convergent Validity

**Fig 2:**
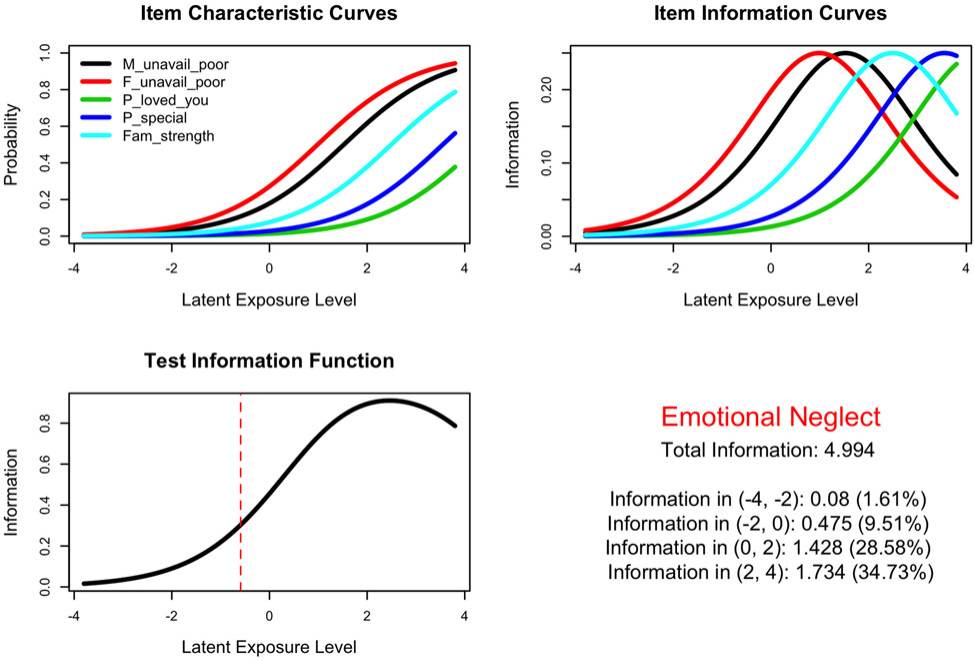
Emotional Neglect. Rasch analysis of emotional neglect subscale showing item characteristic curve, item information curve and test information function.

**Emotional Neglect.** Nine items were considered for inclusion in this scale. Items with excessively high mean square fits were eliminated in turn to produce a five-item scale that fit the model, with three of the items reverse scored (item numbers 42, 43, 52 on the MACE). Final items and fit statistics are provided in Table 2. Eliminated items included unavailability of mother and unavailability of father for enumerated good reasons, plus *“people in your family felt close”* (reverse scored) and *“one or more individuals in your family would help you with your homework*, *or to get ready for school*” (reverse scored). Mean square fit statistics for the final collection were acceptable and ranged from 0.74–1.13. Fig. 2 shows the Item Characteristic Curves (ICC), Item Fit Curves (IFC) and Test Information Function for the emotional neglect Rasch scale. The items covered the latent trait from logit scores of-3.2 (no items selected) to 3.3 (all items selected). The Test Information Function indicated that the scale was most informative between logit scores of 2–4.

**Table 2:** Rasch analysis of emotional neglect scale.

MACE Items	Observations	% Yes	Difficulty	Outfit_MSQ	Infit_MSQ
38 Mother unavailable poor reasons	1045	22.0	-1.12(0.10)	0.84	0.84
39 Father unavailable poor reasons	1045	30.7	-1.79(0.10)	1.13	1.03
42 Family member made you feel loved (reversed)	1045	2.2	1.88(0.19)	0.86	0.75
43 Family member helped you feel special/important (reversed)	1045	4.4	1.08(0.14)	0.79	0.74
52 Family was source of strength and support (reversed)	1045	10.8	-0.05(0.11)	0.78	0.80

The Andersen LR test was non-significant (3.385, df = 4, p = 0.496) indicating acceptable fit. There was good evidence of convergent validity. MACE emotional neglect scores correlated r = 0.583 (95% CI 0.515–0.645, df = 395, p < 10^-16^) with CTQ emotional neglect scores. MACE emotional neglect scores predicted ACE emotional neglect scores with reasonable accuracy (ROC area under the curve = 0.827, 95% CI 0.792–0.862). Subjects indicating emotional neglect on the ACE had mean MACE emotional neglect scores of 3.63 ± 2.20 versus 0.98 ± 1.49 for those indicating no emotional neglect on the ACE (F_1,1039_ = 362.18 p < 10^-16^). A threshold was set at two selected items (logit score—0.59, scaled score 4) to operationally define presence versus absence of emotional neglect on the MACE.

**Non-Verbal Emotional Abuse.** This scale consisted of six items. Mean square fit statistics were acceptable and ranged from 0.72–1.08, Item descriptions and statistics are listed in Table 3. S1 Fig. shows the ICC, IFC and Test Information Function for the Rasch scale. The items covered the latent trait from logit scores of-3.06 (no items selected) to 3.64 (all items selected). The overall Test Information Function indicated that the scale was most informative between logit scores of 0–2.

**Table 3:** Rasch analysis of non-verbal emotional abuse scale.

MACE Items	Observations	% Yes	Difficulty	Outfit_MSQ	Infit_MSQ
5 Locked you in closet, basement, garage, etc.	1048	3.3	2.79(0.17)	0.95	0.72
40 Parent very difficult to please	1048	43.9	-1.17(0.08)	0.99	1.08
41 Parent no time or interest	1048	24.0	0.08(0.08)	0.76	0.85
48 Had to shoulder adult responsibilities	1048	33.9	-0.57(0.08)	0.91	0.93
49 Felt family financial pressure	1048	37.4	-0.79(0.08)	0.95	0.98
50 Kept important secrets/facts from you	1048	30.3	-0.35(0.08)	0.98	0.99

The Andersen LR test was non-significant (0.957, df = 5, p = 0.966) indicating acceptable fit. There was reasonable evidence of convergent validity. MACE non-verbal emotional abuse scores correlated r = 0.553 (95% CI 0.481–0.618, df = 395, p < 10^-16^) with CTQ emotional abuse scores, thought the CTQ emotional abuse scale contained both verbal and non-verbal items.

MACE non-verbal emotional abuse scores predicted ACE emotional abuse scores with reasonable accuracy (ROC area under the curve = 0.811, 95% CI 0.779–0.843). Subjects indicating emotional abuse on the ACE (which also included verbal and non-verbal items) had mean MACE non-verbal emotional abuse scores of 4.86 ± 2.20 versus 2.21 ± 2.09 for those indicating no emotional abuse on the ACE (F_1,1035_ = 230.85 p < 10^-16^). A threshold was set at four selected items (logit score 0.74, scaled score 6) to operationally define presence versus absence of non-verbal emotional abuse on the MACE.

**Parental Physical Maltreatment.** This scale consisted of six of seven considered items, which together provided the best overall fit. Mean square infit and outfit statistics were acceptable as they were all well below 1.3 (Table 4). However, two items had outfit and two infit mean square values < 0.7 indicating some degree of overfit or item redundancy. Being intentionally hit so hard as to require medical attention was the one eliminated item. S2 Fig. shows the ICC, IFC and Test Information Function for the Rasch scale. The items covered the latent trait from logit scores of-4.14 (no items selected) to 4.05 (all items selected). The overall Test Information Function indicated that the scale was most informative between logit scores of 0–2.

**Table 4:** Rasch analysis of parental physical maltreatment scale.

MACE Items	Observations	% Yes	Difficulty	Outfit_MSQ	Infit_MSQ
6. Intentionally pushed, pinched, slapped, kicked etc.	1051	30.9	-0.6 (0.09)	0.91	0.94
7 Hit you so hard it left marks for more than a few minutes	1051	15.1	0.79 (0.1)	0.55	0.7
8 Hit or harmed you so severely that it needed medical attention	1051	3.1	3.14 (0.2)	0.74	0.64
9 Spanked you on buttocks, arms or legs	1051	63.7	-3.27 (0.13)	0.46	0.65
10 Spanked you on unclothed buttocks	1051	23.1	0.02 (0.09)	0.86	1.04
11 Spanked you with object (belt, paddle)	1051	24.5	-0.09 (0.09)	0.70	0.85

The Andersen LR test was non-significant (2.760, df = 5, p = 0.737) indicating acceptable fit. There was good evidence of convergent validity. MACE physical maltreatment scores correlated r = 0.641 (95% CI 0.579–0.695, df = 395, p < 10^-16^) with CTQ physical abuse scores.

MACE parental physical maltreatment scores predicted ACE physical abuse scores with excellent accuracy (ROC area under the curve = 0.938, 95% CI 0.917–0.958). Subjects indicating physical abuse on the ACE had mean MACE physical maltreatment scores of 6.67 ± 1.86 versus 2.36 ± 2.12 for those indicating no physical abuse on the ACE (F_1,1026_ = 408.80, p < 10^-16^). Similarly, mean MACE physical maltreatment scores were 5.89 ± 2.29 vs. 2.27 ± 2.14 in subjects indicating exposure or lack of exposure to parental physical abuse on the ATQ (F_1,756_ = 228.14, p < 10^-16^). A threshold was set at four selected items (logit score 1.06, scaled score 6) to operationally define presence versus absence of physical maltreatment on the MACE.

**Parental Verbal Abuse.** This scale consisted of four of five considered items. *“Yelled or screamed at you more than a few times per year”*was eliminated for excessively high mean square fit statistics. The remaining four items had acceptable mean square infit and outfit statistics that ranged from 0.74–1.04 (Table 5). S3 Fig. shows the ICC, IFC and Test Information Function for the Rasch scale. The overall Test Information Function indicated that the scale was most informative between logit scores of 0–2.

**Table 5:** Rasch analysis of parental verbal abuse scale.

MACE Items	Observations	% Yes	Difficulty	Outfit_MSQ	Infit_MSQ
1 Swore at you, called you names, insulted	1050	30.8	-0.22 (0.08)	0.74	0.79
2 Said hurtful things made you feel humiliated	1050	41.0	-1.19 (0.09)	0.76	0.85
3 Acted in a way that made you feel afraid that you might be physically hurt	1050	30.0	-0.15 (0.08)	0.94	0.97
4 Threatened to leave or abandon you	1050	13.9	1.56 (0.11)	1.04	0.98

The Andersen LR test was non-significant (LR-value: 5.868, df = 3, p = 0.118) indicating acceptable fit. There was good evidence of convergent validity. The four-item MACE parental verbal abuse scores correlated r = 0.687 (95% CI 0.654–0.718, df = 1045, p < 10^-16^) with the 30-item paternal verbal abuse scale (PVAS) score. MACE parental verbal abuse scores predicted significant exposure to verbal abuse on the PVAS with very good accuracy (ROC area under the curve = 0.896, 95% CI 0.872–0.921). Subjects indicating exposure to verbal abuse on the PVAS had mean MACE parental verbal abuse scores of 7.46 ± 2.64 versus 1.99 ± 2.75 for those indicating no verbal abuse on the PVAS (F_1,1045_ = 646.8, p < 10^-16^). A threshold was set at three selected items (logit score 1.32, scaled score 8) to operationally define presence versus absence of parental verbal abuse on the MACE.

Combined MACE parental verbal abuse plus MACE non-verbal emotional abuse correlated r = 0.710 (95% CI 0.657–0.756, df = 395, p < 10^-16^) with CTQ emotional abuse. MACE parental verbal abuse plus non-verbal emotional abuse predicted exposure to emotional abuse on the ACE with ROC area under the curve of 0.910 (95% CI 889–0.930).

**Peer Emotional Abuse.** This scale consisted of all five considered items. Mean square infit and outfit statistics were acceptable (Table 6). However, one item (hurtful) had an outfit mean square fit < 0.7. S4 Fig. shows the ICC, IFC and Test Information Function for the Rasch scale. The items covered the latent trait from logit scores of-2.87 (no items selected) to 3.02 (all items selected). The overall Test Information Function indicated that the scale was most informative between logit scores of 0–2, though also similarly informative between-2–0.

**Table 6:** Rasch analysis of peer emotional abuse.

MACE Items	Observations	% Yes	Difficulty	Outfit_MSQ	Infit_MSQ
26 Swore, called you names/insults more than few times per year	1050	59.3	-0.78 (0.08)	0.71	0.83
27 Said hurtful things made you feel humiliated more than few times per year	1050	65.0	-1.26 (0.09)	0.56	0.73
28 Said things behind you back, spread rumors	1050	48.3	0.04 (0.07)	0.99	1.00
29 Excluded you from activities / groups	1050	46.9	0.14 (0.07)	0.94	0.94
30 Acted in way that made you afraid you might be hurt	1050	21.9	1.86 (0.09)	1.20	0.96

The Andersen LR test was non-significant (5.124, df = 4, p = 0.275) indicating acceptable fit. There was fair evidence of convergent validity. MACE peer emotional abuse scores, which included both verbal abuse and ostracism, correlated r = 0.486 (95% CI 0.438–0.531, df = 1040, p < 10^-16^) with 30-item peer verbal abuse scores (peer-VAS). MACE peer emotional abuse scores predicted significant peer-VAS with good accuracy (ROC area under the curve = 0.803, 95% CI 0.768–0.839). Subjects indicating significant exposure on the peer-VAS had mean MACE peer emotional maltreatment scores of 7.82 ± 2.56 versus 4.26 ± 3.25 for those indicating no significant exposure (F_1,1040_ = 183.5, p < 10^-16^). A threshold was set at four selected items (logit score 1.70, scaled score 8) to operationally define presence versus absence of peer emotional maltreatment on the MACE.

**Peer Physical Bullying.** This scale consisted of all five considered items. Mean square infit and outfit statistics were acceptable (Table 7). However, two severe items (hit and hit requiring medical treatment) had mean square fits < 0.7,suggesting some degree of overfit or redundancy. They were retained as higher severity items important to the latent construct. S5 Fig. shows the ICC, IFC and Test Information Function for the Rasch scale. The items covered the latent trait from logit scores of-3.43 (no items selected) to 3.16 (all items selected). The overall Test Information Function indicated that the scale was most informative between logit scores of 2–4.

**Table 7:** Rasch analysis of peer physical bullying.

**MACE Items**	**Observations**	**% Yes**	**Difficulty**	**Outfit_MSQ**	**Infit_MSQ**
31 Threatened you in order to take money or possessions	1050	6.3	0.89 (0.14)	0.81	0.85
32 Forced you to do things you did not want to	1050	11.0	-0.07 (0.12)	1.00	1.00
33 Intentionally pushed, shoved, punched, kicked you etc.	1050	32.7	-2.45 (0.13)	0.83	0.76
34 Hit you so hard it left marks for more than a few minutes	1050	11.5	-0.15 (0.12)	0.54	0.65
35 Hit or harmed you so severely as to need medical attention	1050	3.3	1.78 (0.17)	0.36	0.68

The Andersen LR test was non-significant (4.298, df = 4, p = 0.367) indicating acceptable fit. MACE Peer Physical Bullying predicted exposure to physical abuse by date, friends, strangers or acquaintances on the ATQ to a significant degree (ROC area under the curve = 0.785, 95% CI = 0.730–0.841). Peer Physical Bullying scores averaged 3.35 ± 2.55 vs. 0.96 ± 1.76 in subjects with or without this type of exposure on the ATQ (F_1,755_ = 107.92, p < 10^-16^). A threshold was set at two selected items (logit score-0.47, scaled score 4) to operationally define presence versus absence of peer physical bullying on the MACE.

**Physical Neglect.** This scale consisted of five out of seven considered items, including 3 that were reversed scored (MACE items 44, 45, 51). Mean square infit and outfit statistics were acceptable though 2 items had low outfit mean squares suggesting some degree of overfit (Table 8). Being left unsupervised and living in foster care were eliminated for excessively high mean square fits. S6 Fig. shows the ICC, IFC and Test Information Function for the Rasch scale. The items covered the latent trait from logit scores of-2.72 (no items selected) to 2.68 (all items selected). The overall Test Information Function indicated that the scale was most informative between logit scores of 2–4.

**Table 8:** Rasch analysis of physical neglect scale.

**MACE Items**	**Observations**	**% Yes**	**Difficulty**	**Outfit_MSQ**	**Infit_MSQ**
44 One or more family members there to take care of you and protect you (reverse)	1050	3.0	0.51 (0.19)	0.63	0.73
45 One or more would be there to take you to doctor or ER if needed (reverse)	1050	2.3	0.92 (0.21)	0.53	0.78
46 You did not have enough to eat	1050	5.7	-0.41 (0.15)	0.91	0.93
47 You had to wear dirty clothes	1050	3.7	0.23 (0.17)	0.99	1.02
51 People in family looked out for each other (reverse)	1050	9.5	-1.25 (0.14)	1.12	1.17

The Andersen LR test for gender was non-significant (4.554, df = 4, p = 0.336) indicating acceptable fit. There was reasonable evidence of convergent validity. MACE physical neglect scores correlated r = 0.579 (95% CI 0.509–0.64, df = 395, p < 10^-16^) with CTQ physical neglect scores. MACE physical neglect scores predicted physical neglect on the ACE with good accuracy (ROC area under the curve = 0.841, 95% CI 0.758–0.924). Subjects indicating significant physical neglect on the ACE had mean MACE physical neglect scores of 4.06 ± 3.27 versus 0.36 ± 1.06 for those indicating no significant neglect (F_1,1039_ = 304.03, p < 10^-16^). A threshold was set at two selected items (logit score-0.45, scaled score 4) to operationally define presence versus absence of physical neglect on the MACE.

**Sexual Abuse.** This scale consisted of seven items, and included adult familial, adult extra-familial and peer sexual abuse. Mean square fit statistics were acceptable and ranged from 0.75–1.12. See Table 9 for description of items and fit statistics. Five additional items were eliminated, as the seven selected provided the best overall model. The eliminated items were similar and included: adult familial attempted or actual intercourse, plus extra-familial adults making sexual comments, having you touch them or attempted intercourse. S7 Fig. shows the ICC, IFC and Test Information Function for the sexual abuse Rasch scale. The items covered the latent trait from logit scores of-3.04 (no items selected) to 2.52 (all items selected). The Test Information Function indicated that the scale was most informative between logit scores of 2–4.

**Table 9:** Rasch analysis of sexual abuse scale.

**MACE Items**	**Observations**	**% Yes**	**Difficulty**	**Outfit_MSQ**	**Infit_MSQ**
12 Parents inappropriate sexual comments to you	967	4.0	0.14 (0.17)	1.12	0.99
13 Parents touched or fondled you in sexual way	967	2.8	0.61 (0.2)	1.08	0.96
14 Parents had you touch them in sexual way	967	1.4	1.39 (0.26)	0.75	0.82
19 Other adults touched or fondled you in sexual way	967	5.6	-0.3 (0.16)	1.12	1.04
20 Other adults had sexual intercourse with you	967	3.7	0.25 (0.18)	0.77	0.84
36 Peer(s) forced you to engage in sexual activity against your will	967	8.2	-0.9 (0.15)	0.95	0.96
37 Peer(s) forced you to do things sexually you did not want to do	967	9.5	-1.18 (0.15)	1.00	0.98

The Andersen LR test was non-significant (3.548, df = 6, p = 0.768) indicating acceptable fit. There was significant evidence of convergent validity. MACE sexual abuse scores correlated r = 0.585 (95% CI 0.516–0.646, df = 395, p < 10^-16^) with CTQ sexual abuse scores. MACE sexual abuse scores predicted ACE sexual abuse scores with reasonable accuracy (ROC area under the curve = 0.832, 95% CI 0.784–0.88). Subjects indicating sexual abuse on the ACE had mean MACE sexual abuse scores of 3.12 ± 2.36 versus 0.38 ± 1.13 for those indicating no sexual abuse on the ACE (F_1,1038_ = 373.8, p < 10^-16^). Similarly, subjects indicating any exposure to sexual abuse on the ATQ had mean MACE sexual abuse scores of 2.55 ± 2.21 vs. 0.14 ± 0.61 for those denying exposure (F_1,757_ = 544, p < 10^-16^). A threshold was set at two selected items (logit score—1.05, scaled score 4) to operationally define presence versus absence of sexual abuse on the MACE.

**Witnessing Interparental Violence.** This scale consisted of five of seven considered items involving witnessing physical abuse to maternal or paternal figures. Mean square infit and outfit statistics were all below 1.3, but three items had outfit mean square values below 0.7 suggesting overfit or item redundancy. See Table 10 for description of items and fit statistics. Hearing and observing adults arguing were eliminated for excessively high mean square values. S8 Fig. shows the ICC, IFC and Test Information Function for the Rasch scale. The items covered the latent trait from logit scores of-3.22 (no items selected) to 3.10 (all items selected). The overall Test Information Function indicated that the scale was most informative between logit scores of 2–4.

**Table 10:** Rasch analysis of witnessing interparental violence.

**MACE Items**	**Observations**	**% Yes**	**Difficulty**	**Outfit_MSQ**	**Infit_MSQ**
21 Saw adults living in household push, slap or throw something at mother (stepmother, grandmother)	1051	12.0	-1.76 (0.17)	0.97	0.94
22 Saw adults hit mother (or surrogates) so hard that it left marks for more than a few minutes	1051	4.7	0.30 (0.18)	0.55	0.66
23 Saw adults hit or harm mother (or surrogates) to the extent that it needed medical attention	1051	2.6	1.26 (0.21)	0.59	0.70
24 Saw adults living in household push, slap or throw something at father (stepfather, grandfather)	1051	9.5	-1.18 (0.16)	1.12	1.10
25 Saw adults hit father (or surrogates) so hard that it left marks for more than a few minutes	1051	2.38%	1.38 (0.217)	0.63	0.94

The Andersen LR test was non-significant (1.360, df = 4, p = 0.851) indicating acceptable fit. There was good evidence of convergent validity. Witnessing interparental violence on the MACE predicted witnessing violence toward mothers on the ACE with good accuracy (ROC area under the curve = 0.862, 95% CI 0.808–0.917). Subjects indicating witnessing violence to mothers on the ACE had mean MACE witnessing interparental violence scores of 4.32 ± 3.05 versus 0.39 ± 1.21 for those that had not (F_1,1036_ = 470.77, p < 10^-16^). Similarly, subjects indicating that they witnessed any degree of interparental violence on the ATQ had mean MACE interparental violence scores of 3.24 ± 2.72 vs. 0.25 ± 1.02 for those who did not (F_1,766_ = 396.53 p < 10^-16^). A threshold was set at two selected items (logit score-0.54, scaled score 4) to operationally define presence versus absence of witnessing interparental violence on the MACE.

**Witnessing Violence to Siblings.** This scale consisted of four of eight considered items involving witnessing physical and/or sexual abuse to siblings. See Table 11 for item description and fit statistics. Eliminated items included observing siblings being hit, and witnessing siblings being made to sexually touch familial adults or attempted or actual intercourse with familial adults. Individually the items selected provided the least satisfactory Rasch statistics as three items had low mean square fits and similar ‘difficulty’ levels consistent with item redundancy However, the Andersen LR test was non-significant (5.034, df = 4, p = 0.169) indicating an acceptable fit for the collection of items. S9 Fig. shows the ICC, IFC and Test Information Function for the Rasch scale. The overall Test Information Function indicated that the scale was most informative between logit scores of 2–4.

**Table 11:** Rasch analysis of witnessing violence to sibling scale.

**MACE Items**	**Observations**	**% Yes**	**Difficulty**	**Outfit_MSQ**	**Infit_MSQ**
15 Parents or adults living in house hit your sibling (stepsibling) so hard that it left marks	839	11.9	-2.4 (0.24)	0.95	0.71
16 Parents… hit or intentionally harmed sibling so that they needed medical attention	839	1.9	0.46 (0.25)	0.42	0.58
17 Parents… made inappropriate sexual comment or suggestions to sibling	839	1.7	0.63 (0.26)	0.63	0.67
18 Parents… touched or fondled sibling in sexual way	839	1.0	1.31 (0.32)	0.38	0.56

Minimal data was collected to support convergent validity for this subscale. There was, as expected, a significant correlation between witnessing interparental violence and witnessing violence to siblings (r = 0.368 (95% CI 0.308–0.425, df = 837, p < 10^-16^), and between experiencing physical abuse and witnessing violence to siblings (r = 0.448 (95% CI 0.392–0.501, df = 837, p < 10^-16^). Although these scales cross-correlated they had very different degrees of association with symptom ratings as previously reported by Teicher and Vitaliano [26] in an independent sample using different metrics. For example, witnessing violence toward siblings on the MACE showed a highly significant association with DES scores (β = 2.02, t = 4.75 p < 10^-5^), whereas witnessing interparental violence did not (β = 0.60, t = 1.44, p = 0.15) and experiencing physical abuse had only a marginal association (β = 0.85, t = 1.83, p < 0.07).

Subjects writing in that they witnessed abuse to a sibling on the ATQ had mean MACE witnessing sibling abuse scores of 1.63 ± 2.24 versus 0.42 ± 1.22 for those who had not (F_1,633_ = 22.87, p < 10^-5^). A threshold was set at one selected item (logit score-1.49, scaled score 3) to operationally define presence versus absence of witnessing violence to siblings on the MACE. Threshold was set at this level as previous research indicated that witnessing abuse to siblings was as common as witnessing abuse to parents [26], and this threshold provided a prevalence rate closest to the prevalence rate for witnessing interparental violence on the MACE.

**Total Scale Scores.** The MACE was developed to provide both a CTQ-like total severity score and an ACE-like multiplicity of exposure score. The MACE Severity score can range from 0–100 and correlated 0.738 (95% CI 0.690–0.780, df = 395, p < 10^-16^) with the CTQ total score (possible range 25–125). The MACE Multiplicity score, which ranged from 0–10 correlated 0.698 (95% CI 0.666–0.728, df = 1043, p < 10^-16^) with the ACE score (range 0–10). As expected, MACE Severity and MACE Multiplicity scores are highly correlate (r = 0.930 (95% CI 0.921–0.937, df = 1049, p < 10^-16^).

### Test-Retest Reliability

Test-retest reliability was collected over a 6-month period in 75 (24M/51F) participants 23.5 ± 1.7 years of age. Most of the data were collected from subjects who had completed the online MACE scale and had come to the office for their initial interview. However, data were also collected on 5 subjects who had returned for annual interviews. Mean time between tests was 66 ± 80 days (range 5–441 days). Total MACE scores were extremely reliable (Severity: r = 0.908 {95% CI 0.857–0.941}; Multiplicity: r = 0.879 {95% CI 0.815–0.922, both p values < 10^-16^). There were no significant effects of time delay between test and retest on retest scores (Severity: main effect of delay F_1,71_ = 1.13 p > 0.29; Multiplicity: F_1,71_ = 0.06, p > 0.81), indicating that time lag was not associated with a consistent increase or decrease in MACE measures. Further, there was no significant effect of time delay on the absolute difference between test and retest on MACE Severity scores (F_1,72_ = 0.14 p > 0.70) or MACE Multiplicity scores (F_1,72_ = 1.11, p = 0.30). This indicates that the subjects did not become significantly more inconsistent in their ratings over time.

**Fig 3:**
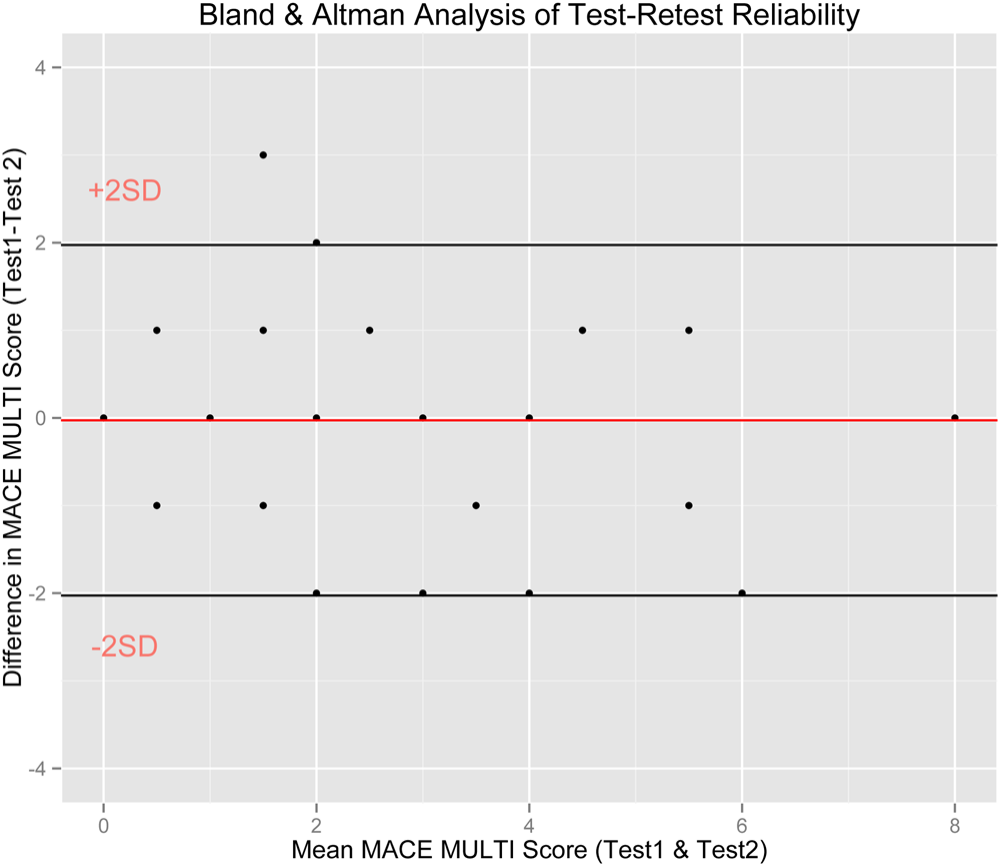
Test-retest Reliability. Bland and Altman analysis of reliability / reproducibility of test—retest scores. Red line indicates the mean difference between test and retest scores, which is close to zero. Horizontal lines indicate confidence intervals showing that test-retest difference scores fall within ± 2 points.

Further, as seen in Fig. 3, total MACE Multiplicity met strict Bland & Altman criteria [79] for test-retest reproducibility, as there was no significant differences in mean scores between test and retest (t_74_ = -0.26, p = 0.80), and 98.7% of differences scores were within ± 2 SD of the mean difference score, meaning that test-retest MACE Multiplicity scores would rarely (< 2%) differ by more than ± 2 points. Similarly, MACE overall severity scores and MACE severity scores across all ages assessed in combination did not differ in any meaningful way between test and retest (1.3 points and 0.3 points on 100 point scale, respectively) and individual test-retest values fell predominantly (93% and 95%) within ± 2 SD of the mean difference score.

**Reliability by Type.** Test-retest reliability for overall degree of exposure to individual types of maltreatment ranged from good to very good (defined as 0.5 < r < 0.8) for emotional neglect, physical neglect, witnessing violence to siblings and peer emotional abuse, to excellent (defined as r > 0.8) for parental NVEA, parental physical maltreatment, parental verbal abuse, peer physical bullying, sexual abuse and witnessing interparental violence) (Table 12).

**Table 12:** Test-retest reliability of MACE subscale scores (n = 75).

Type of Maltreatment	Test-Retest r	Confidence Interval
Emotional Neglect	0.625	{0.464–0.746}
Parental Nonverbal Emotional Abuse	0.826	{0.737–0.887}
Parental Physical Maltreatment	0.874	{0.808–0.919}
Parental Verbal Abuse	0.828	{0.740–0.888}
Peer Emotional Abuse	0.749	{0.629–0.834}
Peer Physical Bullying	0.834	{0.749–0.892}
Physical Neglect	0.643	{0.487–0.759}
Sexual Abuse	0.902	{0.848–0.937}
Witnessing Interparental Violence	0.819	{0.727–0.882}
Witnessing Violence to Siblings	0.741	{0.606–0.834}

**Reliability across age.** Similarly, test-retest reliability for recollected severity scores at each age had good to very good reliability for ages 1–4 and 12, and excellent reliability for all other ages (Table 13).

**Table 13:** Test-retest reliability of MACE severity scores across age (n = 75).

**Recollected Ages**	**Test-Retest r**	**Confidence Interval**
1	0.606	{0.438–0.733}
2	0.584	{0.410–0.717}
3	0.726	{0.597–0.819}
4	0.752	{0.632–0.837}
5	0.831	{0.744–0.891}
6	0.903	{0.849–0.938}
7	0.886	{0.824–0.927}
8	0.891	{0.832–0.930}
9	0.894	{0.837–0.932}
10	0.876	{0.809–0.920}
11	0.852	{0.774–0.904}
12	0.795	{0.692–0.866}
13	0.856	{0.780–0.907}
14	0.832	{0.745–0.891}
15	0.871	{0.802–0.917}
16	0.895	{0.838–0.933}
17	0.863	{0.790–0.911}
18	0.864	{0.792–0.912}

### Prevalence Rates

**Prevalence by Type.** Within the online test sample the self-reported prevalence of exposure ranged from 5.8% for physical neglect to 33.2% for peer emotional abuse (Table 14). Other types of maltreatment with low prevalence in the sample included witnessing interparental violence and childhood sexual abuse. Non-verbal emotional abuse, parental verbal abuse, peer physical abuse and emotional neglect had prevalence rates from 15.7%– 20.8%. Strong gender difference were found in prevalence of reported exposure to childhood sexual abuse (female:male odds ratio 2.95 (1.75–5.22, Fisher exact p < 10^-5^) and peer physical bullying (female:male odds ratio 0.33 (0.23–0.48, Fisher exact p < 10^-9^). Females reported slightly higher prevalence of exposure to emotional neglect, non-verbal emotional abuse and parental verbal abuse (Table 14).

**Table 14:** Prevalence of self-reported exposure to different types of maltreatment in the sample and in male and female participants.

**Exposure**	**N**	**Sample**	**Females**	**Males**	**Odds Ratio**	**Fisher p**
Emotional Neglect	1051	19.70%	22.39%	14.96%	1.64 (1.16–2.34)	0.004
Non-Verbal Emotional Abuse	1049	15.73%	17.49%	12.63%	1.45 (1.00–2.23)	0.05
Parental Physical Maltreatment	1051	12.84%	12.54%	13.39%	0.93 (0.63–1.38)	0.70
Parental Verbal Abuse	1050	20.76%	22.72%	17.32%	1.41 (1.01–1.97)	0.04
Peer Emotional Abuse	1050	33.24%	31.64%	36.05%	0.82 (0.62–1.08)	0.16
Peer Physical Bullying	1050	15.62%	10.15%	25.20%	0.33 (0.23–0.48)	10^-9^
Physical Neglect	1051	5.80%	5.97%	5.51%	1.09 (0.62–1.98)	0.89
Sexual Abuse	1051	10.37%	13.43%	4.99%	2.95 (1.75–5.22)	10^-5^
Witnessing Interparental Violence	1051	8.37%	7.91%	9.19%	0.85 (0.53–1.37)	0.49
Witnessing Violence to Siblings*	839	13.23%	13.73%	12.42%	1.12 (0.73–1.75)	0.60

* In subsample of participants with siblings.

**Multiplicity of exposure.** Overall, 41.77% of the sample, including 41.49% of females and 42.26% of males reported no significant exposure to any type of maltreatment on the MACE (Table 15). This is very similar to the percent of subjects in the sample who reported no significant exposure on the ACE (46.4%). The percent of subjects reporting exposure to one type of maltreatment on the MACE was about half as large as the percent reporting no exposure, and the percent reporting exposure to 2 types was about half as large as the percent reporting exposure to just 1 type. Similarly, 20.9% and 11.3% reported exposure to one or two types of childhood adversity on the ACE. There were no clear-cut gender differences in prevalence of exposure to number of different types of maltreatment on the MACE (χ2 = 7.32 df = 10, p > = 0.69).

**Table 15:** Prevalence of self-reported exposure to number of different types of maltreatment in the entire sample and in male and female participants (n = 1051).

**MACE MULTI**	**Sample**	**Females**	**Males**
0	41.77	41.49	42.26
1	21.98	21.49	22.83
2	13.51	12.99	14.44
3	7.90	8.66	6.56
4	5.04	5.22	4.72
5	4.28	4.78	3.41
6	2.57	2.84	2.10
7	1.43	1.49	1.31
8	0.76	0.45	1.31
9	0.48	0.45	0.52
10	0.29	0.15	0.52

### Comparison of MACE, ACE and CTQ Scores as Predictors of Symptom Ratings

For the MACE to have utility as a research tool for psychiatric and developmental psychopathology studies we required that it would, at least on average, account for as much of the variance in psychiatric symptom scores as the ACE and CTQ. Hence, we compared MACE Severity to CTQ and MACE Multiplicity to ACE in the subsamples that had scores on both instruments. Comparisons were made using ordinary least squares, multiple regression with covariates for age, gender, parental education and financial sufficiency, and with variance decomposition analysis to more accurate assess the percent variance accounted for by the different independent variables after correcting for their cross correlations [74,75]. For the multiple regression analyses the dependent variables were scaled to provide standardized beta weights.

**MACE Multiplicity and ACE Scores.** MACE Multiplicity scores showed numerically greater correlations with symptom measures than ACE scores across all symptom domains (Tables 16, 17). For three of these symptom measures (somatization, ‘limbic irritability’ and suicidal ideation) the differences were large enough to be statistically significant. Multiple regression analysis with sociodemographic covariates revealed highly significant effects of MACE Multiplicity scores on all symptom ratings (Tables 16 and 17). ACE scores, on the other hand, only showed significant effects on ratings of depression, anger-hostility and dissociation when included along with the MACE. Variance decomposition indicated that MACE Multiplicity scores accounted for, on average, 7.48 ± 3.53 (mean ± sd) of the variance in symptom ratings, whereas the ACE accounted for 3.60% ± 1.23%, a 2.07±0.55 fold difference across the seven scales.

**Table 16:** Comparative differences between MACE MULTI and ACE scores in the percent variance they can account for in Kellner Symptom Questionnaire scores (n = 1041).

	**Anxiety Symptoms**		**Depression Symptoms**		**Somatization Symptoms**		**Hostility Symptoms**	
***Ordinary Least Squares***								
	r	p-value	r	p-value	r	p-value	r	p-value
MACE MULTI	0.28	<10^-18^	0.30	<10^-21^	0.3	<10^-22^	0.27	<10^-17^
ACE Score	0.23	<10^-12^	0.26	<10^-16^	0.23	<10^-12^	0.25	<10^-14^
*Williams Test (t)*	*1.91*	*0.06*	*1.57*	*0.12*	*3.08*	*0.002*	*1.08*	*0.28*

***Multiple Regression***								
	Beta	p-value	Est.	p-value	Est.	p-value	Est.	p-value
MACE MULTI	0.119	<10^-7^	0.117	<10^-7^	0.143	<10^-10^	0.099	<10^-5^
ACE Score	0.026	0.28	0.052	0.03	-0.005	0.83	0.051	0.04
Gender	-0.300	<10^-5^	-0.159	0.01	-0.344	<10^-7^	-0.141	0.02
Age	-0.022	0.23	-0.039	0.03	-0.023	0.19	-0.046	0.01
Parental Education	0.005	0.64	0.005	0.68	-0.008	0.46	-0.012	0.28
Financial Sufficiency	-0.055	0.19	-0.023	0.58	-0.089	0.03	-0.017	0.68

***Variance Decomposition***				
MACE MULTI	4.76%	5.35%	5.97%	4.24%
ACE Score	2.37%	3.27%	2.15%	2.86%
Gender	2.13%	0.64%	2.74%	0.51%
Age	0.15%	0.46%	0.17%	0.62%
Parental Education	0.17%	0.20%	0.48%	0.52%
Financial Sufficiency	0.68%	0.48%	1.20%	0.46%

**Table 17:** Comparative differences between MACE MULTI and ACE scores in the percent variance they can account for in symptoms of dissociation, limbic irritability and suicidal ideation.

	**Dissociative Experience Scores (n = 809)**		**Limbic System Checklist-33 (n = 1034)**		**Adult Suicidal Ideation Questionnaire (n = 351)**	
***Ordinary Least Squares***						
	r	p-value	r	p-value	r	p-value
MACE MULTI	0.35	<10^-23^	0.44	<10^-48^	0.42	<10^-14^
ACE Score	0.31	<10^-17^	0.33	<10^-25^	0.33	<10^-9^
*Williams Test (t)*	*1.70*	*0.09*	*5.18*	*<10* ^*–6*^	*2.35*	*0.02*

***Multiple Regression***						
	Beta	p-value	Beta	p-value	Beta	p-value
MACE MULTI	0.123	<10^-7^	0.210	<10^-23^	0.172	<10^-6^
ACE Score	0.065	0.01	0.022	0.35	0.029	0.42
Gender	0.137	0.05	0.079	0.18	0.067	0.50
Age	-0.021	0.30	-0.026	0.13	0.003	0.92
Parental Education	-0.028	0.02	-0.011	0.30	0.044	0.02
Financial Sufficiency	0.022	0.63	0.014	0.72	-0.153	0.03

***Variance Decomposition***			
MACE MULTI	7.25%	13.35%	11.41%
ACE Score	4.62%	4.78%	5.16%
Gender	0.40%	0.16%	0.09%
Age	0.15%	0.21%	0.05%
Parental Education	1.35%	0.74%	0.77%
Financial Sufficiency	0.41%	0.52%	2.11%

**MACE Severity and CTQ Scores.** MACE Severity scores showed numerically greater correlations with symptom measures than CTQ scores on six of the symptom domains (Tables 18, 19). For ‘limbic irritability’ the differences were large enough to be statistically significant. Multiple regression analysis with sociodemographic covariates revealed significant effects of MACE Severity scores on all symptom ratings except anger-hostility (p < 0.09) (Tables 18 and 19). CTQ scores, on the other hand, only showed significant effects on ratings of anger-hostility and suicidality when included along with the MACE. Variance decomposition indicated that MACE Severity scores accounted for, on average, 7.64% ± 4.62% (mean ± sd) of the variance in symptom ratings, whereas the CTQ scores accounted for 3.83 ± 0.95%, a 2.00 ± 1.13 fold difference across the seven scales.

**Table 18:** Comparative differences between MACE SUM and CTQ scores in the percent variance they can account for in Kellner Symptom Questionnaire scores (n = 395).

	**Anxiety Symptoms**		**Depression Symptoms**		**Somatization Symptoms**		**Hostility Symptoms**	
***Ordinary Least Squares***								
	r	p-value	r	p-value	r	p-value	r	p-value
MACE SEVERITY	0.31	<10^-8^	0.34	<10^-11^	0.32	<10^-9^	0.29	<10^-7^
CTQ Score	0.26	<10^-6^	0.30	<10^-8^	0.27	<10^-7^	0.30	<10^-8^
*Williams Test (t)*	*1.37*	*0.17*	*1.40*	*0.16*	*1.40*	*0.16*	*0.44*	*0.66*

***Multiple Regression***								
	Beta	p-value	Beta	p-value	Beta	p-value	Beta	p-value
MACE SEVERITY	0.018	0.0003	0.018	0.0002	0.017	0.0003	0.008	0.09
CTQ Score	0.003	0.52	0.007	0.16	0.004	0.44	0.014	0.01
Gender	-0.389	<10^-4^	-0.138	0.15	-0.392	<10^-4^	-0.132	0.18
Age	0.012	0.66	-0.013	0.64	-0.027	0.31	-0.050	0.07
Parental Education	0.024	0.16	0.044	0.01	0.030	0.07	-0.008	0.64
Financial Sufficiency	-0.109	0.10	-0.149	0.02	-0.173	0.01	-0.064	0.34

***Variance Decomposition***				
MACE SEVERITY	5.68%	7.09%	6.01%	3.86%
CTQ Score	2.94%	4.24%	3.27%	4.71%
Gender	3.67%	0.52%	3.78%	0.53%
Age	0.02%	0.10%	0.31%	0.84%
Parental Education	0.29%	0.82%	0.41%	0.63%
Financial Sufficiency	1.40%	2.04%	2.60%	0.99%

**Table 19:** Comparative differences between MACE SUM and CTQ scores in the percent variance they can account for in symptoms of dissociation, limbic irritability and suicidal ideation.

	**Dissociative Experience Scores (n = 306)**		**Limbic System Checklist-33 (n = 390)**		**Adult Suicidal Ideation Questionnaire (n = 342)**	
***Ordinary Least Squares***						
	r	p-value	r	p-value	r	p-value
MACE SUM	0.28	<10^-5^	0.48	<10^-22^	0.40	<10^-13^
CTQ Score	0.21	<10^-3^	0.30	<10^-8^	0.40	<10^-13^
*Williams Test (t)*	1.68	0.09	5.33	<10^-6^	0.17	0.86

***Multiple Regression***						
	Beta	p-value	Beta	p-value	Beta	p-value
MACE SUM	0.016	0.0035	0.037	<10^-14^	0.016	0.003
CTQ Score	-0.001	0.89	-0.007	0.18	0.018	0.002
Gender	0.086	0.45	0.134	0.14	0.077	0.44
Age	-0.010	0.75	-0.012	0.64	-0.004	0.88
Parental Education	-0.035	0.09	-0.003	0.87	0.051	0.005
Financial Sufficiency	-0.005	0.95	-0.015	0.82	-0.135	0.05

***Variance Decomposition***			
MACE SUM	4.66%	17.50%	8.66%
CTQ Score	1.82%	4.17%	8.51%
Gender	0.18%	0.54%	0.08%
Age	0.06%	0.12%	0.05%
Parental Education	1.62%	0.60%	1.05%
Financial Sufficiency	0.52%	0.97%	1.94%

### Cross-Correlations Between Different Types of Maltreatment

Individuals are frequently exposed to multiple types of abuse, and severities of exposure to different types of abuse are often highly correlated. Table 20 indicates degree of inter-correlation in overall severity scores between the 10 maltreatment subscales on the MACE. Average extent of correlation was relatively modest (mean r = 0.320 ± 0.106). The highest correlations occurred between the two types of parental emotional abuse (verbal and non-verbal emotional abuse r = 0.588), between emotional neglect and verbal and non-verbal emotional abuse (r = 0.504, 0.531, respectively), and between parental verbal abuse and parental physical abuse (r = 0.556). Lowest correlations occurred between peer and parent ratings. For comparison, the average degree of cross-correlation on the CTQ was r = 0.442 ± 0.119.

**Table 20:** Cross-correlation in MACE severity scores to the ten types of maltreatment using pair-wise comparisons.

SUBSCALES	NVEA	PA	PVA	Peer_E	Peer_P	PN	SA	WIPV	WsibV*
Emotional Neglect	0.531	0.320	0.504	0.232	0.216	0.463	0.302	0.250	0.284
Nonverbal Emotional Abuse (NVEA)		0.421	0.588	0.369	0.283	0.322	0.337	0.298	0.330
Parental Physical Maltreatment (PA)			0.556	0.249	0.223	0.294	0.231	0.373	0.448
Parental Verbal Abuse (PVA)				0.367	0.283	0.365	0.312	0.421	0.418
Peer Emotional Abuse (Peer_E)					0.418	0.144	0.239	0.146	0.189
Peer Physical Bullying (Peer_P)						0.230	0.328	0.174	0.243
Physical Neglect (PN)							0.291	0.205	0.336
Sexual Abuse (SA)								0.211	0.291
Witnessing Interparental Violence (WIPV)									0.368

*WsibV—Witnessing Violence to Siblings

Individual cell sizes range from 839–1051.

### Recollected Time Course of Exposure

**Fig 4:**
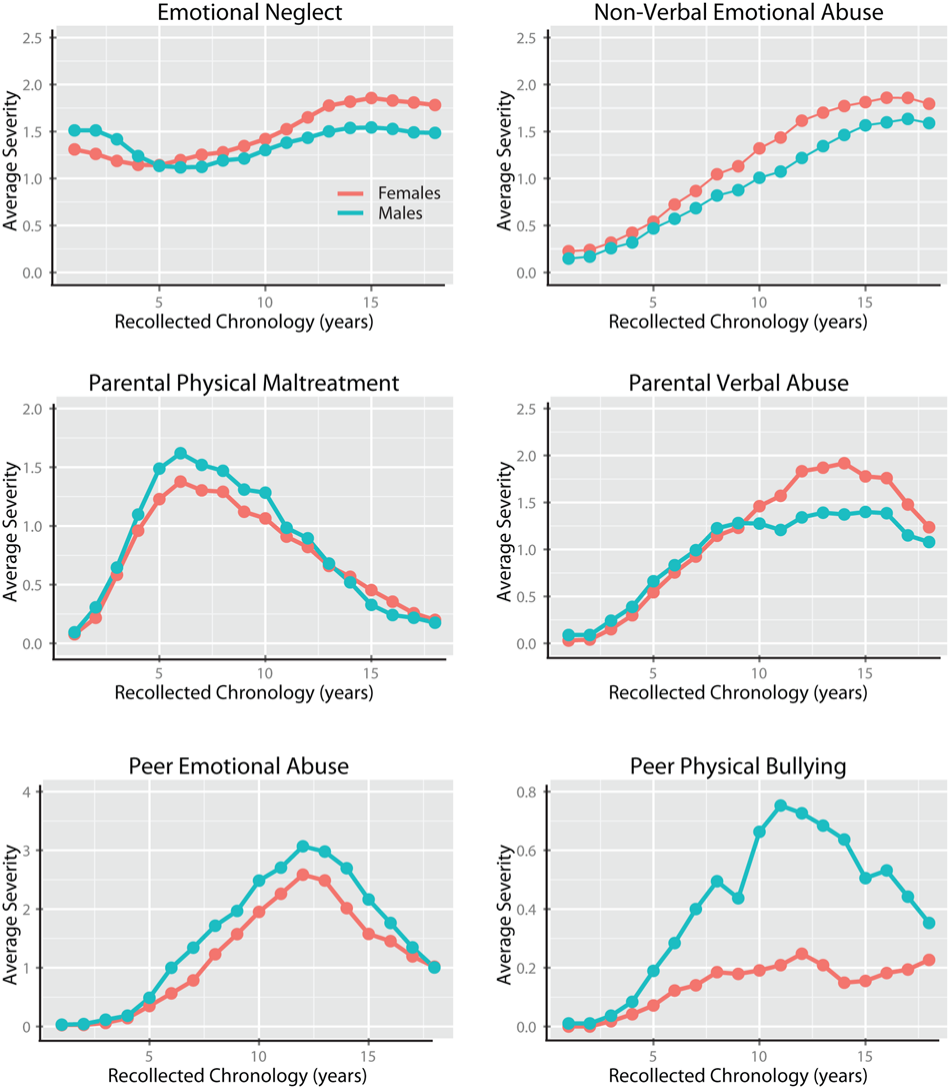
Recollected time course 1. Recollected time course of exposure to emotional neglect, non-verbal emotional abuse, parental verbal abuse, parental physical maltreatment, peer motional abuse and peer physical bullying in males and females.

**Fig 5:**
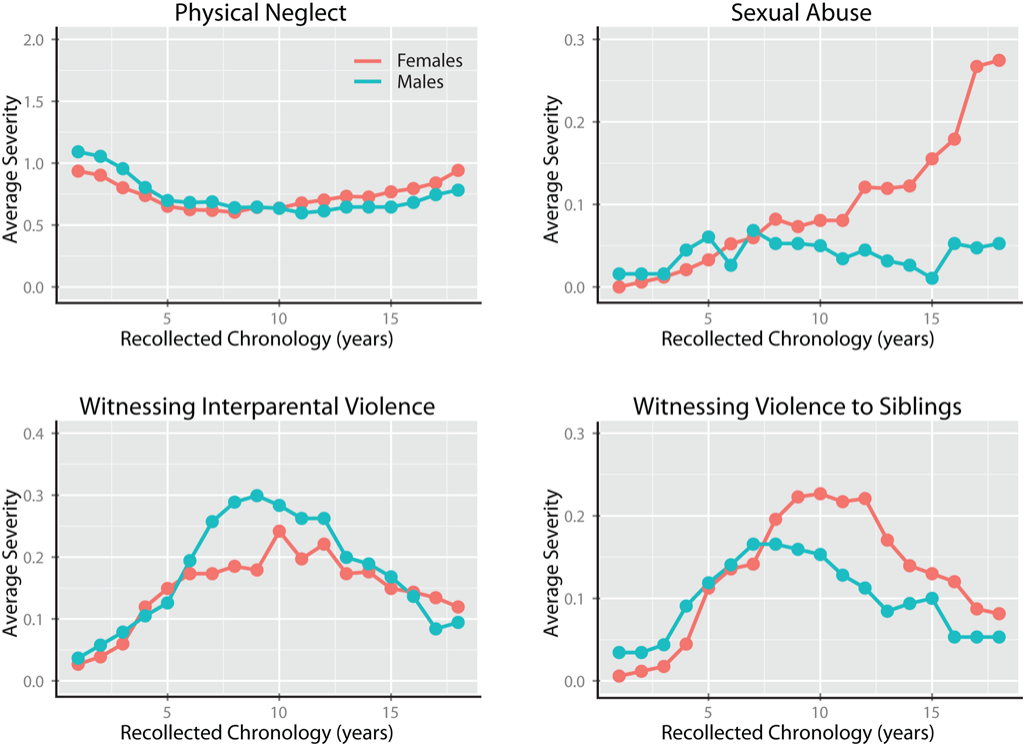
Recollected time course 2. Recollected time course of exposure to physical neglect, familial and non-familial sexual abuse, witnessing interparental violence and witnessing violence to siblings in males and females.

A novel feature of the MACE is the delineation of recollected time course of exposure to each type of maltreatment. Our expectation was that recalled chronology would vary significantly and consistently across years producing a strong main effect of recalled age for most types of maltreatment. However, time course may differ between males and females for certain types of maltreatment. Results of linear mixed effects modeling are summarized in Table 21 and illustrated in Figs. 4 and 5. There were significant main effects of recollected age of exposure on severity scores for all 10 types of maltreatment. However, recollected age explained a substantial (e.g., > 6%) portion of the variance for measures of parental non-verbal emotional abuse, parental physical maltreatment, parental verbal abuse, and peer emotional abuse. Conversely, recollected age had much weaker effects (e.g., ≤ 1% of variance) on ratings of emotional neglect, physical neglect, witnessing interparental violence, and witnessing violence to siblings.

**Table 21:** Linear mixed effects models indicating main effects of recollected ages of exposure, gender and their interaction on severity of exposure scores (n = 1049–1051).

**Types of Exposure**	**Recollected Age**		**Gender**		**Recollected Age x Gender**		**Parental Education**	
	F_17,~18840_	% var.	F_1,~18840_	% var.	F_17,~18840_	% var.	F_1,~18840_	% var.
Emotional Neglect	11.32^f^	0.92%	0.28	0.00%	1.97^b^	0.16%	469.32^f^	2.25%
Parental Nonverbal Emotional Abuse	168.90^f^	11.78%	36.88^e^	0.15%	1.34	0.09%	286.47^f^	1.18%
Parental Physical Maltreatment	89.97^f^	7.16%	29.11^e^	0.14%	1.54	0.12%	272.72^f^	1.28%
Parental Verbal Abuse	75.72^f^	6.01%	3.65	0.02%	3.08^d^	0.24%	215.64^f^	1.01%
Peer Emotional Abuse	174.51^f^	13.44%	99.71^f^	0.45%	2.52^c^	0.19%	0.15	0.00%
Peer Physical Bullying	23.14^f^	1.97%	382.12^f^	1.91%	9.28^f^	0.79%	8.40^b^	0.04%
Physical Neglect	5.79^e^	0.48%	4.62^a^	0.02%	1.05	0.09%	383.22^f^	1.88%
Sexual Abuse	10.60^f^	0.93%	35.77^e^	0.18%	5.21^e^	0.46%	43.33^e^	0.22%
Witnessing Interparental Violence	6.65^e^	0.58%	11.67^c^	0.06%	0.74	0.06%	116.31^f^	0.60%
Witnessing Violence to Siblings*	7.84^f^	0.85%	1.18	0.01%	1.12	0.12%	223.67^f^	1.43%

^a^p<0.05,

^b^p<0.01,

^c^p<.001,

^d^p<10^-4^,

^e^p<10^-6^,

^f^p<10^-16^

* N = 839

There were significant main effects of gender on severity measures for nonverbal emotional abuse, parental physical maltreatment, peer emotional abuse, peer physical bullying, physical neglect, sexual abuse and witnessing interparental violence. There were significant interactive effects between recollected age and gender on five types of maltreatment indicative of gender-specific exposure patterns. Types of maltreatment with significant gender x recalled age interactions were emotional neglect, parental verbal abuse, peer emotional abuse, peer physical bullying and sexual abuse.

Parental education was an important covariate that exerted strong statistical effects on severity scores, particularly for ratings of emotional and physical neglect, but also nonverbal emotional abuse, parental physical abuse, parental verbal abuse and witnessing violence to siblings. However, severity of exposure to peer emotional abuse and physical bullying were minimally affected by parental education levels.

### Recollected Exposure—Differential Time Course

Another key hypothesis is that the different types of maltreatment would each have their own relatively unique time course. Hence, pair-wise comparisons were made between time course for different types of maltreatment to determine whether and how much they differed. Results were analyzed separately for males and females, and statistical significance of multiple cross-comparisons were adjusted using Bonferroni correction.

**Fig 6:**
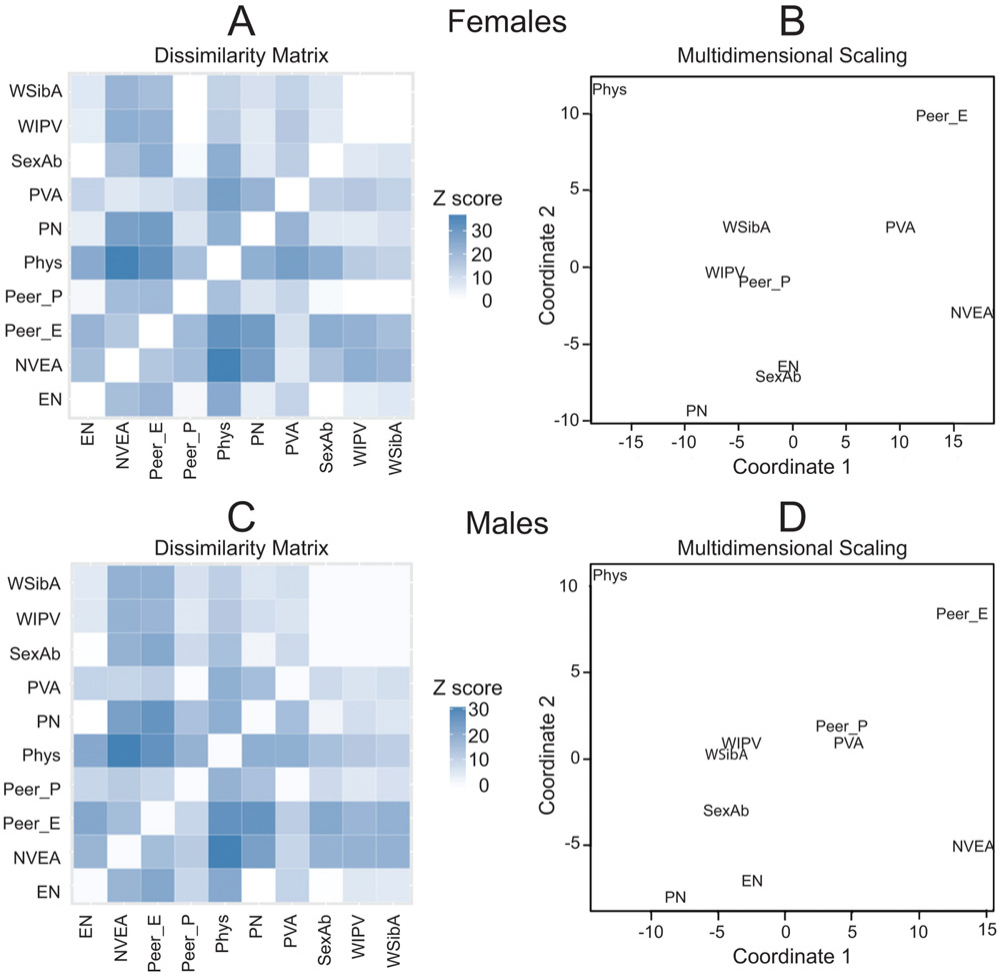
Statistical Differences in Time Course Across Different Types of Abuse. A. Heat map showing Z-score differences in time course of exposure to different types of maltreatment in females. Darker shading is indicative of more significant differences. B. Multidimensional scaling showing in 2-dimensional Euclidean space the degree of similarity or difference in time course of exposure to different types of maltreatment in females. The greater the distance between exposure types the more significant the difference. C. Heat map showing Z-score differences in time course of exposure to different types of maltreatment in males. D. Multidimensional scaling showing in 2-dimensional Euclidean space the degree of similarity or difference in time course of exposure to different types of maltreatment in males. Abbreviations: EN—emotional neglect; NVEA—parental non-verbal abuse; Phys—parental physical maltreament; PVA—parental verbal abuse; Peer_E—peer emotional abuse; Peer_P—peer physical abuse; PN—physical neglect; SexAb—sexual abuse; WIPV—witnessing interparental violence; WSibA—witnessing sibling abuse.

**Females.** All but 4 of the 45-paired comparisons were statistically significant after Bonferroini correction. The four non-significant time course cross comparisons were: Witnessing Interparental Violence / Witnessing Violence to Siblings; Witnessing Interparental Violence / Peer Physical Abuse; Witnessing Violence to Siblings / Peer Physical Abuse and Emotional Neglect / Sexual Abuse. The most significant differences were: Parental Physical Maltreatment / Parental Non-Verbal Emotional Abuse (F_17,24006_ = 91.73, p < 10^-16^); Parental Physical Maltreatment / Peer Emotional Abuse (F_17, 24002_ = 69.05, p < 10^-16^) and Physical Neglect / Peer Emotional Abuse (F_17, 24024_ = 58.07, p < 10^-16^). The significance of the difference in time course between the paired cross comparisons (expressed as z-scores) are highlighted in Fig. 6 as a dissimilarity matrix heat map. Multidimensional scaling provides a 2D summary view in which the degree of statistical difference in time course between the different types of abuse is reflected in the Euclidean distance between the points (Fig. 6).

**Males.** All but 6 of the 45-paired comparisons were statistically significant after Bonferroni correction. The six cross-comparisons that did not differ significantly in time course were: Witnessing Interparental Violence / Witnessing Violence to Siblings; Witnessing Interparental Violence / Sexual Abuse; Witnessing Violence to Siblings / Sexual Abuse, Parental Verbal Abuse / Peer Physical Abuse, Sexual Abuse / Emotional Neglect and Emotional Neglect / Physical Neglect. The most significant differences in time course were between Parental Non-Verbal Emotional Abuse / Parental Physical Maltreatment (F_17, 13656_ = 67.89, p < 10^-16^), Parental Physical Maltreatment / Peer Emotional Abuse (F_17,13656_ = 50.23, p < 10^-16^); and Physical Neglect / Peer Emotional Abuse (F_17,13656_ = 48.31, p < 10^-16^). The significance of the various cross comparisons are illustrated in Fig. 6 as a dissimilarity matrix heat map. Multidimensional scaling provides a 2D summary view in which the degree of statistical difference between types of abuse in time course is reflected in the Euclidean distance between these points.

## Discussion

Research is rapidly progressing on the potential effects of childhood maltreatment on mental and physical well-being, brain development, epigenetic, neuroendocrine and neuroinflammatory processes. Current research has shown overall effects of maltreatment or early life stress and provides evidence for a dose-response relationship between severity or multiplicity of exposure and outcome [80]. Few studies however have delineated sensitive period or examined the specific effects of one type of maltreatment while controlling for degree of exposure to all other types. This is an important area for future research and the MACE was specifically created to facilitate research on the importance of type and timing of exposure, at least to the extent that this can be done through retrospective self-report.

We are well aware that degree of exposure reported during early childhood is only an impression based on what subjects may have been told, what they have inferred from exposure at older ages and perhaps what they witnessed in the way their siblings were treated. Some researchers may prefer to disregard MACE scores below four or five years of age. What we know is that MACE scores at earlier ages are reliable on test-retest, but their potential validity and utility remains to be determined. The items most frequently reported at early ages are spankings, parental unavailability, lack of protection (caring, availability to take to doctor, looking out for each other) and lack of warmth (feeling loved or special or family as a source of strength). With the exception of spanking, (which is known to occur at very high rates during early childhood and then decline [81]), the other events are reported to occur, in most instances, at relatively consistent levels throughout childhood. Hence, it may be a reasonable inference that parents who were emotionally and physically neglectful throughout an individual’s recalled childhood were likely similarly neglectful during their earlier years. Conversely parents who were emotionally and physically caring at recalled ages were probably caring throughout. This however remains to be verified.

Concerns have also expressed about the reliability of retrospective self-report of maltreatment in general for a number of reasons including the presence of memory impairment associated with psychopathology, and the presence of specific mood-congruent memory biases associated with psychopathology [82]. Brewin et al [82], in their comprehensive review, found scant evidence to support these criticisms. What we know in comparing retrospective to prospective reports is that adults tend to minimize their degree of exposure on retrospective report [83,84]. Hence, retrospective exposure rates are lower than prospective rates suggesting a problem with false negative reports but not false positive reports, though this is what critics seem to fear. Individuals reporting abuse retrospectively were those who typically endured the most severe abuse on prospective assessment [83]. This fits with other studies showing that adult reports of abuse are verifiable [85].

Modern instruments for assessing maltreatment including the MACE generally follow Brewin et al [82] recommendation to focus on the occurrence of specific events rather than attitude towards events, and all show impressive test-retest reliability (e.g., CTQ r = 0.88 [86], MACE r = 0.91). On the MACE there is no evidence of negative attribution bias. Ratings of depression and anxiety together account for less than 3% of the variance in retest scores, and the results are in the opposite direction. Increased levels of depression and anxiety were associated with slightly lower retest scores. Indeed, self-reported exposure to maltreatment has been found to be highly consistent over years even in psychotic individuals and not significantly influenced by the severity of their psychosis or their depressive symptoms [87]. The observation that recollected exposure to different types of maltreatment follow their own relatively unique time frames clearly indicates that subjects are nuanced in their response and not simply painting their childhoods as positive or negative based on current mood.

Given this evidence it is not surprising that there is excellent convergence between neurobiological findings in adults reporting exposure retrospectively, in children with contemporaneous exposure [88] and in animal models with experimentally manipulated early life stress [29,89]. Neurobiology is also providing strong convergent evidence from unbiased whole brain analyses which specifically identify alteration in visual cortex [90] and visual-limbic pathway [91] in adults reporting witnessing domestic violence, in auditory cortex [90] and pathways connecting Broca and Wernicke’s area [91] in adults reporting high levels of exposure to parental verbal abuse, and in genital representation area of somatosensory cortex in women reporting childhood sexual abuse [92]. Hence, at least at the group level there is forensic/anatomical evidence supporting the veracity of self-report regarding type of maltreatment experienced.

Neurobiological evidence is also emerging to support veracity of claims regarding self-reported ages of exposure. For example, we reported that visually witnessing domestic violence affects the integrity of the inferior longitudinal fasciculus which interconnects visual cortex and limbic system and determines memory and emotional response to things we see [91]. Diffusion tensor imaging indicates that maltreated and controls differ in degree of myelination and that this is most strongly affected by exposure between 7–13 years [91]. Independent studies indicate that this pathway rapidly myelinates between 7–15 years of age, supporting the apparent sensitivity to exposure within this age range. Similarly, we know that visual cortex is highly plastic in primates until about the time of puberty. Effects of witnessing domestic violence and experiencing sexual abuse on grey matter volume of visual cortex is highly significant prior to [93] or surrounding puberty [90] but not after. In short, retrospective self-report appears to provide potentially useful data for the initial exploration of developmental sensitive periods.

Three versions of this instrument are provided as supplementary material. The first version, referred to as the Maltreatment and Abuse Exposure Scale (MAES) consists of 52 questions that can be used to assess overall degree of exposure and exposure to the 10-types of abuse included in the MACE (S1 File). It however does not inquire about timing of exposure. This instrument is longer than the CTQ (25 items) or ACE score finder (20 questions) but is short enough to be used for the same purposes.

The second version—the MACE—consists of the same 52 questions, plus check boxes to indicate ages of exposure (1–18 years) for each item (S2 File). The third version—the MACE X—is the original developmental tool, which consists of 75 items and also inquires about dating violence and whether specific events led to feelings of helplessness or terror (S3 File). This version is provided for researchers who wish to develop their own version of the instrument, particularly for use in other countries or with other populations (e.g., psychiatric inpatients, prisoners) and who may desire to select items that better discriminate different levels of exposure.

Indeed, colleagues in Germany translated the MACE-X into German and developed a highly comparable instrument that also assesses the same ten types of maltreatment and abuse. Items comprising each maltreatment type are largely but not entirely identical on the German version [94]. To date, the U.S. MACE has been used to delineate sensitive period for effects of maltreatment on amygdala volume in a sample followed longitudinally from infancy to 30 years of age [95].

Included in the supporting information are copies of the MAES (S1 File), MACE (S2 File) and MACE-X (S3 File). Excel spreadsheets for scoring the MAES (S4 File), MACE (S5 File) and MACE-X (S6 File) are also included. In addition, an R package (S7 File, MACEscore) and manual (S8 File) are provided for scoring all three formats, and for checking for, and possibly correcting, common entry errors. All components are provided as open access or with General Public License V2 (MACEscore) to help facilitate their free use as research tools.

## Supporting Information

S1 Fig Parental Non-verbal Emotional Abuse. Rasch analysis of non-verbal emotional abuse subscale showing item characteristic curve, item information curve and test information function.
(TIF)

S2 Fig Parental Physical Maltreatment. Rasch analysis of parental physical maltreatment subscale showing item characteristic curve, item information curve and test information function.
(TIF)

S3 Fig Parental Verbal Abuse. Rasch analysis of parental verbal abuse subscale showing item characteristic curve, item information curve and test information function.
(TIF)

S4 Fig Peer Emotional Abuse. Rasch analysis of peer emotional abuse and ostracism subscale showing item characteristic curve, item information curve and test information function.
(TIF)

S5 Fig Peer Physical Bullying. Rasch analysis of peer physical bullying subscale showing item characteristic curve, item information curve and test information function.
(TIF)

S6 Fig Physical Neglect. Rasch analysis of physical neglect subscale showing item characteristic curve, item information curve and test information function.
(TIF)

S7 Fig Sexual Abuse. Rasch analysis of sexual abuse subscale showing item characteristic curve, item information curve and test information function.
(TIF)

S8 Fig Witnessing Interparental Violence. Rasch analysis of witnessing interparental violence subscale showing item characteristic curve, item information curve and test information function.
(TIF)

S9 Fig Witnessing Violence to Siblings. Rasch analysis of witnessing violence to sibling subscale showing item characteristic curve, item information curve and test information function.
(TIF)

S1 File Formatted copy of the 52-item Maltreatment and Abuse Exposure Scale (MAES) for overall assessment of exposure to ten types of maltreatment across childhood. (DOCX)

S2 File Formatted copy of the 52-item Maltreatment and Abuse Chronology of Exposure Scale (MACE) for assessment of exposure to ten types of maltreatment across each year of childhood. (DOCX)

S3 File Formatted copy of the complete 75-item experimental version of the Maltreatment and Abuse Chronology of Exposure Scale (MACE-X) for assessment of exposure to ten types of maltreatment across each year of childhood. Includes several unscored items. This scale is most useful for researchers endeavoring to develop their own version of the MACE for use with other populations.
(DOCX)

S4 File Excel file for scoring the 52-item MAES. (XLSX)

S5 File Excel file for scoring the 52-item MACE scale for assessing exposure to ten types of maltreatment across each year of childhood. (XLSX)

S6 File Excel file for scoring the experimental 75-item MACE scale for assessing exposure to ten types of maltreatment across each year of childhood. (XLSX)

S7 File R program for scoring MAES, MACE or MACE-X and for checking for, and possibly correcting, common entry errors. (GZ)

S8 File Manual describing functions available in the MACEscore R program. (PDF)

S9 File Data file containing all of the data used to establish Rasch psychometrics for MACE scale. (CSV)

S10 File Data file containing all of the data used to calculate test-retest reliability of MACE scale. (CSV)

S11 File Data file containing data used for analyses in Table 21 and Figs. 4–6. (CSV)
