# Supplementary figures and images for: The ‘Maltreatment and Abuse Chronology of Exposure’ (MACE) Scale for the Retrospective Assessment of Abuse and Neglect During Development
Source: PLoS One. 2015 Feb 25;10(2):e0117423. doi: 10.1371/journal.pone.0117423 (PMC4340880; doi:10.1371/journal.pone.0117423)

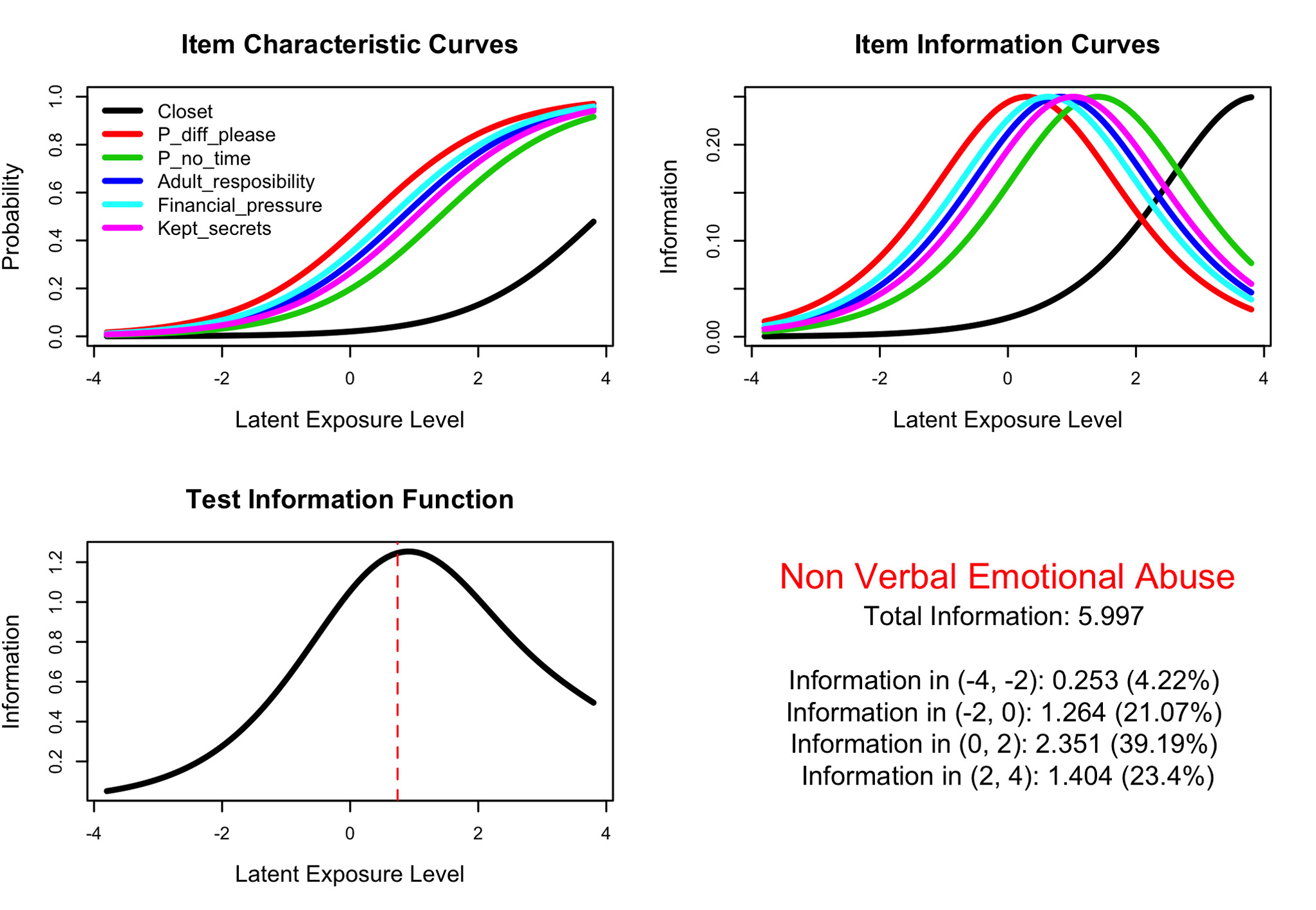

Supplement: S1 Fig — Rasch analysis of non-verbal emotional abuse subscale showing item characteristic curve, item information curve and test information function. (TIF) [file pone.0117423.s001.tif]

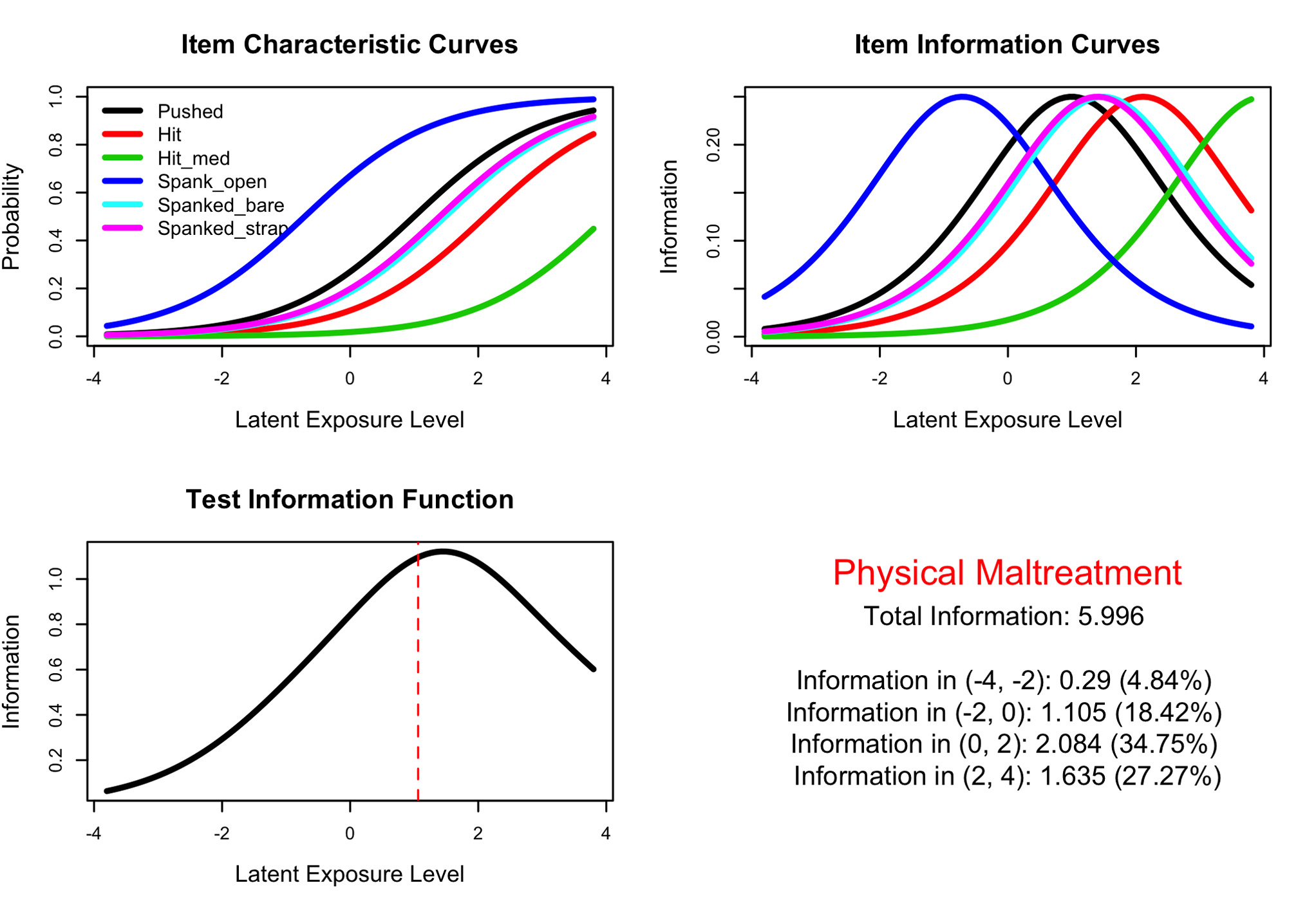

Supplement: S2 Fig — Rasch analysis of parental physical maltreatment subscale showing item characteristic curve, item information curve and test information function. (TIF) [file pone.0117423.s002.tif]

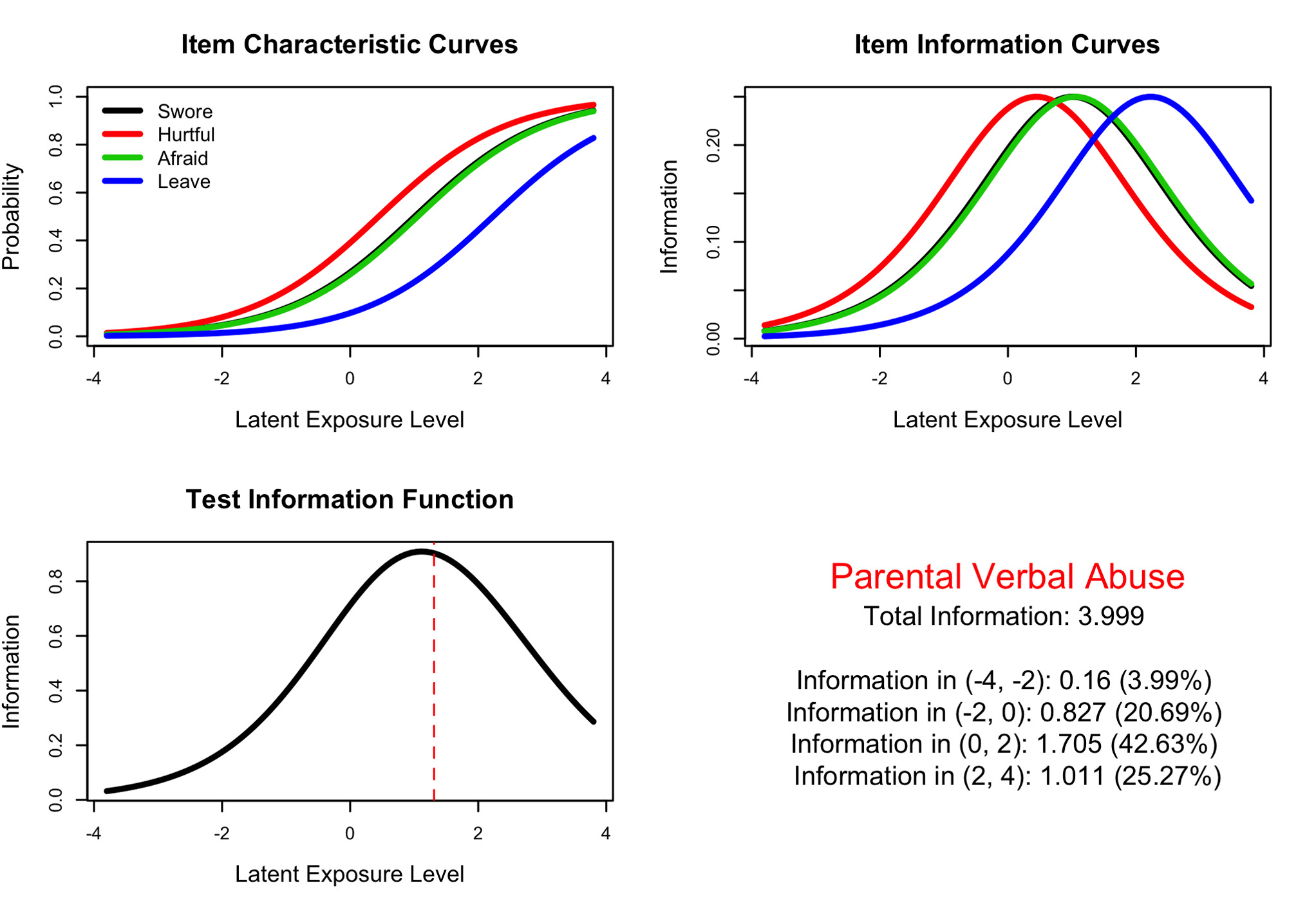

Supplement: S3 Fig — Rasch analysis of parental verbal abuse subscale showing item characteristic curve, item information curve and test information function. (TIF) [file pone.0117423.s003.tif]

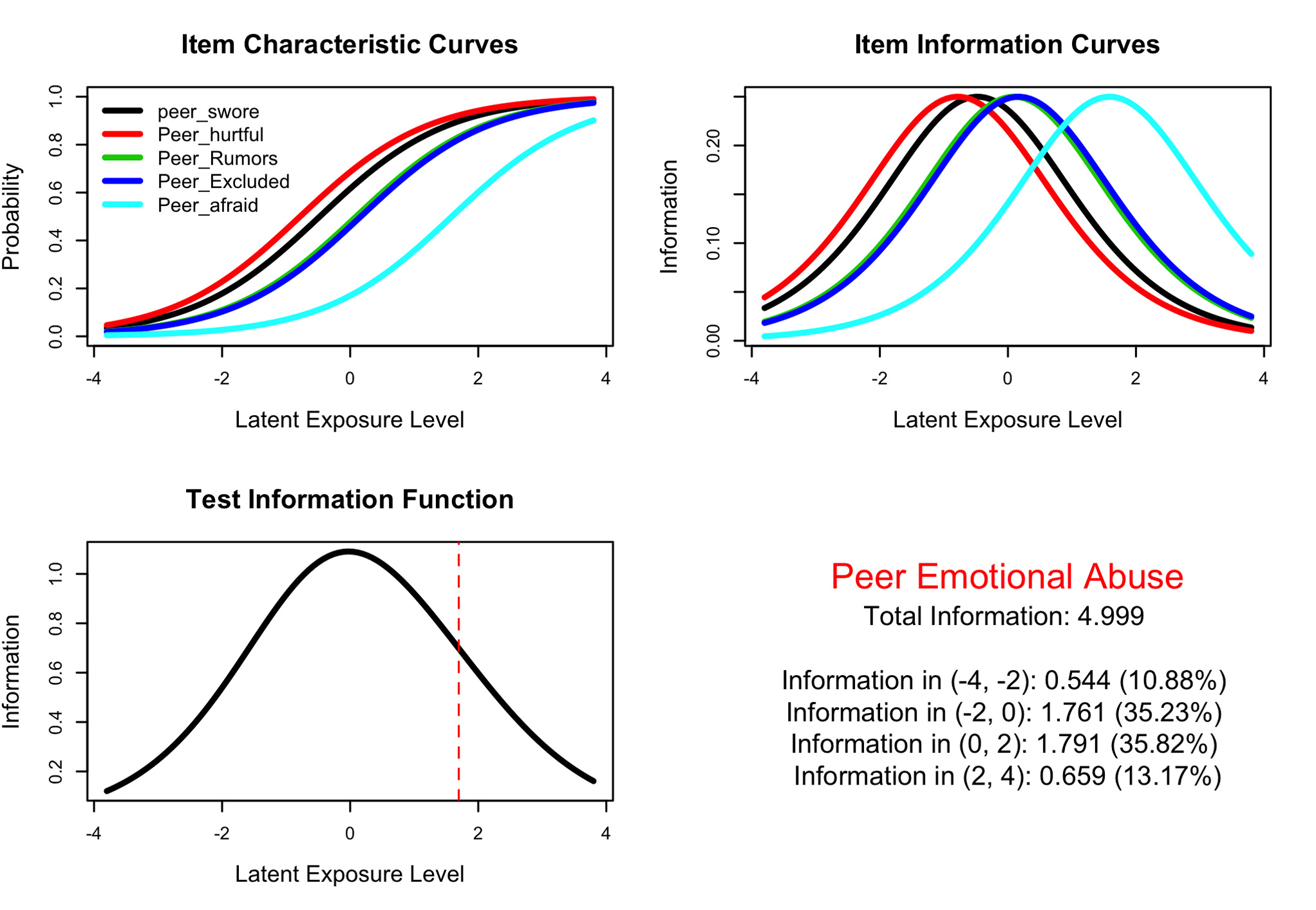

Supplement: S4 Fig — Rasch analysis of peer emotional abuse and ostracism subscale showing item characteristic curve, item information curve and test information function. (TIF) [file pone.0117423.s004.tif]

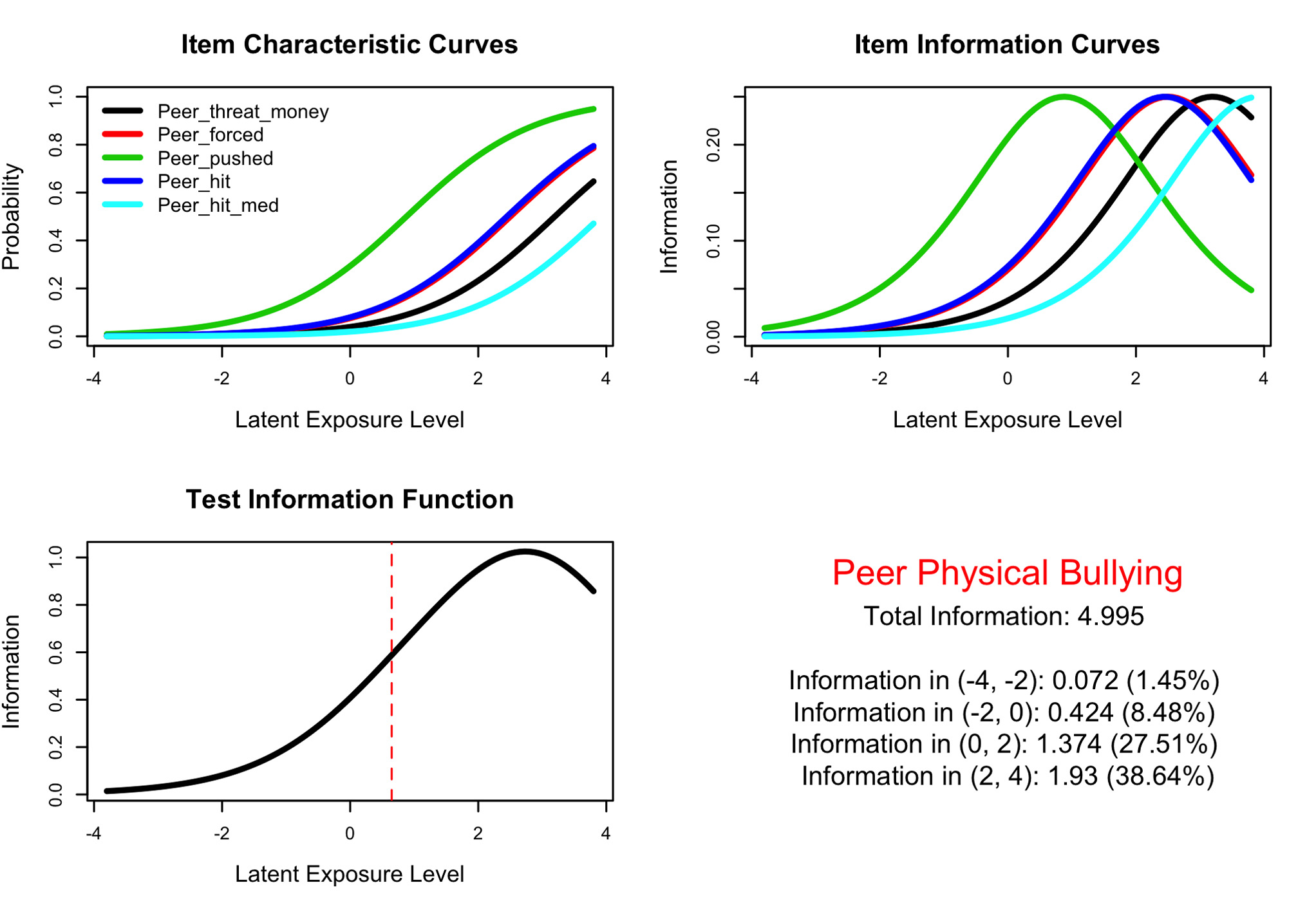

Supplement: S5 Fig — Rasch analysis of peer physical bullying subscale showing item characteristic curve, item information curve and test information function. (TIF) [file pone.0117423.s005.tif]

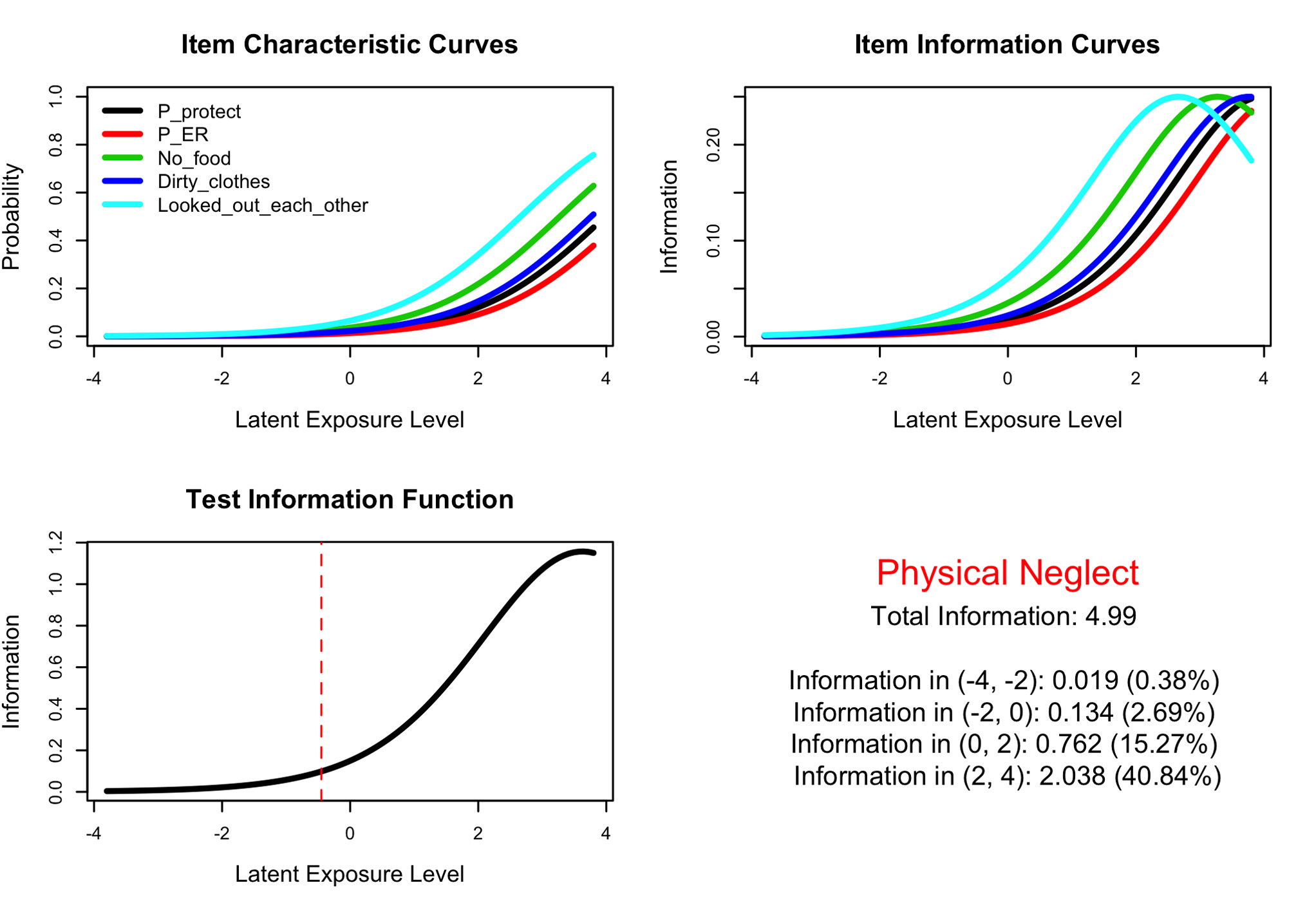

Supplement: S6 Fig — Rasch analysis of physical neglect subscale showing item characteristic curve, item information curve and test information function. (TIF) [file pone.0117423.s006.tif]

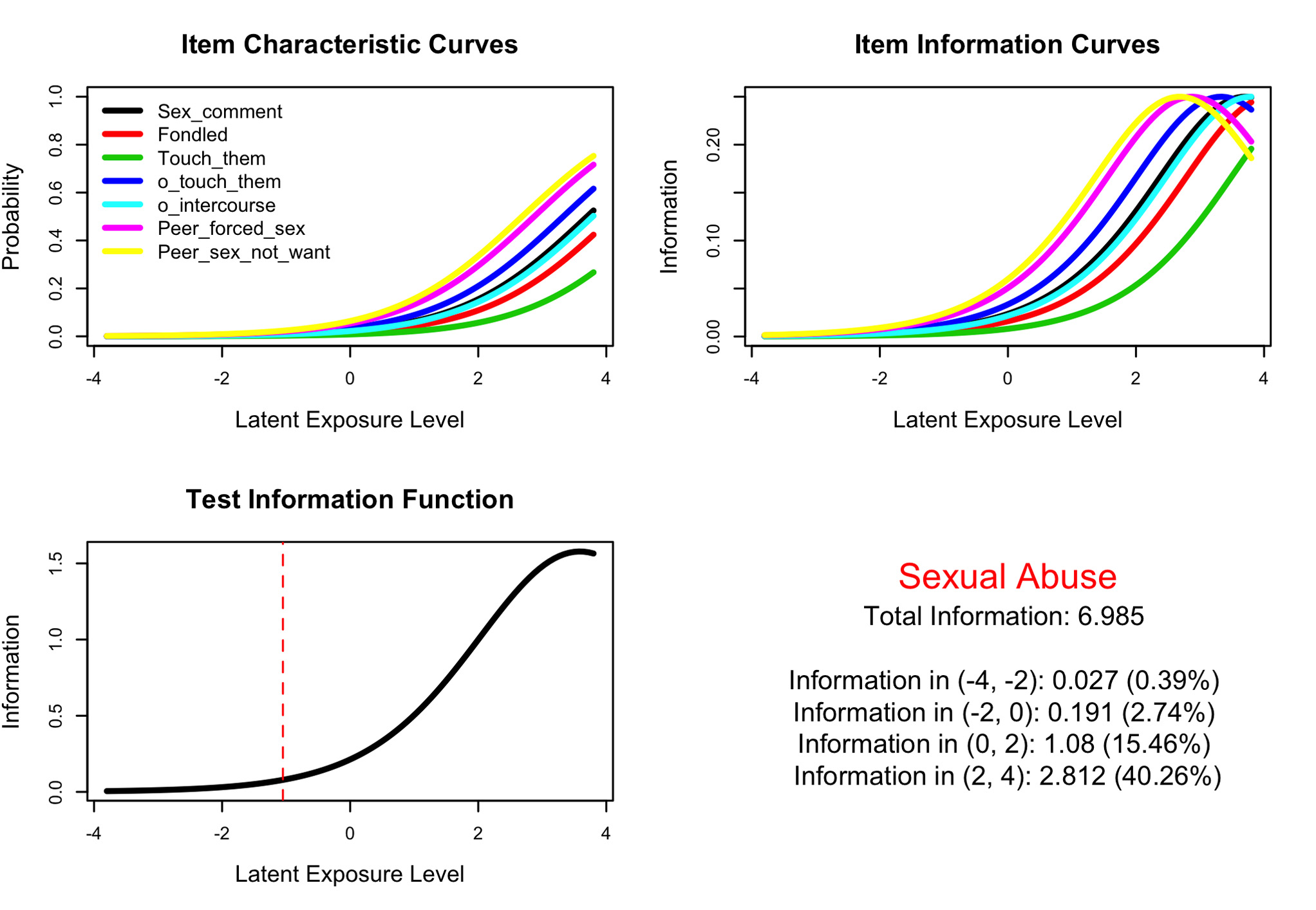

Supplement: S7 Fig — Rasch analysis of sexual abuse subscale showing item characteristic curve, item information curve and test information function. (TIF) [file pone.0117423.s007.tif]

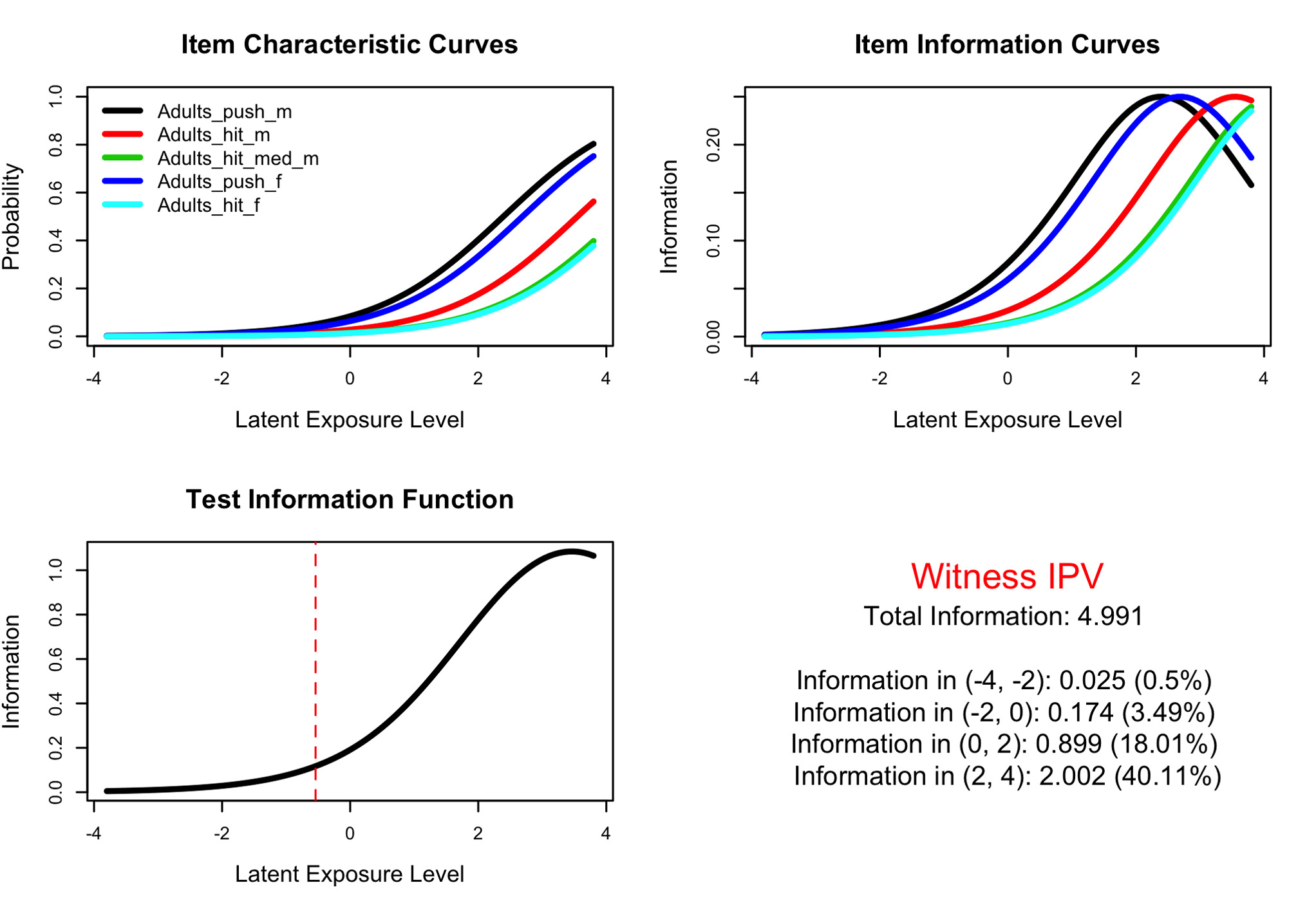

Supplement: S8 Fig — Rasch analysis of witnessing interparental violence subscale showing item characteristic curve, item information curve and test information function. (TIF) [file pone.0117423.s008.tif]

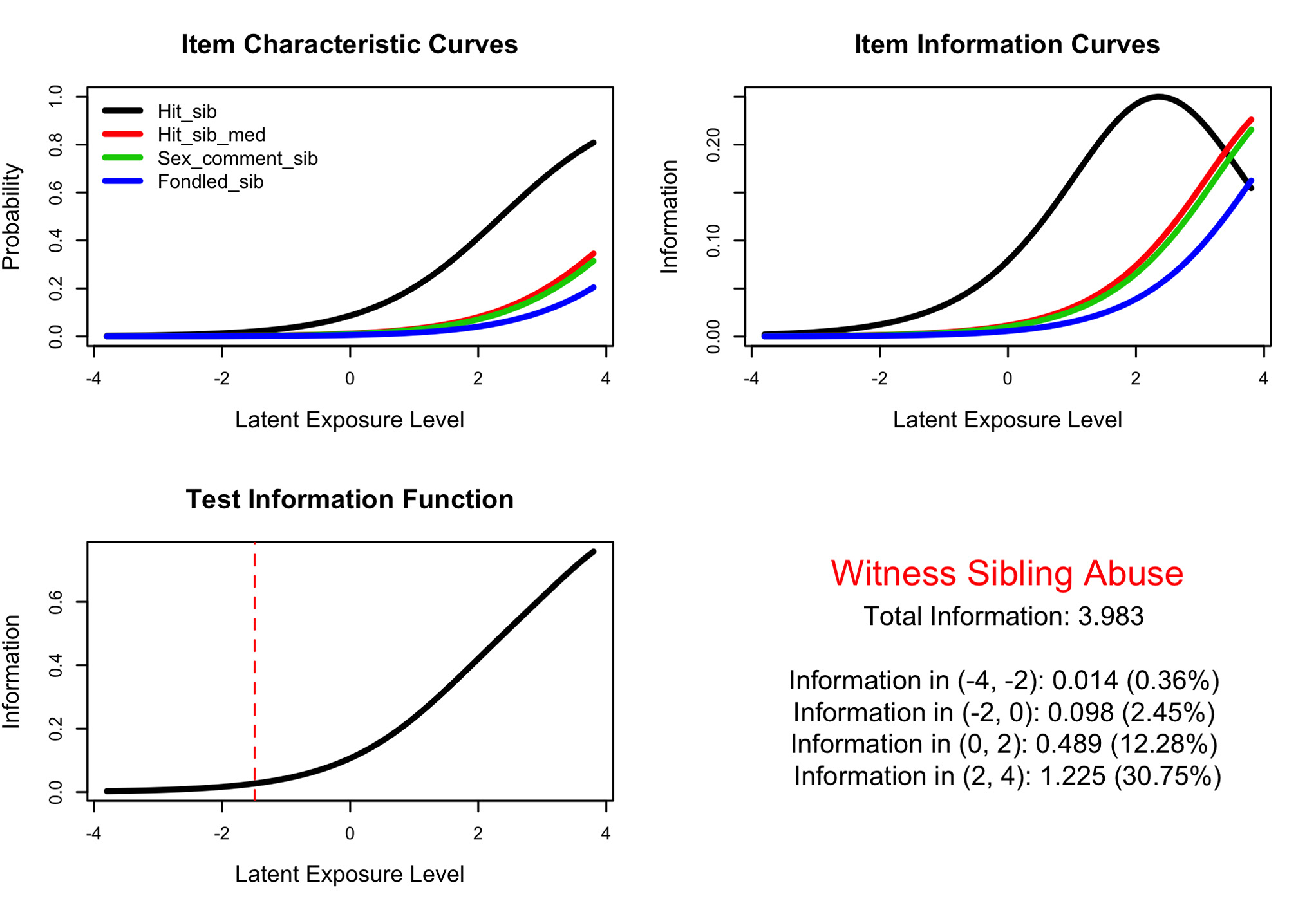

Supplement: S9 Fig — Rasch analysis of witnessing violence to sibling subscale showing item characteristic curve, item information curve and test information function. (TIF) [file pone.0117423.s009.tif]
