# Supplementary material for: The ‘Maltreatment and Abuse Chronology of Exposure’ (MACE) Scale for the Retrospective Assessment of Abuse and Neglect During Development
Source: PLoS One. 2015 Feb 25;10(2):e0117423. doi: 10.1371/journal.pone.0117423 (PMC4340880; doi:10.1371/journal.pone.0117423)
Supplement: S1 File — (DOCX) [file pone.0117423.s010.docx]

MAES

|  | | **Sometimes parents, stepparents or other adults living in the house do hurtful things.**  **If this happened during your childhood (first 18 years of your life), please check ‘Yes’. If this did not happen in your childhood, please check ‘No.’** | |  |  | |
| --- | --- | --- | --- | --- | --- | --- |
| 1. | | Swore at you, called you names, said insulting things like your “fat”, “ugly”, “stupid”, etc. more than a few times a year. | 🔿Yes_1_ | 🔿No_0_ |  | |
| 2. | | Said hurtful things that made you feel bad, embarrassed or humiliated more than a few times a year. | 🔿Yes_1_ | 🔿No_0_ |  | |
| 3. | | Acted in a way that made you afraid that you might be physically hurt. | 🔿Yes_1_ | 🔿No_0_ |  | |
| 4. | | Threatened to leave or abandon you. | 🔿Yes | 🔿No_0_ |  | |
| 5. | | Locked you in a closet, attic, basement or garage. | 🔿Yes_1_ | 🔿No_0_ |  | |
| 6. | | Intentionally pushed, grabbed, shoved, slapped, pinched, punched or kicked you. | 🔿Yes_1_ | 🔿No_0_ |  | |
| 7. | | Hit you so hard that it left marks for more than a few minutes. | 🔿Yes_1_ | 🔿No_0_ |  | |
| 8. | | Hit you so hard, or intentionally harmed you in some way, that you received or should have received medical attention. | 🔿Yes_1_ | 🔿No_0_ |  | |
| 9. | | Spanked you on your buttocks, arms or legs. | 🔿Yes | 🔿No_0_ |  | |
| 10. | | Spanked you on your bare (unclothed) buttocks. | 🔿Yes_1_ | 🔿No_0_ |  | |
| 11. | | Spanked you with an object such as a strap, belt, brush, paddle, rod, etc. | 🔿Yes_1_ | 🔿No_0_ |  | |
| 12. | | Made inappropriate sexual comments or suggestions to you. | 🔿Yes_1_ | 🔿No_0_ |  | |
| 13. | | Touched or fondled your body in a sexual way. | 🔿Yes_1_ | 🔿No_0_ |  | |
| 14. | | Had you touch their body in a sexual way. | 🔿Yes_1_ | 🔿No_0_ |  | |

**Please continue**

**(page 1 of 4)**

|  | **Sometimes parents, stepparents or other adults living in the house do hurtful things to your siblings (brother, sister, stepsiblings).**  **If this happened during your childhood (first 18 years of your life), please check ‘Yes’. If this did not happen in your childhood, please check ‘No.’** | | |  | |  | |
| --- | --- | --- | --- | --- | --- | --- | --- |
| 15. | | Hit your sibling (stepsibling) so hard that it left marks for more than a few minutes. | 🔿Yes_1_ | | 🔿No_0_ | |  |
| 16. | | Hit your sibling (stepsibling) so hard, or intentionally harmed him/her in some way, that he/she received or should have received medical attention. | 🔿Yes_1_ | | 🔿No_0_ | |  |
| 17. | | Made inappropriate sexual comments or suggestions to your sibling (stepsibling). | 🔿Yes_1_ | | 🔿No_0_ | |  |
| 18. | | Touched or fondled your sibling (stepsibling) in a sexual way. | 🔿Yes_1_ | | 🔿No_0_ | |  |

|  | **Sometimes adults or older individuals NOT living in the house do hurtful things to you.**  **If this happened during your childhood (first 18 years of your life), please check ‘Yes’. If this did not happen in your childhood, please check ‘No.’** | | |  | |  | |
| --- | --- | --- | --- | --- | --- | --- | --- |
| 19. | | Had you touch their body in a sexual way. | 🔿Yes_1_ | | 🔿No_0_ | |  |
| 20. | | Actually had sexual intercourse (oral, anal or vaginal) with you. | 🔿Yes_1_ | | 🔿No_0_ | |  |

|  | **Sometimes intense arguments or physical fights occur between parents, stepparents or other adults (boyfriends, girlfriends, grandparents) living in the household.**  **If this happened during your childhood (first 18 years of your life), please check ‘Yes’. If this did not happen in your childhood, please check ‘No.’** | | |  | |  | |
| --- | --- | --- | --- | --- | --- | --- | --- |
| 21. | | Saw adults living in the household push, grab, slap or throw something at your mother (stepmother, grandmother). | 🔿Yes_1_ | | 🔿No_0_ | |  |
| 22. | | Saw adults living in the household hit your mother (stepmother, grandmother) so hard that it left marks for more than a few minutes. | 🔿Yes_1_ | | 🔿No_0_ | |  |
| 23. | | Saw adults living in the household hit your mother (stepmother, grandmother) so hard, or intentionally harm her in some way, that she received or should have received medical attention. | 🔿Yes_1_ | | 🔿No_0_ | |  |
| 24. | | Saw adults living in the household push, grab, slap or throw something at your father (stepfather, grandfather). | 🔿Yes_1_ | | 🔿No_0_ | |  |
| 25. | | Saw adults living in the household hit your father (stepfather, grandfather) so hard that it left marks for more than a few minutes. | 🔿Yes_1_ | | 🔿No_0_ | |  |

|  | **Sometimes children your own age or older do hurtful things like bully or harass you.**  **If this happened during your childhood (first 18 years of your life), please check ‘Yes’. If this did not happen in your childhood, please check ‘No.’** | | |  | |  | |
| --- | --- | --- | --- | --- | --- | --- | --- |
| 26. | | Swore at you, called you names, said insulting things like your “fat”, “ugly”, “stupid”, etc. more than a few times a year. | 🔿Yes_1_ | | 🔿No_0_ | |  |
| 27. | | Said hurtful things that made you feel bad, embarrassed or humiliated more than a few times a year. | 🔿Yes_1_ | | 🔿No_0_ | |  |
| 28. | | Said things behind your back, posted derogatory messages about you, or spread rumors about you. | 🔿Yes_1_ | | 🔿No_0_ | |  |
| 29. | | Intentionally excluded you from activities or groups. | 🔿Yes_1_ | | 🔿No_0_ | |  |
| 30. | | Acted in a way that made you afraid that you might be physically hurt. | 🔿Yes_1_ | | 🔿No_0_ | |  |
| 31. | | Threatened you in order to take your money or possessions. | 🔿Yes_1_ | | 🔿No_0_ | |  |
| 32. | | Forced or threatened you to do things that you did not want to do. | 🔿Yes_1_ | | 🔿No_0_ | |  |
| 33. | | Intentionally pushed, grabbed, shoved, slapped, pinched, punched, or kicked you. | 🔿Yes_1_ | | 🔿No_0_ | |  |
| 33. | | Hit you so hard that it left marks for more than a few minutes. | 🔿Yes_1_ | | 🔿No_0_ | |  |
| 35. | | Hit you so hard, or intentionally harmed you in some way, that you received or should have received medical attention. | 🔿Yes_1_ | | 🔿No_0_ | |  |
| 36. | | Forced you to engage in sexual activity against your will. | 🔿Yes_1_ | | 🔿No_0_ | |  |
| 37. | | Forced you to do things sexually that you did not want to do. | 🔿Yes_1_ | | 🔿No_0_ | |  |

|  | Please indicate if the following happened during your childhood (first 18 years of your life).  If this happened during your childhood (first 18 years of your life), please check ‘Yes’. If this did not happen in your childhood, please check ‘No.’ | | |  | |  | |
| --- | --- | --- | --- | --- | --- | --- | --- |
| 38. | | You felt that your mother or other important maternal figure was present in the household but emotionally unavailable to you for a variety of reasons like drugs, alcohol, workaholic, having an affair, heedlessly pursuing their own goals. | 🔿Yes_1_ | | 🔿No_0_ | |  |
| 39. | | You felt that your father or other important paternal figure was present in the household but emotionally unavailable to you for a variety of reasons like drugs, alcohol, workaholic, having an affair, heedlessly pursuing their own goals. | 🔿Yes_1_ | | 🔿No_0_ | |  |
| 40. | | A parent or other important parental figure was very difficult to please. | 🔿Yes_1_ | | 🔿No_0_ | |  |
| 41. | | A parent or other important parental figure did not have the time or interest to talk to you. | 🔿Yes_1_ | | 🔿No_0_ | |  |
| 42. | | One or more individuals in your family made you feel loved. | 🔿Yes_1_ | | 🔿No_0_ | |  |
| 43. | | One or more individuals in your family helped you feel important or special. | 🔿Yes_1_ | | 🔿No_0_ | |  |
| 44. | | One or more individuals in your family were there to take care of you and protect you. | 🔿Yes_1_ | | 🔿No_0_ | |  |
| 45. | | One or more individuals in your family were there to take you to the doctor or Emergency Room if the need ever arose, or would have if needed. | 🔿Yes_1_ | | 🔿No_0_ | |  |

|  | **Please indicate if the following statements were true about you and your family during your childhood.**  **If this happened during your childhood (first 18 years of your life), please check ‘Yes’. If this did not happen in your childhood, please check ‘No.’** | | |  | |  | |
| --- | --- | --- | --- | --- | --- | --- | --- |
| 46. | | You didn’t have enough to eat. | 🔿Yes_1_ | | 🔿No_0_ | |  |
| 47. | | You had to wear dirty clothes. | 🔿Yes_1_ | | 🔿No_0_ | |  |
| 48. | | You felt that you had to shoulder adult responsibilities. | 🔿Yes_1_ | | 🔿No_0_ | |  |
| 49. | | You felt that your family was under severe financial pressure. | 🔿Yes_1_ | | 🔿No_0_ | |  |
| 50. | | One or more individuals kept important secrets or facts from you. | 🔿Yes_1_ | | 🔿No_0_ | |  |
| 51. | | People in your family looked out for each other. | 🔿Yes_1_ | | 🔿No_0_ | |  |
| 52. | | Your family was a source of strength and support. | 🔿Yes_1_ | | 🔿No_0_ | |  |

Martin H. Teicher & Angelika Parigger
