# Supplementary material for: The ‘Maltreatment and Abuse Chronology of Exposure’ (MACE) Scale for the Retrospective Assessment of Abuse and Neglect During Development
Source: PLoS One. 2015 Feb 25;10(2):e0117423. doi: 10.1371/journal.pone.0117423 (PMC4340880; doi:10.1371/journal.pone.0117423)
Supplement: S2 File — (DOCX) [file pone.0117423.s011.docx]

|  | **Sometimes parents, stepparents or other adults living in the house do hurtful things.**  **If this happened during your childhood (first 18 years of your life) please provide your best estimate of your age at the time(s) of occurrence.**  **Please check all ages that apply.** | |  |  | |  |
| --- | --- | --- | --- | --- | --- | --- |
|  | | *For example item 1.* Swore at you, called you names, said insulting things like your “fat”, “ugly”, “stupid”, etc. more than a few times a year.  *If at ages 6-8 your father swore at you and at ages 8-10 your mother insulted you, and at age 17 your mother’s new live-in boyfriend called you names; you would check off as follows:*   \| 1 \| 2 \| 3 \| 4 \| 5 \| 6 \| 7 \| 8 \| 9 \| 10 \| 11 \| 12 \| 13 \| 14 \| 15 \| 16 \| 17 \| 18 \| \| --- \| --- \| --- \| --- \| --- \| --- \| --- \| --- \| --- \| --- \| --- \| --- \| --- \| --- \| --- \| --- \| --- \| --- \| \|  \|  \|  \|  \|  \| ✓ \| ✓ \| ✓ \| ✓ \| ✓ \|  \|  \|  \|  \|  \|  \| ✓ \|  \| | ⚫  Yes | 🔿  No |  | |
| 1. | | Swore at you, called you names, said insulting things like your “fat”, “ugly”, “stupid”, etc. more than a few times a year.  Please check all ages that apply.   \| 1 \| 2 \| 3 \| 4 \| 5 \| 6 \| 7 \| 8 \| 9 \| 10 \| 11 \| 12 \| 13 \| 14 \| 15 \| 16 \| 17 \| 18 \| \| --- \| --- \| --- \| --- \| --- \| --- \| --- \| --- \| --- \| --- \| --- \| --- \| --- \| --- \| --- \| --- \| --- \| --- \| \|  \|  \|  \|  \|  \|  \|  \|  \|  \|  \|  \|  \|  \|  \|  \|  \|  \|  \| | 🔿  Yes_1_ | 🔿  No_0_ |  | |
| 2. | | Said hurtful things that made you feel bad, embarrassed or humiliated more than a few times a year.  Please check all ages that apply.   \| 1 \| 2 \| 3 \| 4 \| 5 \| 6 \| 7 \| 8 \| 9 \| 10 \| 11 \| 12 \| 13 \| 14 \| 15 \| 16 \| 17 \| 18 \| \| --- \| --- \| --- \| --- \| --- \| --- \| --- \| --- \| --- \| --- \| --- \| --- \| --- \| --- \| --- \| --- \| --- \| --- \| \|  \|  \|  \|  \|  \|  \|  \|  \|  \|  \|  \|  \|  \|  \|  \|  \|  \|  \| | 🔿  Yes_1_ | 🔿  No_0_ |  | |
| 3. | | Acted in a way that made you afraid that you might be physically hurt.  Please check all ages that apply.   \| 1 \| 2 \| 3 \| 4 \| 5 \| 6 \| 7 \| 8 \| 9 \| 10 \| 11 \| 12 \| 13 \| 14 \| 15 \| 16 \| 17 \| 18 \| \| --- \| --- \| --- \| --- \| --- \| --- \| --- \| --- \| --- \| --- \| --- \| --- \| --- \| --- \| --- \| --- \| --- \| --- \| \|  \|  \|  \|  \|  \|  \|  \|  \|  \|  \|  \|  \|  \|  \|  \|  \|  \|  \| | 🔿  Yes_1_ | 🔿  No_0_ |  | |
| 4. | | Threatened to leave or abandon you.  Please check all ages that apply.   \| 1 \| 2 \| 3 \| 4 \| 5 \| 6 \| 7 \| 8 \| 9 \| 10 \| 11 \| 12 \| 13 \| 14 \| 15 \| 16 \| 17 \| 18 \| \| --- \| --- \| --- \| --- \| --- \| --- \| --- \| --- \| --- \| --- \| --- \| --- \| --- \| --- \| --- \| --- \| --- \| --- \| \|  \|  \|  \|  \|  \|  \|  \|  \|  \|  \|  \|  \|  \|  \|  \|  \|  \|  \| | 🔿  Yes | 🔿  No_0_ |  | |
| 5. | | Locked you in a closet, attic, basement or garage.  Please check all ages that apply.   \| 1 \| 2 \| 3 \| 4 \| 5 \| 6 \| 7 \| 8 \| 9 \| 10 \| 11 \| 12 \| 13 \| 14 \| 15 \| 16 \| 17 \| 18 \| \| --- \| --- \| --- \| --- \| --- \| --- \| --- \| --- \| --- \| --- \| --- \| --- \| --- \| --- \| --- \| --- \| --- \| --- \| \|  \|  \|  \|  \|  \|  \|  \|  \|  \|  \|  \|  \|  \|  \|  \|  \|  \|  \| | 🔿  Yes_1_ | 🔿  No_0_ |  | |
| 6. | | Intentionally pushed, grabbed, shoved, slapped, pinched, punched or kicked you.  Please check all ages that apply.   \| 1 \| 2 \| 3 \| 4 \| 5 \| 6 \| 7 \| 8 \| 9 \| 10 \| 11 \| 12 \| 13 \| 14 \| 15 \| 16 \| 17 \| 18 \| \| --- \| --- \| --- \| --- \| --- \| --- \| --- \| --- \| --- \| --- \| --- \| --- \| --- \| --- \| --- \| --- \| --- \| --- \| \|  \|  \|  \|  \|  \|  \|  \|  \|  \|  \|  \|  \|  \|  \|  \|  \|  \|  \| | 🔿  Yes_1_ | 🔿  No_0_ |  | |
| 7. | | Hit you so hard that it left marks for more than a few minutes.  Please check all ages that apply.   \| 1 \| 2 \| 3 \| 4 \| 5 \| 6 \| 7 \| 8 \| 9 \| 10 \| 11 \| 12 \| 13 \| 14 \| 15 \| 16 \| 17 \| 18 \| \| --- \| --- \| --- \| --- \| --- \| --- \| --- \| --- \| --- \| --- \| --- \| --- \| --- \| --- \| --- \| --- \| --- \| --- \| \|  \|  \|  \|  \|  \|  \|  \|  \|  \|  \|  \|  \|  \|  \|  \|  \|  \|  \| | 🔿  Yes_1_ | 🔿  No_0_ |  | |

| 8. | Hit you so hard, or intentionally harmed you in some way, that you received or should have received medical attention.  Please check all ages that apply.   \| 1 \| 2 \| 3 \| 4 \| 5 \| 6 \| 7 \| 8 \| 9 \| 10 \| 11 \| 12 \| 13 \| 14 \| 15 \| 16 \| 17 \| 18 \| \| --- \| --- \| --- \| --- \| --- \| --- \| --- \| --- \| --- \| --- \| --- \| --- \| --- \| --- \| --- \| --- \| --- \| --- \| \|  \|  \|  \|  \|  \|  \|  \|  \|  \|  \|  \|  \|  \|  \|  \|  \|  \|  \| | 🔿  Yes_1_ | 🔿  No_0_ |  |
| --- | --- | --- | --- | --- | --- | --- | --- | --- | --- | --- | --- | --- | --- | --- | --- | --- | --- | --- | --- | --- | --- | --- | --- | --- | --- | --- | --- | --- | --- | --- | --- | --- | --- | --- | --- | --- | --- | --- | --- | --- |
| 9. | Spanked you on your buttocks, arms or legs.  Please check all ages that apply.   \| 1 \| 2 \| 3 \| 4 \| 5 \| 6 \| 7 \| 8 \| 9 \| 10 \| 11 \| 12 \| 13 \| 14 \| 15 \| 16 \| 17 \| 18 \| \| --- \| --- \| --- \| --- \| --- \| --- \| --- \| --- \| --- \| --- \| --- \| --- \| --- \| --- \| --- \| --- \| --- \| --- \| \|  \|  \|  \|  \|  \|  \|  \|  \|  \|  \|  \|  \|  \|  \|  \|  \|  \|  \| | 🔿  Yes_1_ | 🔿  No |  |
| 10. | Spanked you on your bare (unclothed) buttocks.  Please check all ages that apply.   \| 1 \| 2 \| 3 \| 4 \| 5 \| 6 \| 7 \| 8 \| 9 \| 10 \| 11 \| 12 \| 13 \| 14 \| 15 \| 16 \| 17 \| 18 \| \| --- \| --- \| --- \| --- \| --- \| --- \| --- \| --- \| --- \| --- \| --- \| --- \| --- \| --- \| --- \| --- \| --- \| --- \| \|  \|  \|  \|  \|  \|  \|  \|  \|  \|  \|  \|  \|  \|  \|  \|  \|  \|  \| | 🔿  Yes_1_ | 🔿  No_0_ |  |
| 11. | Spanked you with an object such as a strap, belt, brush, paddle, rod, etc.  Please check all ages that apply.   \| 1 \| 2 \| 3 \| 4 \| 5 \| 6 \| 7 \| 8 \| 9 \| 10 \| 11 \| 12 \| 13 \| 14 \| 15 \| 16 \| 17 \| 18 \| \| --- \| --- \| --- \| --- \| --- \| --- \| --- \| --- \| --- \| --- \| --- \| --- \| --- \| --- \| --- \| --- \| --- \| --- \| \|  \|  \|  \|  \|  \|  \|  \|  \|  \|  \|  \|  \|  \|  \|  \|  \|  \|  \| | 🔿  Yes_1_ | 🔿  No_0_ |  |
| 12. | Made inappropriate sexual comments or suggestions to you.  Please check all ages that apply.   \| 1 \| 2 \| 3 \| 4 \| 5 \| 6 \| 7 \| 8 \| 9 \| 10 \| 11 \| 12 \| 13 \| 14 \| 15 \| 16 \| 17 \| 18 \| \| --- \| --- \| --- \| --- \| --- \| --- \| --- \| --- \| --- \| --- \| --- \| --- \| --- \| --- \| --- \| --- \| --- \| --- \| \|  \|  \|  \|  \|  \|  \|  \|  \|  \|  \|  \|  \|  \|  \|  \|  \|  \|  \| | 🔿  Yes_1_ | 🔿  No_0_ |  |
| 13. | Touched or fondled your body in a sexual way.  Please check all ages that apply.   \| 1 \| 2 \| 3 \| 4 \| 5 \| 6 \| 7 \| 8 \| 9 \| 10 \| 11 \| 12 \| 13 \| 14 \| 15 \| 16 \| 17 \| 18 \| \| --- \| --- \| --- \| --- \| --- \| --- \| --- \| --- \| --- \| --- \| --- \| --- \| --- \| --- \| --- \| --- \| --- \| --- \| \|  \|  \|  \|  \|  \|  \|  \|  \|  \|  \|  \|  \|  \|  \|  \|  \|  \|  \| | 🔿  Yes_1_ | 🔿  No_0_ |  |
| 14. | Had you touch their body in a sexual way.  Please check all ages that apply.   \| 1 \| 2 \| 3 \| 4 \| 5 \| 6 \| 7 \| 8 \| 9 \| 10 \| 11 \| 12 \| 13 \| 14 \| 15 \| 16 \| 17 \| 18 \| \| --- \| --- \| --- \| --- \| --- \| --- \| --- \| --- \| --- \| --- \| --- \| --- \| --- \| --- \| --- \| --- \| --- \| --- \| \|  \|  \|  \|  \|  \|  \|  \|  \|  \|  \|  \|  \|  \|  \|  \|  \|  \|  \| | 🔿  Yes_1_ | 🔿  No_0_ |  |

|  | **Sometimes parents, stepparents or other adults living in the house do hurtful things to your siblings (brother, sister, stepsiblings).**  **If this happened during your childhood (first 18 years of your life) please provide your best estimates of your age at the time(s) of occurrence.**  **Please check all ages that apply.** | | |  | |  | |
| --- | --- | --- | --- | --- | --- | --- | --- |
| 15. | | Hit your sibling (stepsibling) so hard that it left marks for more than a few minutes.  Please check all ages that apply.   \| 1 \| 2 \| 3 \| 4 \| 5 \| 6 \| 7 \| 8 \| 9 \| 10 \| 11 \| 12 \| 13 \| 14 \| 15 \| 16 \| 17 \| 18 \| \| --- \| --- \| --- \| --- \| --- \| --- \| --- \| --- \| --- \| --- \| --- \| --- \| --- \| --- \| --- \| --- \| --- \| --- \| \|  \|  \|  \|  \|  \|  \|  \|  \|  \|  \|  \|  \|  \|  \|  \|  \|  \|  \| | 🔿  Yes_1_ | | 🔿  No_0_ | |  |
| 16. | | Hit your sibling (stepsibling) so hard, or intentionally harmed him/her in some way, that he/she received or should have received medical attention.  Please check all ages that apply.   \| 1 \| 2 \| 3 \| 4 \| 5 \| 6 \| 7 \| 8 \| 9 \| 10 \| 11 \| 12 \| 13 \| 14 \| 15 \| 16 \| 17 \| 18 \| \| --- \| --- \| --- \| --- \| --- \| --- \| --- \| --- \| --- \| --- \| --- \| --- \| --- \| --- \| --- \| --- \| --- \| --- \| \|  \|  \|  \|  \|  \|  \|  \|  \|  \|  \|  \|  \|  \|  \|  \|  \|  \|  \| | 🔿  Yes_._ | | 🔿  No_0_ | |  |

| 17. | Made inappropriate sexual comments or suggestions to your sibling (stepsibling).  Please check all ages that apply.   \| 1 \| 2 \| 3 \| 4 \| 5 \| 6 \| 7 \| 8 \| 9 \| 10 \| 11 \| 12 \| 13 \| 14 \| 15 \| 16 \| 17 \| 18 \| \| --- \| --- \| --- \| --- \| --- \| --- \| --- \| --- \| --- \| --- \| --- \| --- \| --- \| --- \| --- \| --- \| --- \| --- \| \|  \|  \|  \|  \|  \|  \|  \|  \|  \|  \|  \|  \|  \|  \|  \|  \|  \|  \| | 🔿  Yes_1_ | 🔿  No_0_ |  |
| --- | --- | --- | --- | --- | --- | --- | --- | --- | --- | --- | --- | --- | --- | --- | --- | --- | --- | --- | --- | --- | --- | --- | --- | --- | --- | --- | --- | --- | --- | --- | --- | --- | --- | --- | --- | --- | --- | --- | --- | --- |
| 18. | Touched or fondled your sibling (stepsibling) in a sexual way.  Please check all ages that apply.   \| 1 \| 2 \| 3 \| 4 \| 5 \| 6 \| 7 \| 8 \| 9 \| 10 \| 11 \| 12 \| 13 \| 14 \| 15 \| 16 \| 17 \| 18 \| \| --- \| --- \| --- \| --- \| --- \| --- \| --- \| --- \| --- \| --- \| --- \| --- \| --- \| --- \| --- \| --- \| --- \| --- \| \|  \|  \|  \|  \|  \|  \|  \|  \|  \|  \|  \|  \|  \|  \|  \|  \|  \|  \| | 🔿  Yes_1_ | 🔿  No_0_ |  |

|  | **Sometimes adults or older individuals NOT living in the house do hurtful things to you.**  **If this happened during your childhood (first 18 years of your life) please provide your best estimates of your age at the time(s) of occurrence.**  **Please check all ages that apply.** | | |  | |  | |
| --- | --- | --- | --- | --- | --- | --- | --- |
| 19. | | Had you touch their body in a sexual way.  Please check all ages that apply.   \| 1 \| 2 \| 3 \| 4 \| 5 \| 6 \| 7 \| 8 \| 9 \| 10 \| 11 \| 12 \| 13 \| 14 \| 15 \| 16 \| 17 \| 18 \| \| --- \| --- \| --- \| --- \| --- \| --- \| --- \| --- \| --- \| --- \| --- \| --- \| --- \| --- \| --- \| --- \| --- \| --- \| \|  \|  \|  \|  \|  \|  \|  \|  \|  \|  \|  \|  \|  \|  \|  \|  \|  \|  \| | 🔿  Yes_1_ | | 🔿  No_0_ | |  |
| 20. | | Actually had sexual intercourse (oral, anal or vaginal) with you.  Please check all ages that apply.   \| 1 \| 2 \| 3 \| 4 \| 5 \| 6 \| 7 \| 8 \| 9 \| 10 \| 11 \| 12 \| 13 \| 14 \| 15 \| 16 \| 17 \| 18 \| \| --- \| --- \| --- \| --- \| --- \| --- \| --- \| --- \| --- \| --- \| --- \| --- \| --- \| --- \| --- \| --- \| --- \| --- \| \|  \|  \|  \|  \|  \|  \|  \|  \|  \|  \|  \|  \|  \|  \|  \|  \|  \|  \| | 🔿  Yes_1_ | | 🔿  No_0_ | |  |

|  | **Sometimes intense arguments or physical fights occur between parents, stepparents or other adults (boyfriends, girlfriends, grandparents) living in the household.**  **If this happened during your childhood (first 18 years of your life) please provide your best estimates of your age at the time(s) of occurrence.**  **Please check all ages that apply.** | | |  | |  | |
| --- | --- | --- | --- | --- | --- | --- | --- |
| 21. | | Saw adults living in the household push, grab, slap or throw something at your mother (stepmother, grandmother).  Please check all ages that apply.   \| 1 \| 2 \| 3 \| 4 \| 5 \| 6 \| 7 \| 8 \| 9 \| 10 \| 11 \| 12 \| 13 \| 14 \| 15 \| 16 \| 17 \| 18 \| \| --- \| --- \| --- \| --- \| --- \| --- \| --- \| --- \| --- \| --- \| --- \| --- \| --- \| --- \| --- \| --- \| --- \| --- \| \|  \|  \|  \|  \|  \|  \|  \|  \|  \|  \|  \|  \|  \|  \|  \|  \|  \|  \| | 🔿  Yes_1_ | | 🔿  No_._ | |  |
| 22. | | Saw adults living in the household hit your mother (stepmother, grandmother) so hard that it left marks for more than a few minutes.  Please check all ages that apply.   \| 1 \| 2 \| 3 \| 4 \| 5 \| 6 \| 7 \| 8 \| 9 \| 10 \| 11 \| 12 \| 13 \| 14 \| 15 \| 16 \| 17 \| 18 \| \| --- \| --- \| --- \| --- \| --- \| --- \| --- \| --- \| --- \| --- \| --- \| --- \| --- \| --- \| --- \| --- \| --- \| --- \| \|  \|  \|  \|  \|  \|  \|  \|  \|  \|  \|  \|  \|  \|  \|  \|  \|  \|  \| | 🔿  Yes_1_ | | 🔿  No_0_ | |  |
| 23. | | Saw adults living in the household hit your mother (stepmother, grandmother) so hard, or intentionally harm her in some way, that she received or should have received medical attention.  Please check all ages that apply.   \| 1 \| 2 \| 3 \| 4 \| 5 \| 6 \| 7 \| 8 \| 9 \| 10 \| 11 \| 12 \| 13 \| 14 \| 15 \| 16 \| 17 \| 18 \| \| --- \| --- \| --- \| --- \| --- \| --- \| --- \| --- \| --- \| --- \| --- \| --- \| --- \| --- \| --- \| --- \| --- \| --- \| \|  \|  \|  \|  \|  \|  \|  \|  \|  \|  \|  \|  \|  \|  \|  \|  \|  \|  \| | 🔿  Yes_._ | | 🔿  No_._ | |  |
| 24. | | Saw adults living in the household push, grab, slap or throw something at your father (stepfather, grandfather).  Please check all ages that apply.   \| 1 \| 2 \| 3 \| 4 \| 5 \| 6 \| 7 \| 8 \| 9 \| 10 \| 11 \| 12 \| 13 \| 14 \| 15 \| 16 \| 17 \| 18 \| \| --- \| --- \| --- \| --- \| --- \| --- \| --- \| --- \| --- \| --- \| --- \| --- \| --- \| --- \| --- \| --- \| --- \| --- \| \|  \|  \|  \|  \|  \|  \|  \|  \|  \|  \|  \|  \|  \|  \|  \|  \|  \|  \| | 🔿  Yes_1_ | | 🔿  No_._ | |  |
| 25. | | Saw adults living in the household hit your father (stepfather, grandfather) so hard that it left marks for more than a few minutes.  Please check all ages that apply.   \| 1 \| 2 \| 3 \| 4 \| 5 \| 6 \| 7 \| 8 \| 9 \| 10 \| 11 \| 12 \| 13 \| 14 \| 15 \| 16 \| 17 \| 18 \| \| --- \| --- \| --- \| --- \| --- \| --- \| --- \| --- \| --- \| --- \| --- \| --- \| --- \| --- \| --- \| --- \| --- \| --- \| \|  \|  \|  \|  \|  \|  \|  \|  \|  \|  \|  \|  \|  \|  \|  \|  \|  \|  \| | 🔿  Yes_1_ | | 🔿  No_._ | |  |

|  | **Sometimes children your own age or older do hurtful things like bully or harass you.**  **If this happened during your childhood (first 18 years of your life) please provide your best estimates of your age at the time(s) of occurrence.**  **Please check all ages that apply.** | | |  | |  | |
| --- | --- | --- | --- | --- | --- | --- | --- |
| 26. | | Swore at you, called you names, said insulting things like your “fat”, “ugly”, “stupid”, etc. more than a few times a year.  Please check all ages that apply.   \| 1 \| 2 \| 3 \| 4 \| 5 \| 6 \| 7 \| 8 \| 9 \| 10 \| 11 \| 12 \| 13 \| 14 \| 15 \| 16 \| 17 \| 18 \| \| --- \| --- \| --- \| --- \| --- \| --- \| --- \| --- \| --- \| --- \| --- \| --- \| --- \| --- \| --- \| --- \| --- \| --- \| \|  \|  \|  \|  \|  \|  \|  \|  \|  \|  \|  \|  \|  \|  \|  \|  \|  \|  \| | 🔿  Yes_1_ | | 🔿  No_0_ | |  |
| 27. | | Said hurtful things that made you feel bad, embarrassed or humiliated more than a few times a year.  Please check all ages that apply.   \| 1 \| 2 \| 3 \| 4 \| 5 \| 6 \| 7 \| 8 \| 9 \| 10 \| 11 \| 12 \| 13 \| 14 \| 15 \| 16 \| 17 \| 18 \| \| --- \| --- \| --- \| --- \| --- \| --- \| --- \| --- \| --- \| --- \| --- \| --- \| --- \| --- \| --- \| --- \| --- \| --- \| \|  \|  \|  \|  \|  \|  \|  \|  \|  \|  \|  \|  \|  \|  \|  \|  \|  \|  \| | 🔿  Yes_1_ | | 🔿  No_0_ | |  |
| 28. | | Said things behind your back, posted derogatory messages about you, or spread rumors about you.  Please check all ages that apply.   \| 1 \| 2 \| 3 \| 4 \| 5 \| 6 \| 7 \| 8 \| 9 \| 10 \| 11 \| 12 \| 13 \| 14 \| 15 \| 16 \| 17 \| 18 \| \| --- \| --- \| --- \| --- \| --- \| --- \| --- \| --- \| --- \| --- \| --- \| --- \| --- \| --- \| --- \| --- \| --- \| --- \| \|  \|  \|  \|  \|  \|  \|  \|  \|  \|  \|  \|  \|  \|  \|  \|  \|  \|  \| | 🔿  Yes_1_ | | 🔿  No_0_ | |  |
| 29. | | Intentionally excluded you from activities or groups.  Please check all ages that apply.   \| 1 \| 2 \| 3 \| 4 \| 5 \| 6 \| 7 \| 8 \| 9 \| 10 \| 11 \| 12 \| 13 \| 14 \| 15 \| 16 \| 17 \| 18 \| \| --- \| --- \| --- \| --- \| --- \| --- \| --- \| --- \| --- \| --- \| --- \| --- \| --- \| --- \| --- \| --- \| --- \| --- \| \|  \|  \|  \|  \|  \|  \|  \|  \|  \|  \|  \|  \|  \|  \|  \|  \|  \|  \| | 🔿  Yes_1_ | | 🔿  No_0_ | |  |
| 30. | | Acted in a way that made you afraid that you might be physically hurt.  Please check all ages that apply.   \| 1 \| 2 \| 3 \| 4 \| 5 \| 6 \| 7 \| 8 \| 9 \| 10 \| 11 \| 12 \| 13 \| 14 \| 15 \| 16 \| 17 \| 18 \| \| --- \| --- \| --- \| --- \| --- \| --- \| --- \| --- \| --- \| --- \| --- \| --- \| --- \| --- \| --- \| --- \| --- \| --- \| \|  \|  \|  \|  \|  \|  \|  \|  \|  \|  \|  \|  \|  \|  \|  \|  \|  \|  \| | 🔿  Yes_._ | | 🔿  No_.._ | |  |
| 31. | | Threatened you in order to take your money or possessions.  Please check all ages that apply.   \| 1 \| 2 \| 3 \| 4 \| 5 \| 6 \| 7 \| 8 \| 9 \| 10 \| 11 \| 12 \| 13 \| 14 \| 15 \| 16 \| 17 \| 18 \| \| --- \| --- \| --- \| --- \| --- \| --- \| --- \| --- \| --- \| --- \| --- \| --- \| --- \| --- \| --- \| --- \| --- \| --- \| \|  \|  \|  \|  \|  \|  \|  \|  \|  \|  \|  \|  \|  \|  \|  \|  \|  \|  \| | 🔿  Yes_1_ | | 🔿  No_0_ | |  |

| 32. | Forced or threatened you to do things that you did not want to do.  Please check all ages that apply.   \| 1 \| 2 \| 3 \| 4 \| 5 \| 6 \| 7 \| 8 \| 9 \| 10 \| 11 \| 12 \| 13 \| 14 \| 15 \| 16 \| 17 \| 18 \| \| --- \| --- \| --- \| --- \| --- \| --- \| --- \| --- \| --- \| --- \| --- \| --- \| --- \| --- \| --- \| --- \| --- \| --- \| \|  \|  \|  \|  \|  \|  \|  \|  \|  \|  \|  \|  \|  \|  \|  \|  \|  \|  \| | 🔿  Yes_1_ | 🔿  No_0_ |  |
| --- | --- | --- | --- | --- | --- | --- | --- | --- | --- | --- | --- | --- | --- | --- | --- | --- | --- | --- | --- | --- | --- | --- | --- | --- | --- | --- | --- | --- | --- | --- | --- | --- | --- | --- | --- | --- | --- | --- | --- | --- |
| 33. | Intentionally pushed, grabbed, shoved, slapped, pinched, punched, or kicked you.  Please check all ages that apply.   \| 1 \| 2 \| 3 \| 4 \| 5 \| 6 \| 7 \| 8 \| 9 \| 10 \| 11 \| 12 \| 13 \| 14 \| 15 \| 16 \| 17 \| 18 \| \| --- \| --- \| --- \| --- \| --- \| --- \| --- \| --- \| --- \| --- \| --- \| --- \| --- \| --- \| --- \| --- \| --- \| --- \| \|  \|  \|  \|  \|  \|  \|  \|  \|  \|  \|  \|  \|  \|  \|  \|  \|  \|  \| | 🔿  Yes_1_ | 🔿  No_0_ |  |
| 34. | Hit you so hard that it left marks for more than a few minutes.  Please check all ages that apply.   \| 1 \| 2 \| 3 \| 4 \| 5 \| 6 \| 7 \| 8 \| 9 \| 10 \| 11 \| 12 \| 13 \| 14 \| 15 \| 16 \| 17 \| 18 \| \| --- \| --- \| --- \| --- \| --- \| --- \| --- \| --- \| --- \| --- \| --- \| --- \| --- \| --- \| --- \| --- \| --- \| --- \| \|  \|  \|  \|  \|  \|  \|  \|  \|  \|  \|  \|  \|  \|  \|  \|  \|  \|  \| | 🔿  Yes_1_ | 🔿  No_0_ |  |
| 35. | Hit you so hard, or intentionally harmed you in some way, that you received or should have received medical attention.  Please check all ages that apply.   \| 1 \| 2 \| 3 \| 4 \| 5 \| 6 \| 7 \| 8 \| 9 \| 10 \| 11 \| 12 \| 13 \| 14 \| 15 \| 16 \| 17 \| 18 \| \| --- \| --- \| --- \| --- \| --- \| --- \| --- \| --- \| --- \| --- \| --- \| --- \| --- \| --- \| --- \| --- \| --- \| --- \| \|  \|  \|  \|  \|  \|  \|  \|  \|  \|  \|  \|  \|  \|  \|  \|  \|  \|  \| | 🔿  Yes_1_ | 🔿  No_0_ |  |
| 36. | Forced you to engage in sexual activity against your will.  Please check all ages that apply.   \| 1 \| 2 \| 3 \| 4 \| 5 \| 6 \| 7 \| 8 \| 9 \| 10 \| 11 \| 12 \| 13 \| 14 \| 15 \| 16 \| 17 \| 18 \| \| --- \| --- \| --- \| --- \| --- \| --- \| --- \| --- \| --- \| --- \| --- \| --- \| --- \| --- \| --- \| --- \| --- \| --- \| \|  \|  \|  \|  \|  \|  \|  \|  \|  \|  \|  \|  \|  \|  \|  \|  \|  \|  \| | 🔿  Yes_1_ | 🔿  No_0_ |  |
| 37. | Forced you to do things sexually that you did not want to do.  Please check all ages that apply.   \| 1 \| 2 \| 3 \| 4 \| 5 \| 6 \| 7 \| 8 \| 9 \| 10 \| 11 \| 12 \| 13 \| 14 \| 15 \| 16 \| 17 \| 18 \| \| --- \| --- \| --- \| --- \| --- \| --- \| --- \| --- \| --- \| --- \| --- \| --- \| --- \| --- \| --- \| --- \| --- \| --- \| \|  \|  \|  \|  \|  \|  \|  \|  \|  \|  \|  \|  \|  \|  \|  \|  \|  \|  \| | 🔿  Yes_1_ | 🔿  No |  |

|  | Please indicate if the following happened during your childhood (first 18 years of your life). Please provide your best estimates of your age at the time(s) of occurrence.  **Please check all ages that apply.** | | |  | |  | |
| --- | --- | --- | --- | --- | --- | --- | --- |
| 38. | | You felt that your mother or other important maternal figure was present in the household but emotionally unavailable to you for a variety of reasons like drugs, alcohol, workaholic, having an affair, heedlessly pursuing their own goals.  Please check all ages that apply.   \| 1 \| 2 \| 3 \| 4 \| 5 \| 6 \| 7 \| 8 \| 9 \| 10 \| 11 \| 12 \| 13 \| 14 \| 15 \| 16 \| 17 \| 18 \| \| --- \| --- \| --- \| --- \| --- \| --- \| --- \| --- \| --- \| --- \| --- \| --- \| --- \| --- \| --- \| --- \| --- \| --- \| \|  \|  \|  \|  \|  \|  \|  \|  \|  \|  \|  \|  \|  \|  \|  \|  \|  \|  \| | 🔿  Yes_1_ | | 🔿  No_0_ | |  |
| 39. | | You felt that your father or other important paternal figure was present in the household but emotionally unavailable to you for a variety of reasons like drugs, alcohol, workaholic, having an affair, heedlessly pursuing their own goals.  Please check all ages that apply.   \| 1 \| 2 \| 3 \| 4 \| 5 \| 6 \| 7 \| 8 \| 9 \| 10 \| 11 \| 12 \| 13 \| 14 \| 15 \| 16 \| 17 \| 18 \| \| --- \| --- \| --- \| --- \| --- \| --- \| --- \| --- \| --- \| --- \| --- \| --- \| --- \| --- \| --- \| --- \| --- \| --- \| \|  \|  \|  \|  \|  \|  \|  \|  \|  \|  \|  \|  \|  \|  \|  \|  \|  \|  \| | 🔿  Yes_1_ | | 🔿  No_0_ | |  |
| 40. | | A parent or other important parental figure was very difficult to please.  Please check all ages that apply.   \| 1 \| 2 \| 3 \| 4 \| 5 \| 6 \| 7 \| 8 \| 9 \| 10 \| 11 \| 12 \| 13 \| 14 \| 15 \| 16 \| 17 \| 18 \| \| --- \| --- \| --- \| --- \| --- \| --- \| --- \| --- \| --- \| --- \| --- \| --- \| --- \| --- \| --- \| --- \| --- \| --- \| \|  \|  \|  \|  \|  \|  \|  \|  \|  \|  \|  \|  \|  \|  \|  \|  \|  \|  \| | 🔿  Yes_1_ | | 🔿  No_0_ | |  |
| 41. | | A parent or other important parental figure did not have the time or interest to talk to you.  Please check all ages that apply.   \| 1 \| 2 \| 3 \| 4 \| 5 \| 6 \| 7 \| 8 \| 9 \| 10 \| 11 \| 12 \| 13 \| 14 \| 15 \| 16 \| 17 \| 18 \| \| --- \| --- \| --- \| --- \| --- \| --- \| --- \| --- \| --- \| --- \| --- \| --- \| --- \| --- \| --- \| --- \| --- \| --- \| \|  \|  \|  \|  \|  \|  \|  \|  \|  \|  \|  \|  \|  \|  \|  \|  \|  \|  \| | 🔿  Yes_1_ | | 🔿  No_0_ | |  |
| 42. | | One or more individuals in your family made you feel loved.  Please check all ages that apply.   \| 1 \| 2 \| 3 \| 4 \| 5 \| 6 \| 7 \| 8 \| 9 \| 10 \| 11 \| 12 \| 13 \| 14 \| 15 \| 16 \| 17 \| 18 \| \| --- \| --- \| --- \| --- \| --- \| --- \| --- \| --- \| --- \| --- \| --- \| --- \| --- \| --- \| --- \| --- \| --- \| --- \| \|  \|  \|  \|  \|  \|  \|  \|  \|  \|  \|  \|  \|  \|  \|  \|  \|  \|  \| | 🔿  Yes_1_ | | 🔿  No_0_ | |  |
| 43. | | One or more individuals in your family helped you feel important or special.  Please check all ages that apply.   \| 1 \| 2 \| 3 \| 4 \| 5 \| 6 \| 7 \| 8 \| 9 \| 10 \| 11 \| 12 \| 13 \| 14 \| 15 \| 16 \| 17 \| 18 \| \| --- \| --- \| --- \| --- \| --- \| --- \| --- \| --- \| --- \| --- \| --- \| --- \| --- \| --- \| --- \| --- \| --- \| --- \| \|  \|  \|  \|  \|  \|  \|  \|  \|  \|  \|  \|  \|  \|  \|  \|  \|  \|  \| | 🔿  Yes_1_ | | 🔿  No_0_ | |  |
| 44. | | One or more individuals in your family were there to take care of you and protect you.  Please check all ages that apply.   \| 1 \| 2 \| 3 \| 4 \| 5 \| 6 \| 7 \| 8 \| 9 \| 10 \| 11 \| 12 \| 13 \| 14 \| 15 \| 16 \| 17 \| 18 \| \| --- \| --- \| --- \| --- \| --- \| --- \| --- \| --- \| --- \| --- \| --- \| --- \| --- \| --- \| --- \| --- \| --- \| --- \| \|  \|  \|  \|  \|  \|  \|  \|  \|  \|  \|  \|  \|  \|  \|  \|  \|  \|  \| | 🔿  Yes_1_ | | 🔿  No_0_ | |  |
| 45. | | One or more individuals in your family were there to take you to the doctor or Emergency Room if the need ever arose, or would have if needed.  Please check all ages that apply.   \| 1 \| 2 \| 3 \| 4 \| 5 \| 6 \| 7 \| 8 \| 9 \| 10 \| 11 \| 12 \| 13 \| 14 \| 15 \| 16 \| 17 \| 18 \| \| --- \| --- \| --- \| --- \| --- \| --- \| --- \| --- \| --- \| --- \| --- \| --- \| --- \| --- \| --- \| --- \| --- \| --- \| \|  \|  \|  \|  \|  \|  \|  \|  \|  \|  \|  \|  \|  \|  \|  \|  \|  \|  \| | 🔿  Yes_1_ | | 🔿  No_0_ | |  |

|  | **Please indicate if the following statements were true about you and your family during your childhood, and your age at the time(s) you felt this to be true.**  **Please check all ages that apply.** | | |  | |  | |
| --- | --- | --- | --- | --- | --- | --- | --- |
| 46. | | You didn’t have enough to eat.  Please check all ages that apply.   \| 1 \| 2 \| 3 \| 4 \| 5 \| 6 \| 7 \| 8 \| 9 \| 10 \| 11 \| 12 \| 13 \| 14 \| 15 \| 16 \| 17 \| 18 \| \| --- \| --- \| --- \| --- \| --- \| --- \| --- \| --- \| --- \| --- \| --- \| --- \| --- \| --- \| --- \| --- \| --- \| --- \| \|  \|  \|  \|  \|  \|  \|  \|  \|  \|  \|  \|  \|  \|  \|  \|  \|  \|  \| | 🔿  Yes_1_ | | 🔿  No_0_ | |  |
| 47. | | You had to wear dirty clothes.  Please check all ages that apply.   \| 1 \| 2 \| 3 \| 4 \| 5 \| 6 \| 7 \| 8 \| 9 \| 10 \| 11 \| 12 \| 13 \| 14 \| 15 \| 16 \| 17 \| 18 \| \| --- \| --- \| --- \| --- \| --- \| --- \| --- \| --- \| --- \| --- \| --- \| --- \| --- \| --- \| --- \| --- \| --- \| --- \| \|  \|  \|  \|  \|  \|  \|  \|  \|  \|  \|  \|  \|  \|  \|  \|  \|  \|  \| | 🔿  Yes_1_ | | 🔿  No_0_ | |  |
| 48. | | You felt that you had to shoulder adult responsibilities.  Please check all ages that apply.   \| 1 \| 2 \| 3 \| 4 \| 5 \| 6 \| 7 \| 8 \| 9 \| 10 \| 11 \| 12 \| 13 \| 14 \| 15 \| 16 \| 17 \| 18 \| \| --- \| --- \| --- \| --- \| --- \| --- \| --- \| --- \| --- \| --- \| --- \| --- \| --- \| --- \| --- \| --- \| --- \| --- \| \|  \|  \|  \|  \|  \|  \|  \|  \|  \|  \|  \|  \|  \|  \|  \|  \|  \|  \| | 🔿  Yes_1_ | | 🔿  No_0_ | |  |
| 49. | | You felt that your family was under severe financial pressure.  Please check all ages that apply.   \| 1 \| 2 \| 3 \| 4 \| 5 \| 6 \| 7 \| 8 \| 9 \| 10 \| 11 \| 12 \| 13 \| 14 \| 15 \| 16 \| 17 \| 18 \| \| --- \| --- \| --- \| --- \| --- \| --- \| --- \| --- \| --- \| --- \| --- \| --- \| --- \| --- \| --- \| --- \| --- \| --- \| \|  \|  \|  \|  \|  \|  \|  \|  \|  \|  \|  \|  \|  \|  \|  \|  \|  \|  \| | 🔿  Yes_1_ | | 🔿  No_0_ | |  |
| 50. | | One or more individuals kept important secrets or facts from you.  Please check all ages that apply.   \| 1 \| 2 \| 3 \| 4 \| 5 \| 6 \| 7 \| 8 \| 9 \| 10 \| 11 \| 12 \| 13 \| 14 \| 15 \| 16 \| 17 \| 18 \| \| --- \| --- \| --- \| --- \| --- \| --- \| --- \| --- \| --- \| --- \| --- \| --- \| --- \| --- \| --- \| --- \| --- \| --- \| \|  \|  \|  \|  \|  \|  \|  \|  \|  \|  \|  \|  \|  \|  \|  \|  \|  \|  \| | 🔿  Yes_1_ | | 🔿  No_0_ | |  |
| 51. | | People in your family looked out for each other.  Please check all ages that apply.   \| 1 \| 2 \| 3 \| 4 \| 5 \| 6 \| 7 \| 8 \| 9 \| 10 \| 11 \| 12 \| 13 \| 14 \| 15 \| 16 \| 17 \| 18 \| \| --- \| --- \| --- \| --- \| --- \| --- \| --- \| --- \| --- \| --- \| --- \| --- \| --- \| --- \| --- \| --- \| --- \| --- \| \|  \|  \|  \|  \|  \|  \|  \|  \|  \|  \|  \|  \|  \|  \|  \|  \|  \|  \| | 🔿  Yes_1_ | | 🔿  No_0_ | |  |
| 52. | | Your family was a source of strength and support.  Please check all ages that apply.   \| 1 \| 2 \| 3 \| 4 \| 5 \| 6 \| 7 \| 8 \| 9 \| 10 \| 11 \| 12 \| 13 \| 14 \| 15 \| 16 \| 17 \| 18 \| \| --- \| --- \| --- \| --- \| --- \| --- \| --- \| --- \| --- \| --- \| --- \| --- \| --- \| --- \| --- \| --- \| --- \| --- \| \|  \|  \|  \|  \|  \|  \|  \|  \|  \|  \|  \|  \|  \|  \|  \|  \|  \|  \| | 🔿  Yes_1_ | | 🔿  No_0_ | |  |
