# Supplementary material for: The ‘Maltreatment and Abuse Chronology of Exposure’ (MACE) Scale for the Retrospective Assessment of Abuse and Neglect During Development
Source: PLoS One. 2015 Feb 25;10(2):e0117423. doi: 10.1371/journal.pone.0117423 (PMC4340880; doi:10.1371/journal.pone.0117423)
Supplement: S3 File — Includes several unscored items. This scale is most useful for researchers endeavoring to develop their own version of the MACE for use with other populations. (DOCX) [file pone.0117423.s012.docx]

MACE-X

|  | **Sometimes parents, stepparents or other adults living in the house do hurtful things.**  **If this happened during your childhood (first 18 years of your life) please provide your best estimate of your age at the time(s) of occurrence.**  **Please check all ages that apply.** | |  |  | |  |
| --- | --- | --- | --- | --- | --- | --- |
|  | | *For example item 1.* Swore at you, called you names, said insulting things like your “fat”, “ugly”, “stupid”, etc. more than a few times a year.  *If at ages 6-8 your father swore at you and at ages 8-10 your mother insulted you, and at age 17 your mother’s new live-in boyfriend called you names; you would check off as follows:*   \| 1 \| 2 \| 3 \| 4 \| 5 \| 6 \| 7 \| 8 \| 9 \| 10 \| 11 \| 12 \| 13 \| 14 \| 15 \| 16 \| 17 \| 18 \| \| --- \| --- \| --- \| --- \| --- \| --- \| --- \| --- \| --- \| --- \| --- \| --- \| --- \| --- \| --- \| --- \| --- \| --- \| \|  \|  \|  \|  \|  \| ✓ \| ✓ \| ✓ \| ✓ \| ✓ \|  \|  \|  \|  \|  \|  \| ✓ \|  \| | ⚫  Yes | 🔿  No |  | |
| 1. | | Swore at you, called you names, said insulting things like your “fat”, “ugly”, “stupid”, etc. more than a few times a year.  Please check all ages that apply.   \| 1 \| 2 \| 3 \| 4 \| 5 \| 6 \| 7 \| 8 \| 9 \| 10 \| 11 \| 12 \| 13 \| 14 \| 15 \| 16 \| 17 \| 18 \| \| --- \| --- \| --- \| --- \| --- \| --- \| --- \| --- \| --- \| --- \| --- \| --- \| --- \| --- \| --- \| --- \| --- \| --- \| \|  \|  \|  \|  \|  \|  \|  \|  \|  \|  \|  \|  \|  \|  \|  \|  \|  \|  \| | 🔿  Yes_1_ | 🔿  No_0_ |  | |
| 2. | | Said hurtful things that made you feel bad, embarrassed or humiliated more than a few times a year.  Please check all ages that apply.   \| 1 \| 2 \| 3 \| 4 \| 5 \| 6 \| 7 \| 8 \| 9 \| 10 \| 11 \| 12 \| 13 \| 14 \| 15 \| 16 \| 17 \| 18 \| \| --- \| --- \| --- \| --- \| --- \| --- \| --- \| --- \| --- \| --- \| --- \| --- \| --- \| --- \| --- \| --- \| --- \| --- \| \|  \|  \|  \|  \|  \|  \|  \|  \|  \|  \|  \|  \|  \|  \|  \|  \|  \|  \| | 🔿  Yes_1_ | 🔿  No_0_ |  | |
| 3. | | Yelled or screamed at you more than a few times per year.  Please check all ages that apply.   \| 1 \| 2 \| 3 \| 4 \| 5 \| 6 \| 7 \| 8 \| 9 \| 10 \| 11 \| 12 \| 13 \| 14 \| 15 \| 16 \| 17 \| 18 \| \| --- \| --- \| --- \| --- \| --- \| --- \| --- \| --- \| --- \| --- \| --- \| --- \| --- \| --- \| --- \| --- \| --- \| --- \| \|  \|  \|  \|  \|  \|  \|  \|  \|  \|  \|  \|  \|  \|  \|  \|  \|  \|  \| | 🔿  Yes_1_ | 🔿  No_0_ |  | |
| 4. | | Acted in a way that made you afraid that you might be physically hurt.  Please check all ages that apply.   \| 1 \| 2 \| 3 \| 4 \| 5 \| 6 \| 7 \| 8 \| 9 \| 10 \| 11 \| 12 \| 13 \| 14 \| 15 \| 16 \| 17 \| 18 \| \| --- \| --- \| --- \| --- \| --- \| --- \| --- \| --- \| --- \| --- \| --- \| --- \| --- \| --- \| --- \| --- \| --- \| --- \| \|  \|  \|  \|  \|  \|  \|  \|  \|  \|  \|  \|  \|  \|  \|  \|  \|  \|  \|   Please indicate if this made you feel helpless or terrified. | 🔿  Yes_1_  ❒  Helpless | 🔿  No_0_  ❒  Terrified |  | |
| 5. | | Threatened to leave or abandon you.  Please check all ages that apply.   \| 1 \| 2 \| 3 \| 4 \| 5 \| 6 \| 7 \| 8 \| 9 \| 10 \| 11 \| 12 \| 13 \| 14 \| 15 \| 16 \| 17 \| 18 \| \| --- \| --- \| --- \| --- \| --- \| --- \| --- \| --- \| --- \| --- \| --- \| --- \| --- \| --- \| --- \| --- \| --- \| --- \| \|  \|  \|  \|  \|  \|  \|  \|  \|  \|  \|  \|  \|  \|  \|  \|  \|  \|  \|   Please indicate if this made you feel helpless or terrified. | 🔿  Yes_1_  ❒  Helpless | 🔿  No_0_  ❒  Terrified |  | |
| 6. | | Locked you in a closet, attic, basement or garage.  Please check all ages that apply.   \| 1 \| 2 \| 3 \| 4 \| 5 \| 6 \| 7 \| 8 \| 9 \| 10 \| 11 \| 12 \| 13 \| 14 \| 15 \| 16 \| 17 \| 18 \| \| --- \| --- \| --- \| --- \| --- \| --- \| --- \| --- \| --- \| --- \| --- \| --- \| --- \| --- \| --- \| --- \| --- \| --- \| \|  \|  \|  \|  \|  \|  \|  \|  \|  \|  \|  \|  \|  \|  \|  \|  \|  \|  \|   Please indicate if this made you feel helpless or terrified. | 🔿  Yes_1_  ❒  Helpless | 🔿  No_0_  ❒  Terrified |  | |

| 7. | Intentionally pushed, grabbed, shoved, slapped, pinched, punched or kicked you.  Please check all ages that apply.   \| 1 \| 2 \| 3 \| 4 \| 5 \| 6 \| 7 \| 8 \| 9 \| 10 \| 11 \| 12 \| 13 \| 14 \| 15 \| 16 \| 17 \| 18 \| \| --- \| --- \| --- \| --- \| --- \| --- \| --- \| --- \| --- \| --- \| --- \| --- \| --- \| --- \| --- \| --- \| --- \| --- \| \|  \|  \|  \|  \|  \|  \|  \|  \|  \|  \|  \|  \|  \|  \|  \|  \|  \|  \|   Please indicate if this made you feel helpless or terrified. | 🔿  Yes_1_  ❒  Helpless | 🔿  No_0_  ❒  Terrified |  |
| --- | --- | --- | --- | --- | --- | --- | --- | --- | --- | --- | --- | --- | --- | --- | --- | --- | --- | --- | --- | --- | --- | --- | --- | --- | --- | --- | --- | --- | --- | --- | --- | --- | --- | --- | --- | --- | --- | --- | --- | --- |
| 8. | Hit you so hard that it left marks for more than a few minutes.  Please check all ages that apply.   \| 1 \| 2 \| 3 \| 4 \| 5 \| 6 \| 7 \| 8 \| 9 \| 10 \| 11 \| 12 \| 13 \| 14 \| 15 \| 16 \| 17 \| 18 \| \| --- \| --- \| --- \| --- \| --- \| --- \| --- \| --- \| --- \| --- \| --- \| --- \| --- \| --- \| --- \| --- \| --- \| --- \| \|  \|  \|  \|  \|  \|  \|  \|  \|  \|  \|  \|  \|  \|  \|  \|  \|  \|  \|   Please indicate if this made you feel helpless or terrified. | 🔿  Yes_1_  ❒  Helpless | 🔿  No_0_  ❒  Terrified |  |
| 9. | Hit you so hard, or intentionally harmed you in some way, that you received or should have received medical attention.  Please check all ages that apply.   \| 1 \| 2 \| 3 \| 4 \| 5 \| 6 \| 7 \| 8 \| 9 \| 10 \| 11 \| 12 \| 13 \| 14 \| 15 \| 16 \| 17 \| 18 \| \| --- \| --- \| --- \| --- \| --- \| --- \| --- \| --- \| --- \| --- \| --- \| --- \| --- \| --- \| --- \| --- \| --- \| --- \| \|  \|  \|  \|  \|  \|  \|  \|  \|  \|  \|  \|  \|  \|  \|  \|  \|  \|  \|   Please indicate if this made you feel helpless or terrified. | 🔿  Yes_1_  ❒  Helpless | 🔿  No_0_  ❒  Terrified |  |
| 10. | Spanked you on your buttocks, arms or legs.  Please check all ages that apply.   \| 1 \| 2 \| 3 \| 4 \| 5 \| 6 \| 7 \| 8 \| 9 \| 10 \| 11 \| 12 \| 13 \| 14 \| 15 \| 16 \| 17 \| 18 \| \| --- \| --- \| --- \| --- \| --- \| --- \| --- \| --- \| --- \| --- \| --- \| --- \| --- \| --- \| --- \| --- \| --- \| --- \| \|  \|  \|  \|  \|  \|  \|  \|  \|  \|  \|  \|  \|  \|  \|  \|  \|  \|  \|   Please indicate if this made you feel helpless or terrified. | 🔿  Yes_1_  ❒  Helpless | 🔿  No_0_  ❒  Terrified |  |
| 11. | Spanked you on your bare (unclothed) buttocks.  Please check all ages that apply.   \| 1 \| 2 \| 3 \| 4 \| 5 \| 6 \| 7 \| 8 \| 9 \| 10 \| 11 \| 12 \| 13 \| 14 \| 15 \| 16 \| 17 \| 18 \| \| --- \| --- \| --- \| --- \| --- \| --- \| --- \| --- \| --- \| --- \| --- \| --- \| --- \| --- \| --- \| --- \| --- \| --- \| \|  \|  \|  \|  \|  \|  \|  \|  \|  \|  \|  \|  \|  \|  \|  \|  \|  \|  \|   Please indicate if this made you feel helpless or terrified. | 🔿  Yes_1_  ❒  Helpless | 🔿  No_0_  ❒  Terrified |  |
| 12. | Spanked you with an object such as a strap, belt, brush, paddle, rod, etc.  Please check all ages that apply.   \| 1 \| 2 \| 3 \| 4 \| 5 \| 6 \| 7 \| 8 \| 9 \| 10 \| 11 \| 12 \| 13 \| 14 \| 15 \| 16 \| 17 \| 18 \| \| --- \| --- \| --- \| --- \| --- \| --- \| --- \| --- \| --- \| --- \| --- \| --- \| --- \| --- \| --- \| --- \| --- \| --- \| \|  \|  \|  \|  \|  \|  \|  \|  \|  \|  \|  \|  \|  \|  \|  \|  \|  \|  \|   Please indicate if this made you feel helpless or terrified. | 🔿  Yes_1_  ❒  Helpless | 🔿  No_0_  ❒  Terrified |  |
| 13. | Made inappropriate sexual comments or suggestions to you.  Please check all ages that apply.   \| 1 \| 2 \| 3 \| 4 \| 5 \| 6 \| 7 \| 8 \| 9 \| 10 \| 11 \| 12 \| 13 \| 14 \| 15 \| 16 \| 17 \| 18 \| \| --- \| --- \| --- \| --- \| --- \| --- \| --- \| --- \| --- \| --- \| --- \| --- \| --- \| --- \| --- \| --- \| --- \| --- \| \|  \|  \|  \|  \|  \|  \|  \|  \|  \|  \|  \|  \|  \|  \|  \|  \|  \|  \|   Please indicate if this made you feel helpless or terrified. | 🔿  Yes_1_  ❒  Helpless | 🔿  No_0_  ❒  Terrified |  |

| 14. | Touched or fondled your body in a sexual way.  Please check all ages that apply.   \| 1 \| 2 \| 3 \| 4 \| 5 \| 6 \| 7 \| 8 \| 9 \| 10 \| 11 \| 12 \| 13 \| 14 \| 15 \| 16 \| 17 \| 18 \| \| --- \| --- \| --- \| --- \| --- \| --- \| --- \| --- \| --- \| --- \| --- \| --- \| --- \| --- \| --- \| --- \| --- \| --- \| \|  \|  \|  \|  \|  \|  \|  \|  \|  \|  \|  \|  \|  \|  \|  \|  \|  \|  \|   Please indicate if this made you feel helpless or terrified. | 🔿  Yes_1_  ❒  Helpless | 🔿  No_0_  ❒  Terrified |  |
| --- | --- | --- | --- | --- | --- | --- | --- | --- | --- | --- | --- | --- | --- | --- | --- | --- | --- | --- | --- | --- | --- | --- | --- | --- | --- | --- | --- | --- | --- | --- | --- | --- | --- | --- | --- | --- | --- | --- | --- | --- |
| 15. | Had you touch their body in a sexual way.  Please check all ages that apply.   \| 1 \| 2 \| 3 \| 4 \| 5 \| 6 \| 7 \| 8 \| 9 \| 10 \| 11 \| 12 \| 13 \| 14 \| 15 \| 16 \| 17 \| 18 \| \| --- \| --- \| --- \| --- \| --- \| --- \| --- \| --- \| --- \| --- \| --- \| --- \| --- \| --- \| --- \| --- \| --- \| --- \| \|  \|  \|  \|  \|  \|  \|  \|  \|  \|  \|  \|  \|  \|  \|  \|  \|  \|  \|   Please indicate if this made you feel helpless or terrified. | 🔿  Yes_1_  ❒  Helpless | 🔿  No_0_  ❒  Terrified |  |
| 16. | Attempted to have any type of sexual intercourse (oral, anal or vaginal) with you.  Please check all ages that apply.   \| 1 \| 2 \| 3 \| 4 \| 5 \| 6 \| 7 \| 8 \| 9 \| 10 \| 11 \| 12 \| 13 \| 14 \| 15 \| 16 \| 17 \| 18 \| \| --- \| --- \| --- \| --- \| --- \| --- \| --- \| --- \| --- \| --- \| --- \| --- \| --- \| --- \| --- \| --- \| --- \| --- \| \|  \|  \|  \|  \|  \|  \|  \|  \|  \|  \|  \|  \|  \|  \|  \|  \|  \|  \|   Please indicate if this made you feel helpless or terrified. | 🔿  Yes_1_  ❒  Helpless | 🔿  No_0_  ❒  Terrified |  |
| 17. | Actually had any type of sexual intercourse (oral, anal or vaginal) with you.  Please check all ages that apply.   \| 1 \| 2 \| 3 \| 4 \| 5 \| 6 \| 7 \| 8 \| 9 \| 10 \| 11 \| 12 \| 13 \| 14 \| 15 \| 16 \| 17 \| 18 \| \| --- \| --- \| --- \| --- \| --- \| --- \| --- \| --- \| --- \| --- \| --- \| --- \| --- \| --- \| --- \| --- \| --- \| --- \| \|  \|  \|  \|  \|  \|  \|  \|  \|  \|  \|  \|  \|  \|  \|  \|  \|  \|  \|   Please indicate if this made you feel helpless or terrified. | 🔿  Yes_1_  ❒  Helpless | 🔿  No_0_  ❒  Terrified |  |

|  | **Sometimes parents, stepparents or other adults living in the house do hurtful things to your siblings (brother, sister, stepsiblings).**  **If this happened during your childhood (first 18 years of your life) please provide your best estimates of your age at the time(s) of occurrence.**  **Please check all ages that apply.** | | |  | |  | |
| --- | --- | --- | --- | --- | --- | --- | --- |
| 18. | | Intentionally pushed, grabbed, shoved, slapped, pinched, punched, or kicked your sibling (stepsibling).  Please check all ages that apply.   \| 1 \| 2 \| 3 \| 4 \| 5 \| 6 \| 7 \| 8 \| 9 \| 10 \| 11 \| 12 \| 13 \| 14 \| 15 \| 16 \| 17 \| 18 \| \| --- \| --- \| --- \| --- \| --- \| --- \| --- \| --- \| --- \| --- \| --- \| --- \| --- \| --- \| --- \| --- \| --- \| --- \| \|  \|  \|  \|  \|  \|  \|  \|  \|  \|  \|  \|  \|  \|  \|  \|  \|  \|  \|   Please indicate if this made you feel helpless or terrified. | 🔿  Yes_1_  ❒  Helpless | | 🔿  No_0_  ❒  Terrified | |  |
| 19. | | Hit your sibling (stepsibling) so hard that it left marks for more than a few minutes.  Please check all ages that apply.   \| 1 \| 2 \| 3 \| 4 \| 5 \| 6 \| 7 \| 8 \| 9 \| 10 \| 11 \| 12 \| 13 \| 14 \| 15 \| 16 \| 17 \| 18 \| \| --- \| --- \| --- \| --- \| --- \| --- \| --- \| --- \| --- \| --- \| --- \| --- \| --- \| --- \| --- \| --- \| --- \| --- \| \|  \|  \|  \|  \|  \|  \|  \|  \|  \|  \|  \|  \|  \|  \|  \|  \|  \|  \|   Please indicate if this made you feel helpless or terrified. | 🔿  Yes_1_  ❒  Helpless | | 🔿  No_0_  ❒  Terrified | |  |

| 20. | Hit your sibling (stepsibling) so hard, or intentionally harmed him/her in some way, that he/she received or should have received medical attention.  Please check all ages that apply.   \| 1 \| 2 \| 3 \| 4 \| 5 \| 6 \| 7 \| 8 \| 9 \| 10 \| 11 \| 12 \| 13 \| 14 \| 15 \| 16 \| 17 \| 18 \| \| --- \| --- \| --- \| --- \| --- \| --- \| --- \| --- \| --- \| --- \| --- \| --- \| --- \| --- \| --- \| --- \| --- \| --- \| \|  \|  \|  \|  \|  \|  \|  \|  \|  \|  \|  \|  \|  \|  \|  \|  \|  \|  \|   Please indicate if this made you feel helpless or terrified. | 🔿  Yes_1_  ❒  Helpless | 🔿  No_0_  ❒  Terrified |  |
| --- | --- | --- | --- | --- | --- | --- | --- | --- | --- | --- | --- | --- | --- | --- | --- | --- | --- | --- | --- | --- | --- | --- | --- | --- | --- | --- | --- | --- | --- | --- | --- | --- | --- | --- | --- | --- | --- | --- | --- | --- |
| 21. | Made inappropriate sexual comments or suggestions to your sibling (stepsibling).  Please check all ages that apply.   \| 1 \| 2 \| 3 \| 4 \| 5 \| 6 \| 7 \| 8 \| 9 \| 10 \| 11 \| 12 \| 13 \| 14 \| 15 \| 16 \| 17 \| 18 \| \| --- \| --- \| --- \| --- \| --- \| --- \| --- \| --- \| --- \| --- \| --- \| --- \| --- \| --- \| --- \| --- \| --- \| --- \| \|  \|  \|  \|  \|  \|  \|  \|  \|  \|  \|  \|  \|  \|  \|  \|  \|  \|  \|   Please indicate if this made you feel helpless or terrified. | 🔿  Yes_1_  ❒  Helpless | 🔿  No_0_  ❒  Terrified |  |
| 22. | Touched or fondled your sibling (stepsibling) in a sexual way.  Please check all ages that apply.   \| 1 \| 2 \| 3 \| 4 \| 5 \| 6 \| 7 \| 8 \| 9 \| 10 \| 11 \| 12 \| 13 \| 14 \| 15 \| 16 \| 17 \| 18 \| \| --- \| --- \| --- \| --- \| --- \| --- \| --- \| --- \| --- \| --- \| --- \| --- \| --- \| --- \| --- \| --- \| --- \| --- \| \|  \|  \|  \|  \|  \|  \|  \|  \|  \|  \|  \|  \|  \|  \|  \|  \|  \|  \|   Please indicate if this made you feel helpless or terrified. | 🔿  Yes_1_  ❒  Helpless | 🔿  No_0_  ❒  Terrified |  |
| 23. | Had your sibling (stepsibling) touch their body in a sexual way.  Please check all ages that apply.   \| 1 \| 2 \| 3 \| 4 \| 5 \| 6 \| 7 \| 8 \| 9 \| 10 \| 11 \| 12 \| 13 \| 14 \| 15 \| 16 \| 17 \| 18 \| \| --- \| --- \| --- \| --- \| --- \| --- \| --- \| --- \| --- \| --- \| --- \| --- \| --- \| --- \| --- \| --- \| --- \| --- \| \|  \|  \|  \|  \|  \|  \|  \|  \|  \|  \|  \|  \|  \|  \|  \|  \|  \|  \|   Please indicate if this made you feel helpless or terrified. | 🔿  Yes_1_  ❒  Helpless | 🔿  No_0_  ❒  Terrified |  |
| 24. | Had or attempted to have any type of sexual intercourse (oral, anal or vaginal) with your sibling (stepsibling).  Please check all ages that apply.   \| 1 \| 2 \| 3 \| 4 \| 5 \| 6 \| 7 \| 8 \| 9 \| 10 \| 11 \| 12 \| 13 \| 14 \| 15 \| 16 \| 17 \| 18 \| \| --- \| --- \| --- \| --- \| --- \| --- \| --- \| --- \| --- \| --- \| --- \| --- \| --- \| --- \| --- \| --- \| --- \| --- \| \|  \|  \|  \|  \|  \|  \|  \|  \|  \|  \|  \|  \|  \|  \|  \|  \|  \|  \|   Please indicate if this made you feel helpless or terrified. | 🔿  Yes_1_  ❒  Helpless | 🔿  No_0_  ❒  Terrified |  |
| 25. | Threatened to harm your sibling (stepsibling).  Please check all ages that apply.   \| 1 \| 2 \| 3 \| 4 \| 5 \| 6 \| 7 \| 8 \| 9 \| 10 \| 11 \| 12 \| 13 \| 14 \| 15 \| 16 \| 17 \| 18 \| \| --- \| --- \| --- \| --- \| --- \| --- \| --- \| --- \| --- \| --- \| --- \| --- \| --- \| --- \| --- \| --- \| --- \| --- \| \|  \|  \|  \|  \|  \|  \|  \|  \|  \|  \|  \|  \|  \|  \|  \|  \|  \|  \|   Please indicate if this made you feel helpless or terrified. | 🔿  Yes_1_  ❒  Helpless | 🔿  No_0_  ❒  Terrified |  |

|  | **Sometimes adults or older individuals NOT living in the house do hurtful things to you.**  **If this happened during your childhood (first 18 years of your life) please provide your best estimates of your age at the time(s) of occurrence.**  **Please check all ages that apply.** | | |  | |  | |
| --- | --- | --- | --- | --- | --- | --- | --- |
| 26. | | Made inappropriate sexual comments or suggestions to you.  Please check all ages that apply.   \| 1 \| 2 \| 3 \| 4 \| 5 \| 6 \| 7 \| 8 \| 9 \| 10 \| 11 \| 12 \| 13 \| 14 \| 15 \| 16 \| 17 \| 18 \| \| --- \| --- \| --- \| --- \| --- \| --- \| --- \| --- \| --- \| --- \| --- \| --- \| --- \| --- \| --- \| --- \| --- \| --- \| \|  \|  \|  \|  \|  \|  \|  \|  \|  \|  \|  \|  \|  \|  \|  \|  \|  \|  \|   Please indicate if this made you feel helpless or terrified. | 🔿  Yes_1_  ❒  Helpless | | 🔿  No_0_  ❒  Terrified | |  |
| 27. | | Touched or fondled your body in a sexual way.  Please check all ages that apply.   \| 1 \| 2 \| 3 \| 4 \| 5 \| 6 \| 7 \| 8 \| 9 \| 10 \| 11 \| 12 \| 13 \| 14 \| 15 \| 16 \| 17 \| 18 \| \| --- \| --- \| --- \| --- \| --- \| --- \| --- \| --- \| --- \| --- \| --- \| --- \| --- \| --- \| --- \| --- \| --- \| --- \| \|  \|  \|  \|  \|  \|  \|  \|  \|  \|  \|  \|  \|  \|  \|  \|  \|  \|  \|   Please indicate if this made you feel helpless or terrified. | 🔿  Yes_1_  ❒  Helpless | | 🔿  No_0_  ❒  Terrified | |  |
| 28. | | Had you touch their body in a sexual way.  Please check all ages that apply.   \| 1 \| 2 \| 3 \| 4 \| 5 \| 6 \| 7 \| 8 \| 9 \| 10 \| 11 \| 12 \| 13 \| 14 \| 15 \| 16 \| 17 \| 18 \| \| --- \| --- \| --- \| --- \| --- \| --- \| --- \| --- \| --- \| --- \| --- \| --- \| --- \| --- \| --- \| --- \| --- \| --- \| \|  \|  \|  \|  \|  \|  \|  \|  \|  \|  \|  \|  \|  \|  \|  \|  \|  \|  \|   Please indicate if this made you feel helpless or terrified. | 🔿  Yes_1_  ❒  Helpless | | 🔿  No_0_  ❒  Terrified | |  |
| 29. | | Attempted to have any type of sexual intercourse (oral, anal or vaginal) with you.  Please check all ages that apply.   \| 1 \| 2 \| 3 \| 4 \| 5 \| 6 \| 7 \| 8 \| 9 \| 10 \| 11 \| 12 \| 13 \| 14 \| 15 \| 16 \| 17 \| 18 \| \| --- \| --- \| --- \| --- \| --- \| --- \| --- \| --- \| --- \| --- \| --- \| --- \| --- \| --- \| --- \| --- \| --- \| --- \| \|  \|  \|  \|  \|  \|  \|  \|  \|  \|  \|  \|  \|  \|  \|  \|  \|  \|  \|   Please indicate if this made you feel helpless or terrified. | 🔿  Yes_1_  ❒  Helpless | | 🔿  No_0_  ❒  Terrified | |  |
| 30. | | Actually had sexual intercourse (oral, anal or vaginal) with you.  Please check all ages that apply.   \| 1 \| 2 \| 3 \| 4 \| 5 \| 6 \| 7 \| 8 \| 9 \| 10 \| 11 \| 12 \| 13 \| 14 \| 15 \| 16 \| 17 \| 18 \| \| --- \| --- \| --- \| --- \| --- \| --- \| --- \| --- \| --- \| --- \| --- \| --- \| --- \| --- \| --- \| --- \| --- \| --- \| \|  \|  \|  \|  \|  \|  \|  \|  \|  \|  \|  \|  \|  \|  \|  \|  \|  \|  \|   Please indicate if this made you feel helpless or terrified. | 🔿  Yes_1_  ❒  Helpless | | 🔿  No_0_  ❒  Terrified | |  |

|  | **Sometimes intense arguments or physical fights occur between parents, stepparents or other adults (boyfriends, girlfriends, grandparents) living in the household.**  **If this happened during your childhood (first 18 years of your life) please provide your best estimates of your age at the time(s) of occurrence.**  **Please check all ages that apply.** | | |  | |  | |
| --- | --- | --- | --- | --- | --- | --- | --- |
| 31. | | Witnessed adults living in the household argue intensely with your mother (stepmother, grandmother), say derogatory things to her, or threaten her with harm.  Please check all ages that apply.   \| 1 \| 2 \| 3 \| 4 \| 5 \| 6 \| 7 \| 8 \| 9 \| 10 \| 11 \| 12 \| 13 \| 14 \| 15 \| 16 \| 17 \| 18 \| \| --- \| --- \| --- \| --- \| --- \| --- \| --- \| --- \| --- \| --- \| --- \| --- \| --- \| --- \| --- \| --- \| --- \| --- \| \|  \|  \|  \|  \|  \|  \|  \|  \|  \|  \|  \|  \|  \|  \|  \|  \|  \|  \|   Please indicate if this made you feel helpless or terrified. | 🔿  Yes_1_  ❒  Helpless | | 🔿  No_0_  ❒  Terrified | |  |
| 32. | | Witnessed adults living in the household argue intensely with your father (stepfather, grandfather), say derogatory things to him, or threaten him with harm.  Please check all ages that apply.   \| 1 \| 2 \| 3 \| 4 \| 5 \| 6 \| 7 \| 8 \| 9 \| 10 \| 11 \| 12 \| 13 \| 14 \| 15 \| 16 \| 17 \| 18 \| \| --- \| --- \| --- \| --- \| --- \| --- \| --- \| --- \| --- \| --- \| --- \| --- \| --- \| --- \| --- \| --- \| --- \| --- \| \|  \|  \|  \|  \|  \|  \|  \|  \|  \|  \|  \|  \|  \|  \|  \|  \|  \|  \|   Please indicate if this made you feel helpless or terrified. | 🔿  Yes_1_  ❒  Helpless | | 🔿  No_0_  ❒  Terrified | |  |
| 33. | | Saw adults living in the household push, grab, slap or throw something at your mother (stepmother, grandmother).  Please check all ages that apply.   \| 1 \| 2 \| 3 \| 4 \| 5 \| 6 \| 7 \| 8 \| 9 \| 10 \| 11 \| 12 \| 13 \| 14 \| 15 \| 16 \| 17 \| 18 \| \| --- \| --- \| --- \| --- \| --- \| --- \| --- \| --- \| --- \| --- \| --- \| --- \| --- \| --- \| --- \| --- \| --- \| --- \| \|  \|  \|  \|  \|  \|  \|  \|  \|  \|  \|  \|  \|  \|  \|  \|  \|  \|  \|   Please indicate if this made you feel helpless or terrified. | 🔿  Yes_1_  ❒  Helpless | | 🔿  No_0_  ❒  Terrified | |  |
| 34. | | Saw adults living in the household hit your mother (stepmother, grandmother) so hard that it left marks for more than a few minutes.  Please check all ages that apply.   \| 1 \| 2 \| 3 \| 4 \| 5 \| 6 \| 7 \| 8 \| 9 \| 10 \| 11 \| 12 \| 13 \| 14 \| 15 \| 16 \| 17 \| 18 \| \| --- \| --- \| --- \| --- \| --- \| --- \| --- \| --- \| --- \| --- \| --- \| --- \| --- \| --- \| --- \| --- \| --- \| --- \| \|  \|  \|  \|  \|  \|  \|  \|  \|  \|  \|  \|  \|  \|  \|  \|  \|  \|  \|   Please indicate if this made you feel helpless or terrified. | 🔿  Yes_1_  ❒  Helpless | | 🔿  No_0_  ❒  Terrified | |  |
| 35. | | Saw adults living in the household hit your mother (stepmother, grandmother) so hard, or intentionally harm her in some way, that she received or should have received medical attention.  Please check all ages that apply.   \| 1 \| 2 \| 3 \| 4 \| 5 \| 6 \| 7 \| 8 \| 9 \| 10 \| 11 \| 12 \| 13 \| 14 \| 15 \| 16 \| 17 \| 18 \| \| --- \| --- \| --- \| --- \| --- \| --- \| --- \| --- \| --- \| --- \| --- \| --- \| --- \| --- \| --- \| --- \| --- \| --- \| \|  \|  \|  \|  \|  \|  \|  \|  \|  \|  \|  \|  \|  \|  \|  \|  \|  \|  \|   Please indicate if this made you feel helpless or terrified. | 🔿  Yes_1_  ❒  Helpless | | 🔿  No_0_  ❒  Terrified | |  |
| 36. | | Saw adults living in the household push, grab, slap or throw something at your father (stepfather, grandfather).  Please check all ages that apply.   \| 1 \| 2 \| 3 \| 4 \| 5 \| 6 \| 7 \| 8 \| 9 \| 10 \| 11 \| 12 \| 13 \| 14 \| 15 \| 16 \| 17 \| 18 \| \| --- \| --- \| --- \| --- \| --- \| --- \| --- \| --- \| --- \| --- \| --- \| --- \| --- \| --- \| --- \| --- \| --- \| --- \| \|  \|  \|  \|  \|  \|  \|  \|  \|  \|  \|  \|  \|  \|  \|  \|  \|  \|  \|   Please indicate if this made you feel helpless or terrified. | 🔿  Yes_1_  ❒  Helpless | | 🔿  No_0_  ❒  Terrified | |  |
| 37. | | Saw adults living in the household hit your father (stepfather, grandfather) so hard that it left marks for more than a few minutes.  Please check all ages that apply.   \| 1 \| 2 \| 3 \| 4 \| 5 \| 6 \| 7 \| 8 \| 9 \| 10 \| 11 \| 12 \| 13 \| 14 \| 15 \| 16 \| 17 \| 18 \| \| --- \| --- \| --- \| --- \| --- \| --- \| --- \| --- \| --- \| --- \| --- \| --- \| --- \| --- \| --- \| --- \| --- \| --- \| \|  \|  \|  \|  \|  \|  \|  \|  \|  \|  \|  \|  \|  \|  \|  \|  \|  \|  \|   Please indicate if this made you feel helpless or terrified. | 🔿  Yes_1_  ❒  Helpless | | 🔿  No_0_  ❒  Terrified | |  |
| 38. | | Saw adults living in the household hit your father (stepfather, grandfather) so hard, or intentionally harm him in some way, that he received or should have received medical attention.  Please check all ages that apply.   \| 1 \| 2 \| 3 \| 4 \| 5 \| 6 \| 7 \| 8 \| 9 \| 10 \| 11 \| 12 \| 13 \| 14 \| 15 \| 16 \| 17 \| 18 \| \| --- \| --- \| --- \| --- \| --- \| --- \| --- \| --- \| --- \| --- \| --- \| --- \| --- \| --- \| --- \| --- \| --- \| --- \| \|  \|  \|  \|  \|  \|  \|  \|  \|  \|  \|  \|  \|  \|  \|  \|  \|  \|  \|   Please indicate if this made you feel helpless or terrified. | 🔿  Yes_1_  ❒  Helpless | | 🔿  No_0_  ❒  Terrified | |  |

|  | **Sometimes children your own age or older do hurtful things like bully or harass you.**  **If this happened during your childhood (first 18 years of your life) please provide your best estimates of your age at the time(s) of occurrence.**  **Please check all ages that apply.** | | |  | |  | |
| --- | --- | --- | --- | --- | --- | --- | --- |
| 39. | | Swore at you, called you names, said insulting things like your “fat”, “ugly”, “stupid”, etc. more than a few times a year.  Please check all ages that apply.   \| 1 \| 2 \| 3 \| 4 \| 5 \| 6 \| 7 \| 8 \| 9 \| 10 \| 11 \| 12 \| 13 \| 14 \| 15 \| 16 \| 17 \| 18 \| \| --- \| --- \| --- \| --- \| --- \| --- \| --- \| --- \| --- \| --- \| --- \| --- \| --- \| --- \| --- \| --- \| --- \| --- \| \|  \|  \|  \|  \|  \|  \|  \|  \|  \|  \|  \|  \|  \|  \|  \|  \|  \|  \|   Please indicate ages when (if) the person doing this to you was a date (e.g., boyfriend, girlfriend, someone you associated with on a social, romantic or intimate level).   \| 1 \| 2 \| 3 \| 4 \| 5 \| 6 \| 7 \| 8 \| 9 \| 10 \| 11 \| 12 \| 13 \| 14 \| 15 \| 16 \| 17 \| 18 \| \| --- \| --- \| --- \| --- \| --- \| --- \| --- \| --- \| --- \| --- \| --- \| --- \| --- \| --- \| --- \| --- \| --- \| --- \| \|  \|  \|  \|  \|  \|  \|  \|  \|  \|  \|  \|  \|  \|  \|  \|  \|  \|  \| | 🔿  Yes_1_  🔿  Yes_1_ | | 🔿  No_0_  🔿  No_0_ | |  |
| 40. | | Said hurtful things that made you feel bad, embarrassed or humiliated more than a few times a year.  Please check all ages that apply.   \| 1 \| 2 \| 3 \| 4 \| 5 \| 6 \| 7 \| 8 \| 9 \| 10 \| 11 \| 12 \| 13 \| 14 \| 15 \| 16 \| 17 \| 18 \| \| --- \| --- \| --- \| --- \| --- \| --- \| --- \| --- \| --- \| --- \| --- \| --- \| --- \| --- \| --- \| --- \| --- \| --- \| \|  \|  \|  \|  \|  \|  \|  \|  \|  \|  \|  \|  \|  \|  \|  \|  \|  \|  \|   Please indicate ages when (if) the person doing this to you was a date.   \| 1 \| 2 \| 3 \| 4 \| 5 \| 6 \| 7 \| 8 \| 9 \| 10 \| 11 \| 12 \| 13 \| 14 \| 15 \| 16 \| 17 \| 18 \| \| --- \| --- \| --- \| --- \| --- \| --- \| --- \| --- \| --- \| --- \| --- \| --- \| --- \| --- \| --- \| --- \| --- \| --- \| \|  \|  \|  \|  \|  \|  \|  \|  \|  \|  \|  \|  \|  \|  \|  \|  \|  \|  \| | 🔿  Yes_1_  🔿  Yes_1_ | | 🔿  No_0_  🔿  No_0_ | |  |

| 41. | Said things behind your back, posted derogatory messages about you, or spread rumors about you.  Please check all ages that apply.   \| 1 \| 2 \| 3 \| 4 \| 5 \| 6 \| 7 \| 8 \| 9 \| 10 \| 11 \| 12 \| 13 \| 14 \| 15 \| 16 \| 17 \| 18 \| \| --- \| --- \| --- \| --- \| --- \| --- \| --- \| --- \| --- \| --- \| --- \| --- \| --- \| --- \| --- \| --- \| --- \| --- \| \|  \|  \|  \|  \|  \|  \|  \|  \|  \|  \|  \|  \|  \|  \|  \|  \|  \|  \|   Please indicate ages when (if) the person doing this to you was a date.   \| 1 \| 2 \| 3 \| 4 \| 5 \| 6 \| 7 \| 8 \| 9 \| 10 \| 11 \| 12 \| 13 \| 14 \| 15 \| 16 \| 17 \| 18 \| \| --- \| --- \| --- \| --- \| --- \| --- \| --- \| --- \| --- \| --- \| --- \| --- \| --- \| --- \| --- \| --- \| --- \| --- \| \|  \|  \|  \|  \|  \|  \|  \|  \|  \|  \|  \|  \|  \|  \|  \|  \|  \|  \| | 🔿  Yes_1_  🔿  Yes_1_ | 🔿  No_0_  🔿  No_0_ |  |
| --- | --- | --- | --- | --- | --- | --- | --- | --- | --- | --- | --- | --- | --- | --- | --- | --- | --- | --- | --- | --- | --- | --- | --- | --- | --- | --- | --- | --- | --- | --- | --- | --- | --- | --- | --- | --- | --- | --- | --- | --- | --- | --- | --- | --- | --- | --- | --- | --- | --- | --- | --- | --- | --- | --- | --- | --- | --- | --- | --- | --- | --- | --- | --- | --- | --- | --- | --- | --- | --- | --- | --- | --- | --- | --- | --- | --- |
| 42. | Intentionally excluded you from activities or groups.  Please check all ages that apply.   \| 1 \| 2 \| 3 \| 4 \| 5 \| 6 \| 7 \| 8 \| 9 \| 10 \| 11 \| 12 \| 13 \| 14 \| 15 \| 16 \| 17 \| 18 \| \| --- \| --- \| --- \| --- \| --- \| --- \| --- \| --- \| --- \| --- \| --- \| --- \| --- \| --- \| --- \| --- \| --- \| --- \| \|  \|  \|  \|  \|  \|  \|  \|  \|  \|  \|  \|  \|  \|  \|  \|  \|  \|  \|   Please indicate ages when (if) the person doing this to you was a date.   \| 1 \| 2 \| 3 \| 4 \| 5 \| 6 \| 7 \| 8 \| 9 \| 10 \| 11 \| 12 \| 13 \| 14 \| 15 \| 16 \| 17 \| 18 \| \| --- \| --- \| --- \| --- \| --- \| --- \| --- \| --- \| --- \| --- \| --- \| --- \| --- \| --- \| --- \| --- \| --- \| --- \| \|  \|  \|  \|  \|  \|  \|  \|  \|  \|  \|  \|  \|  \|  \|  \|  \|  \|  \| | 🔿  Yes_1_  🔿  Yes_1_ | 🔿  No_0_  🔿  No_0_ |  |
| 43. | Acted in a way that made you afraid that you might be physically hurt.  Please check all ages that apply.   \| 1 \| 2 \| 3 \| 4 \| 5 \| 6 \| 7 \| 8 \| 9 \| 10 \| 11 \| 12 \| 13 \| 14 \| 15 \| 16 \| 17 \| 18 \| \| --- \| --- \| --- \| --- \| --- \| --- \| --- \| --- \| --- \| --- \| --- \| --- \| --- \| --- \| --- \| --- \| --- \| --- \| \|  \|  \|  \|  \|  \|  \|  \|  \|  \|  \|  \|  \|  \|  \|  \|  \|  \|  \|   Please indicate ages when (if) the person doing this to you was a date.   \| 1 \| 2 \| 3 \| 4 \| 5 \| 6 \| 7 \| 8 \| 9 \| 10 \| 11 \| 12 \| 13 \| 14 \| 15 \| 16 \| 17 \| 18 \| \| --- \| --- \| --- \| --- \| --- \| --- \| --- \| --- \| --- \| --- \| --- \| --- \| --- \| --- \| --- \| --- \| --- \| --- \| \|  \|  \|  \|  \|  \|  \|  \|  \|  \|  \|  \|  \|  \|  \|  \|  \|  \|  \| | 🔿  Yes_1_  🔿  Yes_1_ | 🔿  No_0_  🔿  No_0_ |  |
| 44. | Threatened you in order to take your money or possessions.  Please check all ages that apply.   \| 1 \| 2 \| 3 \| 4 \| 5 \| 6 \| 7 \| 8 \| 9 \| 10 \| 11 \| 12 \| 13 \| 14 \| 15 \| 16 \| 17 \| 18 \| \| --- \| --- \| --- \| --- \| --- \| --- \| --- \| --- \| --- \| --- \| --- \| --- \| --- \| --- \| --- \| --- \| --- \| --- \| \|  \|  \|  \|  \|  \|  \|  \|  \|  \|  \|  \|  \|  \|  \|  \|  \|  \|  \|   Please indicate ages when (if) the person doing this to you was a date.   \| 1 \| 2 \| 3 \| 4 \| 5 \| 6 \| 7 \| 8 \| 9 \| 10 \| 11 \| 12 \| 13 \| 14 \| 15 \| 16 \| 17 \| 18 \| \| --- \| --- \| --- \| --- \| --- \| --- \| --- \| --- \| --- \| --- \| --- \| --- \| --- \| --- \| --- \| --- \| --- \| --- \| \|  \|  \|  \|  \|  \|  \|  \|  \|  \|  \|  \|  \|  \|  \|  \|  \|  \|  \| | 🔿  Yes_1_  🔿  Yes_1_ | 🔿  No_0_  🔿  No_0_ |  |
| 45. | Forced or threatened you to do things that you did not want to do.  Please check all ages that apply.   \| 1 \| 2 \| 3 \| 4 \| 5 \| 6 \| 7 \| 8 \| 9 \| 10 \| 11 \| 12 \| 13 \| 14 \| 15 \| 16 \| 17 \| 18 \| \| --- \| --- \| --- \| --- \| --- \| --- \| --- \| --- \| --- \| --- \| --- \| --- \| --- \| --- \| --- \| --- \| --- \| --- \| \|  \|  \|  \|  \|  \|  \|  \|  \|  \|  \|  \|  \|  \|  \|  \|  \|  \|  \|   If yes, please describe examples:   \|  \| \| --- \|   Please indicate ages when (if) the person doing this to you was a date.   \| 1 \| 2 \| 3 \| 4 \| 5 \| 6 \| 7 \| 8 \| 9 \| 10 \| 11 \| 12 \| 13 \| 14 \| 15 \| 16 \| 17 \| 18 \| \| --- \| --- \| --- \| --- \| --- \| --- \| --- \| --- \| --- \| --- \| --- \| --- \| --- \| --- \| --- \| --- \| --- \| --- \| \|  \|  \|  \|  \|  \|  \|  \|  \|  \|  \|  \|  \|  \|  \|  \|  \|  \|  \| | 🔿  Yes_1_  🔿  Yes_1_ | 🔿  No_0_  🔿  No_0_ |  |

| 46. | Intentionally pushed, grabbed, shoved, slapped, pinched, punched, or kicked you.  Please check all ages that apply.   \| 1 \| 2 \| 3 \| 4 \| 5 \| 6 \| 7 \| 8 \| 9 \| 10 \| 11 \| 12 \| 13 \| 14 \| 15 \| 16 \| 17 \| 18 \| \| --- \| --- \| --- \| --- \| --- \| --- \| --- \| --- \| --- \| --- \| --- \| --- \| --- \| --- \| --- \| --- \| --- \| --- \| \|  \|  \|  \|  \|  \|  \|  \|  \|  \|  \|  \|  \|  \|  \|  \|  \|  \|  \|   Please indicate ages when (if) the person doing this to you was a date.   \| 1 \| 2 \| 3 \| 4 \| 5 \| 6 \| 7 \| 8 \| 9 \| 10 \| 11 \| 12 \| 13 \| 14 \| 15 \| 16 \| 17 \| 18 \| \| --- \| --- \| --- \| --- \| --- \| --- \| --- \| --- \| --- \| --- \| --- \| --- \| --- \| --- \| --- \| --- \| --- \| --- \| \|  \|  \|  \|  \|  \|  \|  \|  \|  \|  \|  \|  \|  \|  \|  \|  \|  \|  \| | 🔿  Yes_1_  🔿  Yes_1_ | 🔿  No_0_  🔿  No_0_ |  |
| --- | --- | --- | --- | --- | --- | --- | --- | --- | --- | --- | --- | --- | --- | --- | --- | --- | --- | --- | --- | --- | --- | --- | --- | --- | --- | --- | --- | --- | --- | --- | --- | --- | --- | --- | --- | --- | --- | --- | --- | --- | --- | --- | --- | --- | --- | --- | --- | --- | --- | --- | --- | --- | --- | --- | --- | --- | --- | --- | --- | --- | --- | --- | --- | --- | --- | --- | --- | --- | --- | --- | --- | --- | --- | --- | --- | --- |
| 47. | Hit you so hard that it left marks for more than a few minutes.  Please check all ages that apply.   \| 1 \| 2 \| 3 \| 4 \| 5 \| 6 \| 7 \| 8 \| 9 \| 10 \| 11 \| 12 \| 13 \| 14 \| 15 \| 16 \| 17 \| 18 \| \| --- \| --- \| --- \| --- \| --- \| --- \| --- \| --- \| --- \| --- \| --- \| --- \| --- \| --- \| --- \| --- \| --- \| --- \| \|  \|  \|  \|  \|  \|  \|  \|  \|  \|  \|  \|  \|  \|  \|  \|  \|  \|  \|   Please indicate ages when (if) the person doing this to you was a date.   \| 1 \| 2 \| 3 \| 4 \| 5 \| 6 \| 7 \| 8 \| 9 \| 10 \| 11 \| 12 \| 13 \| 14 \| 15 \| 16 \| 17 \| 18 \| \| --- \| --- \| --- \| --- \| --- \| --- \| --- \| --- \| --- \| --- \| --- \| --- \| --- \| --- \| --- \| --- \| --- \| --- \| \|  \|  \|  \|  \|  \|  \|  \|  \|  \|  \|  \|  \|  \|  \|  \|  \|  \|  \| | 🔿  Yes_1_  🔿  Yes_1_ | 🔿  No_0_  🔿  No_0_ |  |
| 48. | Hit you so hard, or intentionally harmed you in some way, that you received or should have received medical attention.  Please check all ages that apply.   \| 1 \| 2 \| 3 \| 4 \| 5 \| 6 \| 7 \| 8 \| 9 \| 10 \| 11 \| 12 \| 13 \| 14 \| 15 \| 16 \| 17 \| 18 \| \| --- \| --- \| --- \| --- \| --- \| --- \| --- \| --- \| --- \| --- \| --- \| --- \| --- \| --- \| --- \| --- \| --- \| --- \| \|  \|  \|  \|  \|  \|  \|  \|  \|  \|  \|  \|  \|  \|  \|  \|  \|  \|  \|   Please indicate ages when (if) the person doing this to you was a date.   \| 1 \| 2 \| 3 \| 4 \| 5 \| 6 \| 7 \| 8 \| 9 \| 10 \| 11 \| 12 \| 13 \| 14 \| 15 \| 16 \| 17 \| 18 \| \| --- \| --- \| --- \| --- \| --- \| --- \| --- \| --- \| --- \| --- \| --- \| --- \| --- \| --- \| --- \| --- \| --- \| --- \| \|  \|  \|  \|  \|  \|  \|  \|  \|  \|  \|  \|  \|  \|  \|  \|  \|  \|  \| | 🔿  Yes_1_  🔿  Yes_1_ | 🔿  No_0_  🔿  No_0_ |  |
| 49. | Forced you to engage in sexual activity against your will.  Please check all ages that apply.   \| 1 \| 2 \| 3 \| 4 \| 5 \| 6 \| 7 \| 8 \| 9 \| 10 \| 11 \| 12 \| 13 \| 14 \| 15 \| 16 \| 17 \| 18 \| \| --- \| --- \| --- \| --- \| --- \| --- \| --- \| --- \| --- \| --- \| --- \| --- \| --- \| --- \| --- \| --- \| --- \| --- \| \|  \|  \|  \|  \|  \|  \|  \|  \|  \|  \|  \|  \|  \|  \|  \|  \|  \|  \|   Please indicate ages when (if) the person doing this to you was a date.   \| 1 \| 2 \| 3 \| 4 \| 5 \| 6 \| 7 \| 8 \| 9 \| 10 \| 11 \| 12 \| 13 \| 14 \| 15 \| 16 \| 17 \| 18 \| \| --- \| --- \| --- \| --- \| --- \| --- \| --- \| --- \| --- \| --- \| --- \| --- \| --- \| --- \| --- \| --- \| --- \| --- \| \|  \|  \|  \|  \|  \|  \|  \|  \|  \|  \|  \|  \|  \|  \|  \|  \|  \|  \| | 🔿  Yes_1_  🔿  Yes_1_ | 🔿  No_0_  🔿  No_0_ |  |
| 50. | Forced you to do things sexually that you did not want to do.  Please check all ages that apply.   \| 1 \| 2 \| 3 \| 4 \| 5 \| 6 \| 7 \| 8 \| 9 \| 10 \| 11 \| 12 \| 13 \| 14 \| 15 \| 16 \| 17 \| 18 \| \| --- \| --- \| --- \| --- \| --- \| --- \| --- \| --- \| --- \| --- \| --- \| --- \| --- \| --- \| --- \| --- \| --- \| --- \| \|  \|  \|  \|  \|  \|  \|  \|  \|  \|  \|  \|  \|  \|  \|  \|  \|  \|  \|   Please indicate ages when (if) the person doing this to you was a date.   \| 1 \| 2 \| 3 \| 4 \| 5 \| 6 \| 7 \| 8 \| 9 \| 10 \| 11 \| 12 \| 13 \| 14 \| 15 \| 16 \| 17 \| 18 \| \| --- \| --- \| --- \| --- \| --- \| --- \| --- \| --- \| --- \| --- \| --- \| --- \| --- \| --- \| --- \| --- \| --- \| --- \| \|  \|  \|  \|  \|  \|  \|  \|  \|  \|  \|  \|  \|  \|  \|  \|  \|  \|  \| | 🔿  Yes_1_  🔿  Yes_1_ | 🔿  No_0_  🔿  No_0_ |  |

|  | Please indicate if the following happened during your childhood (first 18 years of your life). Please provide your best estimates of your age at the time(s) of occurrence.  **Please check all ages that apply.** | | |  | |  | |
| --- | --- | --- | --- | --- | --- | --- | --- |
| 51. | | You felt that your mother or other important maternal figure was present in the household but emotionally unavailable to you for a variety of reasons like drugs, alcohol, workaholic, having an affair, heedlessly pursuing their own goals.  Please check all ages that apply.   \| 1 \| 2 \| 3 \| 4 \| 5 \| 6 \| 7 \| 8 \| 9 \| 10 \| 11 \| 12 \| 13 \| 14 \| 15 \| 16 \| 17 \| 18 \| \| --- \| --- \| --- \| --- \| --- \| --- \| --- \| --- \| --- \| --- \| --- \| --- \| --- \| --- \| --- \| --- \| --- \| --- \| \|  \|  \|  \|  \|  \|  \|  \|  \|  \|  \|  \|  \|  \|  \|  \|  \|  \|  \| | 🔿  Yes_1_ | | 🔿  No_0_ | |  |
| 52. | | You felt that your father or other important paternal figure was present in the household but emotionally unavailable to you for a variety of reasons like drugs, alcohol, workaholic, having an affair, heedlessly pursuing their own goals.  Please check all ages that apply.   \| 1 \| 2 \| 3 \| 4 \| 5 \| 6 \| 7 \| 8 \| 9 \| 10 \| 11 \| 12 \| 13 \| 14 \| 15 \| 16 \| 17 \| 18 \| \| --- \| --- \| --- \| --- \| --- \| --- \| --- \| --- \| --- \| --- \| --- \| --- \| --- \| --- \| --- \| --- \| --- \| --- \| \|  \|  \|  \|  \|  \|  \|  \|  \|  \|  \|  \|  \|  \|  \|  \|  \|  \|  \| | 🔿  Yes_1_ | | 🔿  No_0_ | |  |
| 53. | | You felt that your mother or other important maternal figure was emotionally unavailable to you for a variety of reasons like military service, taking care of a sick relative, in school, business necessity.  Please check all ages that apply.   \| 1 \| 2 \| 3 \| 4 \| 5 \| 6 \| 7 \| 8 \| 9 \| 10 \| 11 \| 12 \| 13 \| 14 \| 15 \| 16 \| 17 \| 18 \| \| --- \| --- \| --- \| --- \| --- \| --- \| --- \| --- \| --- \| --- \| --- \| --- \| --- \| --- \| --- \| --- \| --- \| --- \| \|  \|  \|  \|  \|  \|  \|  \|  \|  \|  \|  \|  \|  \|  \|  \|  \|  \|  \| | 🔿  Yes_1_ | | 🔿  No_0_ | |  |
| 54. | | You felt that your father or other important paternal figure was emotionally unavailable to you for a variety of reasons like military service, taking care of a sick relative, in school, business necessity.  Please check all ages that apply.   \| 1 \| 2 \| 3 \| 4 \| 5 \| 6 \| 7 \| 8 \| 9 \| 10 \| 11 \| 12 \| 13 \| 14 \| 15 \| 16 \| 17 \| 18 \| \| --- \| --- \| --- \| --- \| --- \| --- \| --- \| --- \| --- \| --- \| --- \| --- \| --- \| --- \| --- \| --- \| --- \| --- \| \|  \|  \|  \|  \|  \|  \|  \|  \|  \|  \|  \|  \|  \|  \|  \|  \|  \|  \| | 🔿  Yes_1_ | | 🔿  No_0_ | |  |
| 55. | | A parent or other important parental figure was very difficult to please.  Please check all ages that apply.   \| 1 \| 2 \| 3 \| 4 \| 5 \| 6 \| 7 \| 8 \| 9 \| 10 \| 11 \| 12 \| 13 \| 14 \| 15 \| 16 \| 17 \| 18 \| \| --- \| --- \| --- \| --- \| --- \| --- \| --- \| --- \| --- \| --- \| --- \| --- \| --- \| --- \| --- \| --- \| --- \| --- \| \|  \|  \|  \|  \|  \|  \|  \|  \|  \|  \|  \|  \|  \|  \|  \|  \|  \|  \| | 🔿  Yes_1_ | | 🔿  No_0_ | |  |
| 56. | | A parent or other important parental figure did not have the time or interest to talk to you.  Please check all ages that apply.   \| 1 \| 2 \| 3 \| 4 \| 5 \| 6 \| 7 \| 8 \| 9 \| 10 \| 11 \| 12 \| 13 \| 14 \| 15 \| 16 \| 17 \| 18 \| \| --- \| --- \| --- \| --- \| --- \| --- \| --- \| --- \| --- \| --- \| --- \| --- \| --- \| --- \| --- \| --- \| --- \| --- \| \|  \|  \|  \|  \|  \|  \|  \|  \|  \|  \|  \|  \|  \|  \|  \|  \|  \|  \| | 🔿  Yes_1_ | | 🔿  No_0_ | |  |
| 57. | | One or more individuals in your family made you feel loved.  Please check all ages that apply.   \| 1 \| 2 \| 3 \| 4 \| 5 \| 6 \| 7 \| 8 \| 9 \| 10 \| 11 \| 12 \| 13 \| 14 \| 15 \| 16 \| 17 \| 18 \| \| --- \| --- \| --- \| --- \| --- \| --- \| --- \| --- \| --- \| --- \| --- \| --- \| --- \| --- \| --- \| --- \| --- \| --- \| \|  \|  \|  \|  \|  \|  \|  \|  \|  \|  \|  \|  \|  \|  \|  \|  \|  \|  \|   Who? (e.g. mother, aunt, maternal grandfather)   \|  \| \| --- \| | 🔿  Yes_1_ | | 🔿  No_0_ | |  |

| 58. | One or more individuals in your family helped you feel important or special.  Please check all ages that apply.   \| 1 \| 2 \| 3 \| 4 \| 5 \| 6 \| 7 \| 8 \| 9 \| 10 \| 11 \| 12 \| 13 \| 14 \| 15 \| 16 \| 17 \| 18 \| \| --- \| --- \| --- \| --- \| --- \| --- \| --- \| --- \| --- \| --- \| --- \| --- \| --- \| --- \| --- \| --- \| --- \| --- \| \|  \|  \|  \|  \|  \|  \|  \|  \|  \|  \|  \|  \|  \|  \|  \|  \|  \|  \|   Who? (e.g. mother, aunt, maternal grandfather)   \|  \| \| --- \| | 🔿  Yes_1_ | 🔿  No_0_ |  |
| --- | --- | --- | --- | --- | --- | --- | --- | --- | --- | --- | --- | --- | --- | --- | --- | --- | --- | --- | --- | --- | --- | --- | --- | --- | --- | --- | --- | --- | --- | --- | --- | --- | --- | --- | --- | --- | --- | --- | --- | --- | --- |
| 59. | One or more individuals in your family were there to take care of you and protect you.  Please check all ages that apply.   \| 1 \| 2 \| 3 \| 4 \| 5 \| 6 \| 7 \| 8 \| 9 \| 10 \| 11 \| 12 \| 13 \| 14 \| 15 \| 16 \| 17 \| 18 \| \| --- \| --- \| --- \| --- \| --- \| --- \| --- \| --- \| --- \| --- \| --- \| --- \| --- \| --- \| --- \| --- \| --- \| --- \| \|  \|  \|  \|  \|  \|  \|  \|  \|  \|  \|  \|  \|  \|  \|  \|  \|  \|  \|   Who? (e.g. mother, aunt, maternal grandfather)   \|  \| \| --- \| | 🔿  Yes_1_ | 🔿  No_0_ |  |
| 60. | One or more individuals in your family were there to take you to the doctor or Emergency Room if the need ever arose.  Please check all ages that apply.   \| 1 \| 2 \| 3 \| 4 \| 5 \| 6 \| 7 \| 8 \| 9 \| 10 \| 11 \| 12 \| 13 \| 14 \| 15 \| 16 \| 17 \| 18 \| \| --- \| --- \| --- \| --- \| --- \| --- \| --- \| --- \| --- \| --- \| --- \| --- \| --- \| --- \| --- \| --- \| --- \| --- \| \|  \|  \|  \|  \|  \|  \|  \|  \|  \|  \|  \|  \|  \|  \|  \|  \|  \|  \|   Who? (e.g. mother, aunt, maternal grandfather)   \|  \| \| --- \| | 🔿  Yes_1_ | 🔿  No_0_ |  |
| 61. | One or more individuals in your family would help you with your homework, or to get ready for school.  Please check all ages that apply.   \| 1 \| 2 \| 3 \| 4 \| 5 \| 6 \| 7 \| 8 \| 9 \| 10 \| 11 \| 12 \| 13 \| 14 \| 15 \| 16 \| 17 \| 18 \| \| --- \| --- \| --- \| --- \| --- \| --- \| --- \| --- \| --- \| --- \| --- \| --- \| --- \| --- \| --- \| --- \| --- \| --- \| \|  \|  \|  \|  \|  \|  \|  \|  \|  \|  \|  \|  \|  \|  \|  \|  \|  \|  \| | 🔿  Yes_1_ | 🔿  No_0_ |  |

|  | **Please indicate if the following statements were true about you and your family during your childhood, and your age at the time(s) you felt this to be true.**  **Please check all ages that apply.** | | |  | |  | |
| --- | --- | --- | --- | --- | --- | --- | --- |
| 62. | | You didn’t have enough to eat.  Please check all ages that apply.   \| 1 \| 2 \| 3 \| 4 \| 5 \| 6 \| 7 \| 8 \| 9 \| 10 \| 11 \| 12 \| 13 \| 14 \| 15 \| 16 \| 17 \| 18 \| \| --- \| --- \| --- \| --- \| --- \| --- \| --- \| --- \| --- \| --- \| --- \| --- \| --- \| --- \| --- \| --- \| --- \| --- \| \|  \|  \|  \|  \|  \|  \|  \|  \|  \|  \|  \|  \|  \|  \|  \|  \|  \|  \| | 🔿  Yes_1_ | | 🔿  No_0_ | |  |
| 63. | | You had to wear dirty clothes.  Please check all ages that apply.   \| 1 \| 2 \| 3 \| 4 \| 5 \| 6 \| 7 \| 8 \| 9 \| 10 \| 11 \| 12 \| 13 \| 14 \| 15 \| 16 \| 17 \| 18 \| \| --- \| --- \| --- \| --- \| --- \| --- \| --- \| --- \| --- \| --- \| --- \| --- \| --- \| --- \| --- \| --- \| --- \| --- \| \|  \|  \|  \|  \|  \|  \|  \|  \|  \|  \|  \|  \|  \|  \|  \|  \|  \|  \| | 🔿  Yes_1_ | | 🔿  No_0_ | |  |
| 64. | | You were left unsupervised at an age or in situations when you should have been supervised.  Please check all ages that apply.   \| 1 \| 2 \| 3 \| 4 \| 5 \| 6 \| 7 \| 8 \| 9 \| 10 \| 11 \| 12 \| 13 \| 14 \| 15 \| 16 \| 17 \| 18 \| \| --- \| --- \| --- \| --- \| --- \| --- \| --- \| --- \| --- \| --- \| --- \| --- \| --- \| --- \| --- \| --- \| --- \| --- \| \|  \|  \|  \|  \|  \|  \|  \|  \|  \|  \|  \|  \|  \|  \|  \|  \|  \|  \| | 🔿  Yes_1_ | | 🔿  No_0_ | |  |
| 65. | | You felt that you had to shoulder adult responsibilities.  Please check all ages that apply.   \| 1 \| 2 \| 3 \| 4 \| 5 \| 6 \| 7 \| 8 \| 9 \| 10 \| 11 \| 12 \| 13 \| 14 \| 15 \| 16 \| 17 \| 18 \| \| --- \| --- \| --- \| --- \| --- \| --- \| --- \| --- \| --- \| --- \| --- \| --- \| --- \| --- \| --- \| --- \| --- \| --- \| \|  \|  \|  \|  \|  \|  \|  \|  \|  \|  \|  \|  \|  \|  \|  \|  \|  \|  \| | 🔿  Yes_1_ | | 🔿  No_0_ | |  |
| 66. | | You felt that your family was under severe financial pressure.  Please check all ages that apply.   \| 1 \| 2 \| 3 \| 4 \| 5 \| 6 \| 7 \| 8 \| 9 \| 10 \| 11 \| 12 \| 13 \| 14 \| 15 \| 16 \| 17 \| 18 \| \| --- \| --- \| --- \| --- \| --- \| --- \| --- \| --- \| --- \| --- \| --- \| --- \| --- \| --- \| --- \| --- \| --- \| --- \| \|  \|  \|  \|  \|  \|  \|  \|  \|  \|  \|  \|  \|  \|  \|  \|  \|  \|  \| | 🔿  Yes_1_ | | 🔿  No_0_ | |  |
| 67. | | One or more individuals kept important secrets or facts from you.  Please check all ages that apply.   \| 1 \| 2 \| 3 \| 4 \| 5 \| 6 \| 7 \| 8 \| 9 \| 10 \| 11 \| 12 \| 13 \| 14 \| 15 \| 16 \| 17 \| 18 \| \| --- \| --- \| --- \| --- \| --- \| --- \| --- \| --- \| --- \| --- \| --- \| --- \| --- \| --- \| --- \| --- \| --- \| --- \| \|  \|  \|  \|  \|  \|  \|  \|  \|  \|  \|  \|  \|  \|  \|  \|  \|  \|  \| | 🔿  Yes_1_ | | 🔿  No_0_ | |  |
| 68. | | Your parents were separated.  Please check all ages that apply.   \| 1 \| 2 \| 3 \| 4 \| 5 \| 6 \| 7 \| 8 \| 9 \| 10 \| 11 \| 12 \| 13 \| 14 \| 15 \| 16 \| 17 \| 18 \| \| --- \| --- \| --- \| --- \| --- \| --- \| --- \| --- \| --- \| --- \| --- \| --- \| --- \| --- \| --- \| --- \| --- \| --- \| \|  \|  \|  \|  \|  \|  \|  \|  \|  \|  \|  \|  \|  \|  \|  \|  \|  \|  \| | 🔿  Yes_1_ | | 🔿  No_0_ | |  |
| 69. | | Your parents were divorced.  Please check all ages that apply.   \| 1 \| 2 \| 3 \| 4 \| 5 \| 6 \| 7 \| 8 \| 9 \| 10 \| 11 \| 12 \| 13 \| 14 \| 15 \| 16 \| 17 \| 18 \| \| --- \| --- \| --- \| --- \| --- \| --- \| --- \| --- \| --- \| --- \| --- \| --- \| --- \| --- \| --- \| --- \| --- \| --- \| \|  \|  \|  \|  \|  \|  \|  \|  \|  \|  \|  \|  \|  \|  \|  \|  \|  \|  \| | 🔿  Yes_1_ | | 🔿  No_0_ | |  |
| 70. | | A parent or other important parental figure died.  Please check all ages that apply.   \| 1 \| 2 \| 3 \| 4 \| 5 \| 6 \| 7 \| 8 \| 9 \| 10 \| 11 \| 12 \| 13 \| 14 \| 15 \| 16 \| 17 \| 18 \| \| --- \| --- \| --- \| --- \| --- \| --- \| --- \| --- \| --- \| --- \| --- \| --- \| --- \| --- \| --- \| --- \| --- \| --- \| \|  \|  \|  \|  \|  \|  \|  \|  \|  \|  \|  \|  \|  \|  \|  \|  \|  \|  \| | 🔿  Yes_1_ | | 🔿  No_0_ | |  |
| 71. | | You had to spend time living in two or more households.  Please check all ages that apply.   \| 1 \| 2 \| 3 \| 4 \| 5 \| 6 \| 7 \| 8 \| 9 \| 10 \| 11 \| 12 \| 13 \| 14 \| 15 \| 16 \| 17 \| 18 \| \| --- \| --- \| --- \| --- \| --- \| --- \| --- \| --- \| --- \| --- \| --- \| --- \| --- \| --- \| --- \| --- \| --- \| --- \| \|  \|  \|  \|  \|  \|  \|  \|  \|  \|  \|  \|  \|  \|  \|  \|  \|  \|  \| | 🔿  Yes_1_ | | 🔿  No_0_ | |  |
| 72. | | You lived in foster care.  Please check all ages that apply.   \| 1 \| 2 \| 3 \| 4 \| 5 \| 6 \| 7 \| 8 \| 9 \| 10 \| 11 \| 12 \| 13 \| 14 \| 15 \| 16 \| 17 \| 18 \| \| --- \| --- \| --- \| --- \| --- \| --- \| --- \| --- \| --- \| --- \| --- \| --- \| --- \| --- \| --- \| --- \| --- \| --- \| \|  \|  \|  \|  \|  \|  \|  \|  \|  \|  \|  \|  \|  \|  \|  \|  \|  \|  \| | 🔿  Yes_1_ | | 🔿  No_0_ | |  |
| 73. | | People in your family looked out for each other.  Please check all ages that apply.   \| 1 \| 2 \| 3 \| 4 \| 5 \| 6 \| 7 \| 8 \| 9 \| 10 \| 11 \| 12 \| 13 \| 14 \| 15 \| 16 \| 17 \| 18 \| \| --- \| --- \| --- \| --- \| --- \| --- \| --- \| --- \| --- \| --- \| --- \| --- \| --- \| --- \| --- \| --- \| --- \| --- \| \|  \|  \|  \|  \|  \|  \|  \|  \|  \|  \|  \|  \|  \|  \|  \|  \|  \|  \| | 🔿  Yes_1_ | | 🔿  No_0_ | |  |
| 74. | | People in your family felt close to each other.  Please check all ages that apply.   \| 1 \| 2 \| 3 \| 4 \| 5 \| 6 \| 7 \| 8 \| 9 \| 10 \| 11 \| 12 \| 13 \| 14 \| 15 \| 16 \| 17 \| 18 \| \| --- \| --- \| --- \| --- \| --- \| --- \| --- \| --- \| --- \| --- \| --- \| --- \| --- \| --- \| --- \| --- \| --- \| --- \| \|  \|  \|  \|  \|  \|  \|  \|  \|  \|  \|  \|  \|  \|  \|  \|  \|  \|  \| | 🔿  Yes_1_ | | 🔿  No_0_ | |  |
| 75. | | Your family was a source of strength and support.  Please check all ages that apply.   \| 1 \| 2 \| 3 \| 4 \| 5 \| 6 \| 7 \| 8 \| 9 \| 10 \| 11 \| 12 \| 13 \| 14 \| 15 \| 16 \| 17 \| 18 \| \| --- \| --- \| --- \| --- \| --- \| --- \| --- \| --- \| --- \| --- \| --- \| --- \| --- \| --- \| --- \| --- \| --- \| --- \| \|  \|  \|  \|  \|  \|  \|  \|  \|  \|  \|  \|  \|  \|  \|  \|  \|  \|  \| | 🔿  Yes_1_ | | 🔿  No_0_ | |  |
