# Supplementary material for: The ‘Maltreatment and Abuse Chronology of Exposure’ (MACE) Scale for the Retrospective Assessment of Abuse and Neglect During Development
Source: PLoS One. 2015 Feb 25;10(2):e0117423. doi: 10.1371/journal.pone.0117423 (PMC4340880; doi:10.1371/journal.pone.0117423)
Supplement: S8 File — (PDF) [file pone.0117423.s017.pdf]

Package 'MACEscore'

December 29, 2014

Type Package

Title Scoring Package for Maltreatment and Abuse Chronology of Exposure Scales

Version 1.1

Date 2014-11-17

Author Martin H. Teicher

Maintainer Martin H. Teicher <martin_teicher@hms.harvard.edu>

Description Scores Maltreatment and Abuse Chronology of Exposure Scale (MACE), Maltreatment and Abuse Exposure Scale (MAES) and original developmental version of MACE (MACE-X). Also includes routines for summarizing results and checking and correcting common entry errors.

License GPL-2

R topics documented:

MACEscore-package 2
auto_check_mace 3
check_entries 5
check_ER_MD 6
check_ever_for_ages_with_exposure 7
check_peer_date 7
correct_missing_ever_values 8
generate_filename_fx 9
mace.calc 9
mace.score 11
mace_sample 13
mace_sample_data 13
mace_type_fx 13
mace_x_sample 14
maes.calc 14
maes_sample 15
spank_yr_check 16
summarize 17

Index **19**

MACEscore-package *Scoring Package for Maltreatment and Abuse Chronology of Exposure Scales*

Description

Provides routines for scoring MACE (52 Questions, 988 items), MACE-X (75 Main Questions, 1728 items), and MAES (Maltreatment and Abuse Exposure Scale; 52 questions, 52 items). Also includes functions for checking for possible input or entry errors, and for correcting common input or entry mistakes, plus sample MACE, MACE-X and MAES data for scoring.

Details

Package:	MACEscore
Type:	Package
Version:	1.1
Date:	2014-11-17
License:	GPL-2

Author(s)

Martin H. Teicher <martin_teicher@hms.harvard.edu>

References

Teicher,M. H. & Parigger, A. The Maltreatment and Abuse Chronology of Exposure (MACE) Scale for the Retrospective Assessment of Maltreatment During Development. PLoS ONE (in press).

See Also

maes.calc, mace.calc, mace.score, summarize, auto_check_mace, check_entries, check_ER_MD, check_ever_for_ages_with_exposure, correct_missing_ever_values, check_peer_date, spank_yr_check

Examples

```
## Load sample data
data(mace_sample_data)

## See how MACE, MACE-X and MAES data must be formatted for correct scoring
names(mace_sample)

names(mace_x_sample)

names(maes_sample)

## Score MACE  (same command for MACE_X or MAES)
## Results are provided in a data frame
## Note: data frame passed to scoring function must have correct number of columns from
##   start.col to last column.  Additional data columns can be contained prior to start
```

```
##  column but not after last required data column

x<-mace.score(mace_sample,start.col=3)

## Summarize basic results
summarize(x)

## Provide more detailed summary of MACE and MACE_x scores
summarize(x,form="long")
```

`auto_check_mace` *Check items on MACE or MACE-X for different types of entry errors*

Description

Checks automatically for different types of entry errors recognized to date. Each type of entry error can be detected through their own function call. This routine calls each of the functions in turn. Further, flags can be set to automatically correct detected errors based on available options. Use with care.

Usage

```
auto_check_mace(fp_data, start.col, auto_fix_pos_age_o_ever = TRUE,
auto_fix_missing_ever = TRUE, auto_fix_pos_ever_0_yrs = NA, auto_fix_peer_data = TRUE,
auto_fix_spank = TRUE, auto_fix_ER = FALSE)
```

Arguments

`fp_data` MACE or MACE-X data frame suitable for scoring

`start.col` Integer value indicating column in fp_data where MACE or MACE item responses begin

`auto_fix_pos_age_o_ever`
Flag to indicate whether to rescore Ever <- 1 in instance where Ever==0 but subject indicated exposure at one or more ages.

`auto_fix_missing_ever`
Flag to indicate whether to rescore Ever==NA as 1 or 0 based on whether they reported any exposure on years 1-18.

`auto_fix_pos_ever_0_yrs`
Flag with options TRUE, FALSE or NA. If TRUE will rescore Ever from 1 to 0 if all years are 0 (years override ever). If NA selected will rescore all years from 0 to NA when all years == 0 but Ever==1 (ever overrides years.)

`auto_fix_peer_data`
Flag to indicate whether to rescore MACE-X items that inquire about various forms of peer emotional or physical abuse based on response to follow-up question asking if the peer was a date. Some subjects left the peer responses blank when indicating a positive dating event. This function rescores the peer event as positive when the date event was scored positive.

`auto_fix_spank` Flag indicates whether to correct for possible misunderstanding of Question 9 - "Spanked you on your buttocks, arms or legs" as subjects sometimes answered NO for ages when they answered YES to Question 10 - "Spanked you on your bare (unclothed) buttocks" or Question 11 - "Spanked you with an object such as a strap, belt, brush, paddle, rod, etc."

auto_fix_ER
: MACE item 45 asks subjects to indicate whether - 'One or more individuals in your family were there to take you to the doctor or Emergency Room if the need ever arose, or would have if needed.' Some subject have indicated YES, but only indicated years in which the need actually arose, rather than all years in which a family member would have done so if required. If flag is set to TRUE will rescore ER_MD years based on response to item 44 - 'One or more individuals in your family were there to take care of you and protect you' and item 51 - 'People in your family looked out for each other.' An ER_MD year will be rescored from 0 to 1 if response to items 44 and 51 were both true for that year.

Details

Lists number of potential errors of each type and subjects / items in question.

Value

data frame
: Data frame with MACE or MACE-X items including corrected items

Author(s)

Martin H. Teicher <martin_teicher@hms.harvard.edu>

See Also

check_entries, check_ER_MD, correct_missing_ever_values, check_peer_date, spank_yr_check, check_ever_for_ages_with_exposure

Examples

```
## Load sample data
data(mace_sample_data)

## Produce some artificial discrepancies
mace_x_sample2<-mace_x_sample
mace_x_sample2[1,21]<-0    # Zero childhood/ever x positive years discrepancy
mace_x_sample2[2,40]<-1    # positive childhood/ever x zero years discrepancy
mace_x_sample2[3,687]<-NA    # NA childhood/ever x zero years discrepancy
mace_x_sample2[4,190:194]<-0    # Spank discrepancies
mace_x_sample2[7,884:885]<-0    # Peer / date discrepancies
mace_x_sample2[8,1428:1432]<-0         # ER_MD discrepancies

# Autocheck and correct raw MACE-X data
mace_frame<-auto_check_mace(mace_x_sample2, start.col=3, auto_fix_pos_age_o_ever=TRUE,
auto_fix_missing_ever=TRUE, auto_fix_pos_ever_0_yrs=NA, auto_fix_peer_data=TRUE,
auto_fix_spank=TRUE, auto_fix_ER=FALSE)
```

`check_entries` *Check items on MACE or MACE-X for possible entry errors*

Description

Checks for incongruities in response to 'CHILDHOOD" or 'EVER' item versus individual age items. Reports instances when subjects indicated exposure to particular event (EVER == 1) but failed to indicate ages when exposure occurred.

Usage

```
check_entries(mace_frame, start.col = 3)
```

Arguments

`mace_frame`	MACE or MACE-X data frame suitable for scoring
`start.col`	Integer value indicating column in data frame where MACE or MACE item responses begin

Value

`sub.check.scores`
: Vector indicating number of incongruities per subject

`pos.ever.zero.years`
: Lists each subject with an incongruous response characterized by EVER==1 and all years == 0 and indicates which specific items were entered in this way

`pos.ever.na.years`
: Lists each subject with an incongruous response characterized by EVER==1 and all years == NA and indicates which specific items were entered in this way

`mace_frame2`
: Returns revised mace_frame where EVER==1 is rescored as EVER==0 when all years were scored as 0. In this option years override ever.

`mace_frame3`
: Returns revised mace_frame in which instances when EVER==1 but all years are scored 0 leads to years being rescored as NA. In this option ever override years.

Examples

```
## Load sample data
data(mace_sample_data)

## Introduce some discrepancies
mace_sample2 <- mace_sample
mace_sample2[4,c(230,249)]<-1

## Check data for common errors
q<-check_entries(mace_sample2,start.col=3)
q$pos.ever.zero.years # Subject indicated that experience occurred but indicated zero
# (no) for all years
q$pos.ever.na.years # Subject indicated that experience occurred but indicated NA
# for all years
```

check_ER_MD *Check and correct possible misunderstanding of MACE item 45*

Description

MACE item 45 asks subjects to indicate whether - 'One or more individuals in your family were there to take you to the doctor or Emergency Room if the need ever arose, or would have if needed.' Some subject have indicated YES, but only indicated years in which the need actually arose, rather than all years in which a family member would have done so if required. This function corrects this possible misunderstanding.

Usage

```
check_ER_MD(mace_frame, start.col, verbose = TRUE)
```

Arguments

mace_frame	MACE or MACE-X data frame suitable for scoring
start.col	Integer value indicating column in data frame where MACE or MACE-X item responses begin.
verbose	TRUE / FALSE flag to have routine indicate subjects with possible ER_MD discrepancies.

Details

Response to item 45 is modified based on response to item 44 - 'One or more individuals in your family were there to take care of you and protect you' and item 51 - 'People in your family looked out for each other.' Item 45 is rescored as positive during each year in which subjects indicated that both item 44 and item 51 were true.

Value

data frame	Data frame with MACE-X items including corrected peer items

Examples

```
## Load sample data
data(mace_sample_data)

## Produce some artificial discrepancies
mace_x_sample2<-mace_x_sample
mace_x_sample2[8,1428:1432]<-0        # ER_MD discrepancies

## Check for possible misunderstanding of item 45 - availability of family member to take
## you to ER or to MD if need was to arise
mace_x_sample3 <- check_ER_MD(mace_x_sample2,start.col=3,verbose=TRUE)
```

check_ever_for_ages_with_exposure
Check missing 'EVER' responses with exposure on MACE or MACE-X

Description

Some subjects have indicated that specific events occurred during one or more childhood years, but failed to answer whether the event ever occurred. This function checks for and updates 'EVER' responses in such instances.

Usage

```
check_ever_for_ages_with_exposure(mace_frame, start.col = 3)
```

Arguments

mace_frame	Data frame containing responses to MACE or MACE-X items suitable for scoring
start.col	Integer value for column in data frame in which MACE or MACE-X items begin

Value

data frame	Data frame with MACE or MACE-X items including corrected 'EVER' items

Examples

```
## Load sample data
data(mace_sample_data)

## Produce some artificial discrepancies
mace_x_sample2<-mace_x_sample
mace_x_sample2[1,21]<-0    # Zero childhood/ever x positive years discrepancy
mace_x_sample2[4,687]<-NA    # NA childhood/ever x zero years discrepancy

## If MACE or MACE-X, check for discrepancies where EVER is negative or NA
## but specific years positive
hold <- check_ever_for_ages_with_exposure(mace_x_sample2,start.col=3)
```

check_peer_date
Checks and corrects responses to peer items based on dating items on MACE-X

Description

The MACE-X has items that inquire about various forms of peer emotional or physical abuse. For each question there is a follow-up question asking if the peer was a date. Some subjects have left the peer responses blank when indicating a positive dating event. This function rescores the peer event as positive when the date event was scored positive.

Usage

```
check_peer_date(mace_frame, start.col,verbose=TRUE)
```

Arguments

mace_frame	MACE-X data frame suitable for scoring
start.col	Column in data frame where MACE-X item responses begin
verbose	TRUE / FALSE flag to indicate whether routine should print out subject names and row numbers with discrepancies.

Value

data frame	Data frame with MACE-X items including corrected peer items

Examples

```
## Load sample data
data(mace_sample_data)

## Produce some artificial discrepancies
mace_x_sample2<-mace_x_sample
mace_x_sample2[7,884:885]<-0     # Peer / date discrepancies

## If MACE-X, correct peer exposure if peer year negative but date year positive
hold <- check_peer_date(mace_x_sample2,start.col=3,verbose=TRUE)
```

---

correct_missing_ever_values

Correct missing 'EVER' responses on MACE or MACE-X

Description

Depricated function used when an earlier electronic version of the MACE-X failed to output results for 'EVER' columns. This function rescored the 'EVER' column based on responses to the same item at specific ages.

Usage

```
correct_missing_ever_values(mace_frame, start.col = 3, verbose = FALSE)
```

Arguments

mace_frame	Data frame containing responses to MACE or MACE-X items suitable for scoring
start.col	Column in data frame in which MACE or MACE-X items begin
verbose	Flag to indicate whether to enumerate ever items and subjects being corrected

Value

data frame	Data frame with MACE or MACE-X items including corrected 'EVER' items

Examples

```
## Load sample data
data(mace_sample_data)

## Introduce some missing EVER items
mace_sample2 <- mace_sample
mace_sample2[4,c(230,249)]<-NA

## If MACE or MACE-X, score EVER based on responses across ages 1-18
mace_sample3 <- correct_missing_ever_values(mace_sample2,start.col=3, verbose = TRUE)
```

---

generate_filename_fx *Generate file name for saving MACE scores*

Description

Generates a generic file name for saving MACE, MACE-X or MAES scores which includes time and date.

Usage

```
generate_filename_fx(pathe, prefix = NULL, xstring, suffix = ".csv")
```

Arguments

pathe	Path to directory where file will be saved
prefix	Initial text of file name
xstring	Second text component of file name (e.g., MACE_scores)
suffix	Final text component of file name - typically extension

Value

text	Full file path and name which includes date and time

Author(s)

Martin H. Teicher <martin_teicher@hms.harvard.edu>

---

mace.calc *Scoring MACE and MACE-X*

Description

Function for scoring the Maltreatment and Abuse Chronology of Exposure Scale (MACE) or longer developmental version (MACE-X)

Usage

```
mace.calc(mace_frame, start.col, type = "mace")
```

Arguments

`mace_frame`	Raw MACE or MACE-X data.frame suitable for scoring with 988 columns from start.col to end for MACE and 1728 columns for MACE-X
`start.col`	Integer variable indicating column in data.frame where MACE or MACE-X items start
`type`	Text indicating `"mace"` or `"mace-x"`

Details

See `mace_sample` and `mace_x_sample` for examples of how columns need to be ordered for correct scoring

Value

`data.frame`	Data frame with 240 columns for MACE and 243 columns for MACE-X

subj:	- 1st column of mace_frame
columns 2-19:	- Severity of sexual abuse at ages 1 - 18
column 20:	- Overall severity of childhood sexual abuse
columns 21-38:	- Severity of parental verbal abuse at ages 1 - 18
column 39:	- Overall severity of exposure to parental verbal abuse
columns 40-57:	- Severity of parental non-verbal emotional abuse at ages 1 - 18
column 58:	- Overall severity of exposure to parental non-verbal emotional abuse
columns 59-76:	- Severity of parental physical maltreatment at ages 1 - 18
column 77:	- Overall severity of exposure to parental physical maltreatment
columns 78-95:	- Severity of witnessing intraparental violence at ages 1 - 18
column 96:	- Overall severity of exposure to intraparental violence
columns 97-114:	- Severity of witnessing violence to siblings at ages 1 - 18
column 115:	- Overall severity of exposure to witnessing violence to siblings
columns 116-133:	- Severity of peer emotional abuse at ages 1 - 18
column 134:	- Overall severity of exposure to peer emotional abuse
columns 135-152:	- Severity of peer physical bullying at ages 1 - 18
column 153:	- Overall severity of exposure to peer physical bullying
columns 154-171:	- Severity of emotional neglect at ages 1 - 18
column 172:	- Overall severity of exposure to emotional neglect
columns 173-190:	- Severity of physical neglect at ages 1 - 18
column 191:	- Overall severity of physical neglect
columns 192-209:	- Number of different types of maltreatment experienced at ages 1-18
columns 210-227:	- Overall severity of exposure to maltreatment at ages 1-18
column 228:	- Number of different types of maltreatment experienced during childhood MACE_MUTI
column 229:	- Overall severity of exposure to childhood maltreatment MACE_SUM
columns 230-239:	- Overall severity of exposure to sexual abuse, parental verbal abuse,
-	non-verbal emotional abuse, physical maltreatment, witnessing intraparental
-	violence, witnessing violence to sibs, peer emotional abuse, peer physical abuse,
-	emotional neglect, physical neglect (same as columns 20, 39, 58, 77,96, 115,
-	134,153,172,191)
column 240:	- Number of different types of familial maltreatment experienced
-	includes intrafamilial CSA, PVA, NVEA, PPhysM, IPV, WSibA, EN, PN
MACE-X ONLY	
column 241:	- Number of different events that produced feelings of helplessness
column 242:	- Number of different events that produced feelings of terror
column 243:	- Number of different events that produced feeling of hopeless and/or terror

Author(s)

Martin H. Teicher <martin_teicher@hms.harvard.edu>

References

Teicher,M. H. & Parigger, A. The Maltreatment and Abuse Chronology of Exposure (MACE) Scale for the Retrospective Assessment of Maltreatment During Development. PLoS ONE (in press).

See Also

`mace.score`, `mace.calc`

Examples

```
## Load sample data
data(mace_sample_data)

## See how MACE and MACE-X data must be formatted for correct scoring
names(mace_sample)
names(mace_x_sample)

## Score MACE
## Note: data frame passed to scoring function must have correct number of columns (988 or 1729)
##   from start.col to last column.  Additional information can be contained prior to start
##  column but not after last required data column

x1<-mace.score(mace_sample,start.col=3)
x2<-mace.score(mace_x_sample,start.col=3)
## or
x1<-mace.calc(mace_sample,start.col=3,type="mace")
x2<-mace.calc(mace_x_sample,start.col=3,type="mace-x")

## Summarize results - short format
summarize(x1)
summarize(x2)

## Summarize results - long format
summarize(x1,"long")
summarize(x2,"long")
```

`mace.score` *Wrapper function for calculating MACE, MACE-X or MAES scores*

Description

Calls appropriate routines for scoring Maltreatment and Abuse Chronology of Exposure Scale (MACE, 52 Questions, 988 items), MACE-X (75 Main Questions, 1728 items), and MAES (Maltreatment and Abuse Exposure Scale; 52 questions, 52 items)

Usage

```
mace.score(mace_frame, start.col = 3)
```

Arguments

`mace_frame`	Data frame for MACE, MACE-X or MAES suitable for scoring
`start.col`	Column in data frame where MACE, MACE-X or MAES items begin

Details

It is critical that items in the data frame to be scored have been entered in the exact sequence shown in the sample data for the MACE, MACE-X or MAES. The program does not check that they have. It only checks that the data frame contains the exact number of columns required for the scoring. Data frames with more than or less than the required number of columns (from start.col to end) will not be scored. The required number of columns is 52 for MAES, 988 for MACE and 1728 for MACE-X.

Value

`data.frame`	Data frame of scored values. Number of columns in data frame depends on type of test scored. For MAES the data frame contains 24 columns, for MACE 240 columns, for MACE-X 243. See mace.cal and maes.cal for description of columns in data frame.

Warning

Make certain that data to be scored has been entered in the exact correct sequence indicated in the sample data.

Author(s)

Martin H. Teicher <martin_teicher@hms.harvard.edu>

References

Teicher,M. H. & Parigger, A. The Maltreatment and Abuse Chronology of Exposure (MACE) Scale for the Retrospective Assessment of Maltreatment During Development. PLoS ONE (in press).

See Also

`maes.calc`, `mace.calc`, `summarize`

Examples

```
## Load sample data
data(mace_sample_data)

## See how MACE, MACE-X and MAES data must be formatted for correct scoring
names(mace_sample)
names(mace_x_sample)
names(maes_sample)

## Score MACE
x1<-mace.score(mace_sample,start.col=3)

## Score MACE-X
x2<-mace.score(mace_x_sample,start.col=3)
```

```
## Score MAES
x3<-mace.score(maes_sample,start.col=3)

## See results
summarize(x1)
summarize(x2)
summarize(x3)
```

`mace_sample` *Sample data to check routines for scoring MACE*

Description

Data set with MACE item scores for 9 subjects.

`mace_sample_data` *Sample data to check routines for scoring MACE, MACE-X and MAES*

Description

Contains `maes_sample`, `mace_sample` and `mace_x_sample` data sets which represent MAES, MACE and MACE-X item scores for 9 subjects.

`mace_type_fx` *Internal function for determining if data frame is MAES, MACE or MACE-X*

Description

Internal function determines if data frame is suitable for scoring as MACE, MACE-X or MAES based on number of columns. At this stage no further checks are applied. MAES requires exactly 52 columns from designated start.col to last column. MACE requires 988 and MACE-x 1728.

Usage

```
mace_type_fx(mace_frame, start.col)
```

Arguments

`mace_frame`	Data frame of responses to MAES, MACE or MACE-X test items
`start.col`	Integer variable indicating column in data.frame where MAES, MACE or MACE-X items start

Value

`text string`	"mace", "mace-x", "maes" or NA

`mace_x_sample` *Sample data to check routines for scoring MACE-X*

Description

Data set with MACE-X item scores for 9 subjects.

`maes.calc` *Scoring Maltreatment and Abuse Exposure Scale (MAES)*

Description

Function for scoring the Maltreatment and Abuse Exposure Scale (MAES)

Usage

```
maes.calc(mace_frame, start.col)
```

Arguments

`mace_frame`	Raw MAES data.frame suitable for scoring with 52 columns from start.col to end
`start.col`	Integer variable indicating column in data.frame where MAES items start

Details

See maes_sample for example of how columns need to be ordered for correct scoring

Value

`data.frame`	Data frame with 24 columns containing scored results

subj:	- 1st column of raw data.frame
MAES_SexAb_SUM:	- severity of exposure to childhood sexual abuse
MAES_PVA_SUM:	- severity of exposure to parental verbal abuse
MAES_NVEA_SUM:	- severity of exposure to parental non-verbal emotional abuse
MAES_PPhysM_SUM:	- severity of exposure to parental physical Maltreatment
MAES_IPV_SUM:	- severity of exposure to witnessing intraparental violence
MAES_WSibA_SUM:	- severity of exposure to witnessing violence to siblings
MAES_PeerVA_SUM:	- severity of exposure to peer emotional abuse
MAES_PeerPhys_SUM:	- severity of exposure to peer physical bullying
MAES_EN_SUM:	- severity of exposure to emotional neglect
MAES_PN_SUM:	- severity of exposure to physical neglect
MAES_MULTI:	- number of different types of maltreatment experienced
MAES_SUM:	- overall severity of exposure to maltreatment
MAES_SexAb_MULTI:	- 1 indicates above threshold exposure to childhood sexual abuse
MAES_PVA_MULTI:	- 1 indicates above threshold exposure to parental verbal abuse
MAES_NVEA_MULTI:	- 1 indicates above threshold exposure to parental non-verbal emotional abuse
MAES_PPhysM_MULTI:	- 1 indicates above threshold exposure to parental physical maltreatment

MAES_IPV_MULTI:	- 1 indicates above threshold exposure to witnessing intraparental violence
MAES_WSibA_MULTI:	- 1 indicates above threshold exposure to witnessing violence to siblings
MAES_PeerVA_MULTI:	- 1 indicates above threshold exposure to peer emotional abuse
MAES_PeerPhys_MULTI:	- 1 indicates above threshold exposure to peer physical bullying
MAES_EN_MULTI:	- 1 indicates above threshold exposure to emotional neglect
MAES_PN_MULTI:	- 1 indicates above threshold exposure to physical neglect
intrafamilial_MAES:	- number of different types of familial maltreatment experienced
-	includes intrafamilial CSA, PVA, NVEA, PPhysM, IPV, WSibA, EN, PN

Author(s)

Martin H. Teicher <martin_teicher@hms.harvard.edu>

References

Teicher,M. H. & Parigger, A. The Maltreatment and Abuse Chronology of Exposure (MACE) Scale for the Retrospective Assessment of Maltreatment During Development. PLoS ONE (in press).

See Also

mace.score, mace.calc

Examples

```
## Load sample data
data(mace_sample_data)

## See how MAES data must be formatted for correct scoring
names(maes_sample)

## Score MAES
## Note: data frame passed to scoring function must have correct number of columns (52) from
##   start.col to last column.  Additional information can be contained prior to start
##  column but not after last required data column
x<-mace.score(maes_sample,start.col=3)
## or
x<-maes.calc(maes_sample,start.col=3)

## Summarize results
summarize(x)
```

`maes_sample` *Sample data to check routines for scoring MAES*

Description

Data set with MAES item scores for 9 subjects.

spank_yr_check *Check spank items*

Description

Checks and corrects possible misunderstanding of Question 9 - "Spanked you on your buttocks, arms or legs" as subjects sometimes answer NO for ages when they answered YES to Question 10 - "Spanked you on your bare (unclothed) buttocks" or Question 11 - "Spanked you with an object such as a strap, belt, brush, paddle, rod, etc."

Usage

```
spank_yr_check(mace_frame, start.col, verbose = TRUE)
```

Arguments

mace_frame	MACE or MACE-X raw data.frame suitable for scoring
start.col	Column number of first MACE or MACE-x item - "Swore_1"
verbose	TRUE / FALSE flag to indicate whether routine should print out subject names and row numbers with discrepancies.

Value

data.frame	Returns corrected MACE or MACE-X raw data.frame suitable for scoring with start.col = 3

Author(s)

Martin H. Teicher <martin_teicher@hms.harvard.edu

Examples

```
## Load sample data
data(mace_sample_data)

## Produce some artificial discrepancies
mace_x_sample2<-mace_x_sample
mace_x_sample2[4,190:194]<-0    # Spank discrepancies

## Correct if spank year negative but spank_bare or spank_object are positive
mace_x_sample_revised<-spank_yr_check(mace_x_sample2,start.col=3,verbose=TRUE)
```

summarize *Summarize scored MACE results*

Description

Provides short or more complete summary of results of MACE, MACE-X or MAES tests

Usage

```
summarize(x, form = "short")
```

Arguments

x	A MACE, MACE-X or MAES data.frame provided through mace.score function
form	Text string with default="short". Any other text string will select more complete format.

Value

overall.severity	MACE_MULT and MACE_SUM values for subjects
types.abuse	Overall sexab, pva, nvea, pphys, wipv, w_sib, peerEA, peerPhys, en, pn scores
form="long"	Provides these additional results...
sexab.age	Sexual abuse by age
pva.age	Peer Verbal abuse by age
nvea.age	Non-verbal emotional abuse by age
pphysmal.age	Parental physical abuse by age
wipv.age	Witnessing intraparental violence by age
wsib.age	Witnessing abuse to siblings by age
peerEA.age	Peer emotional abuse by age
peerPhys.age	Peer physical abuse by age
en.age	Emotional neglect by age
pn.age	Physical neglect by age
mult.age	MACE Multi by age
sum.age	MACE Sum by age

Author(s)

Martin H. Teicher <martin_teicher@hms.harvard.edu>

Examples

```
## Load sample data
data(mace_sample_data)

## Score MACE data
x<-mace.score(mace_sample,start.col=3)

## Summarize basic results
summarize(x)

## Provide more detailed summary of MACE and MACE_x scores
summarize(x,form="long")
```

Index

∗Topic **datasets**
mace_sample, 13
mace_sample_data, 13
mace_x_sample, 14
maes_sample, 15
∗Topic **list**
summarize, 17
∗Topic **package**
MACEscore-package, 2

auto_check_mace, *2*, 3

check_entries, *2*, *4*, 5
check_ER_MD, *2*, *4*, 6
check_ever_for_ages_with_exposure, *2*, *4*, 7
check_peer_date, *2*, *4*, 7
correct_missing_ever_values, *2*, *4*, 8

generate_filename_fx, 9

mace.calc, *2*, 9, *11, 12*, *15*
mace.score, *2*, *11*, 11, *15*
mace_sample, 13
mace_sample_data, 13
mace_type_fx, 13
mace_x_sample, 14
MACEscore (MACEscore-package), 2
MACEscore-package, 2
maes.calc, *2*, *12*, 14
maes_sample, 15

spank_yr_check, *2*, *4*, 16
summarize, *2*, *12*, 17
